# Upcycling fish scales through heating for steganography and Rhodamine B adsorption application

**DOI:** 10.1038/s41467-023-42080-1

**Published:** 2023-10-16

**Authors:** Malcolm Miao Geng Sow, Zheng Zhang, Chorng Haur Sow, Sharon Xiaodai Lim

**Affiliations:** 1 NUS High School of Mathematics and Science, 20 Clementi Avenue 1, Singapore, 129957 Singapore; 2 Institute of Materials Research and Engineering (IMRE), Agency for Science, Technology and Research (A*STAR), 2 Fusionopolis Way, Innovis #08-03, Singapore, 138634 Singapore; 3 Department of Physics, National University of Singapore, 2 Science Drive 3, Singapore, 117542 Singapore

**Keywords:** Synthesis and processing, Optical materials, Biopolymers

## Abstract

With increasing population and limited resources, a potential route for improving sustainability is increased reuse of waste materials. By re-looking at wastes, interesting properties and multifunctionalities can be discovered in materials previously explored. Despite years of research on bio-compatible fish scales, there is limited study on the fluorescence property of this abundant waste material. Controlled denaturation of collagen and introduction of defects can serve as a means to transform the fluorescence property of these fish scale wastes while providing more adsorption sites for pollutant removal, turning multifunctional fish scales into a natural steganographic material for transmitting text and images at both the macroscopic and microscopic levels and effectively removing Rhodamine B pollutants (91 % removal) within a short contact time (10 minutes). Our work offers a glimpse into the realm of engineering defects-induced fluorescence in natural material with potential as bio-compatible fluorescence probes while encouraging multidimensional applicability to be established in otherwise overlooked waste resources.

## Abstract (web summary)

Waste materials including fish scales are increasingly being developed for alternative applications, but simple processing is required. Here, the authors report the treatment of fish scales to give fluorescent materials suitable for dye absorption.

## Introduction

Based on a global seafood consumption footprint study^1^, 154 million tonnes (*Mt*) of seafood were consumed in 2011 alone. With a global projected 7.2–12 million tons of fish waste discarded yearly^2,3^, fish scale waste is an abundant, sustainable natural resource that deserves more attention. The primitive fish scale has inspired the development of next-generation bionic flexible armour^4^, active camouflaging surfaces^5^, and transparent fish gelatin films for electroluminescence devices^6^. Its extracts have applications ranging from biomedical (wound healing^7^, bone^8^, and cornea repair^9^ etc.) to flexible electronics^6^, batteries^10^, and pollutants adsorption^11–14^. However, an extensive literature review shows limited studies on the optical properties (fluorescence, in particular) of primitive fish scales.

Fish scale primarily comprises collagen and hydroxyapatite (HAp). Collagen contains fluorescing chromophores with a reported short lifetime under UV excitation^4,5^, while HAp is intrinsically a wide bandgap inorganic material^6^. Development of these highly bio-compatible entities into fluorescence probes for in vivo studies involved efforts mainly focused on functionalizing HAp with dopants^15^ or breaking down fish scales to form carbon nanodots through mechanical and hydrothermal process combinations^7^. These processes require large amounts of time, energy and chemical resources. Thus, to reap the economic benefits of the low-cost waste material, the ability to achieve fluorescence enhancement of primitive fish scale with minimum intermediate steps is desired. With the recent discovery that human hair fluorescence can be enhanced through controlled thermal annealing^8^, the prospect of breaking down the collagen structure into its fluorescent variant while introducing defects into HAp through controlled annealing is a viable route toward achieving enhanced fluorescence in these primitive fish scales.

Biosorbents are emerging as green, cost-effective, and efficient alternatives to synthetic material for pollutant removal from wastewater^9^. The defective and denatured intrinsic structure of the fish scale biosorbent can result in a greater number of adsorption sites, thus enhancing the efficiency of this biosorbent. Of the many water pollutants, Rhodamine B (RhB) is of particular interest, not only because of the lack of research pertaining to the use of fish scale waste as a RhB biosorbent but also due to the highly toxic nature of RhB—cancer, respiratory diseases, liver failure and dermatitis are amongst the many maladies it can induce^10^, on top of the threat it poses to marine ecosystems due to its wide usage as a flow tracing agent^11^ and textile dyes^10^.

Inexpensive biomass such as activated carbon white sugar can remove 98% of RhB within 12 min of contact time^12^. However, the functionalization process is very energy- and time-intensive, involving multiple steps of chemical treatment, washing, and hours of high-temperature (700 °C) thermal annealing in a controlled environment. In comparison, a functionalized fish scale created via a single thermal annealing step at 270 °C in ambient for 3 min can effectively remove 91% of RhB within a contact time of 10 min. Hence, making this alternative material more energy-efficient, cost and time-saving. Considering that the average contact time for biomass removal of RhB (removal percentage: 85–95%) is 100 min^13,14,16–21^, the functionalized fish scale is shown to be ten times more efficient.

During this effort, we chanced upon another interesting application—using a functionalized fish scale as a medium in steganography, giving twists to old methods^22^. Many materials and data processes^23–25^ are utilized for steganography, allowing for certain advantages such as multiplexing steganography, ease of message transmission and multilevel security. However, many require more sophisticated instruments for message extraction and hack-prone computer code for deciphering messages^26^. Fish scale steganography is an alternative form that functions in a multidimensional level of hiding text and images at both micro and macroscopic levels. Information can easily be spread across different pieces that are compact enough to be transported. The widely available versatile laser writing techniques allow this discovery to be readily adapted and customized to the needs of its encoder. The potential of using natural products as a possible source of steganography material is a discovery on its own.

In this work, the concept of having materials with multiple functionalities and applicability is presented through the development of thermally treated fish scales as effective biosorbents for RhB removal and alternative steganographic material. Both applications are alternative uses for fish scale, and the functionalized fish scale can also effectively remove 91% of RhB within a contact time of 10 min. The emphasis is that the functionalized fish scale is a simple and economical sorbent to prepare. In a future of scarce resources, we believe such materials with multiple functionalities and applicability will be highly desirable.

## Results and discussion

### Macroscopic biofluorescence tuning of primitive fish scale

**Fig. 1:**
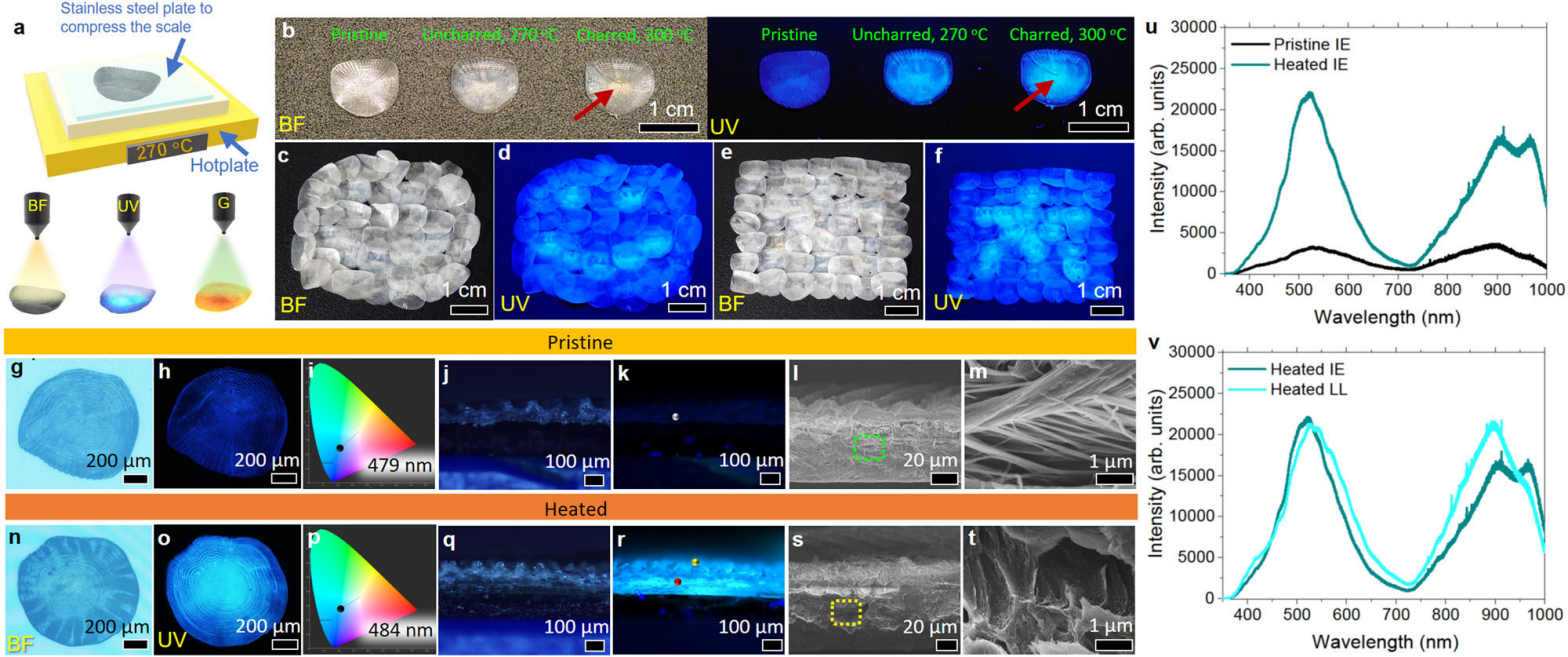
Fabrication and characterization of pristine versus heated fish scales. **a** Schematic of the thermal annealing process of the fish scale. The optical effect is achieved by comparing emissions from the heated fish scale under BF, UV, and G excitations. **b** Comparison of pristine, uncharred and charred fish scale under BF and UV excitation. **c**–**f** Hidden messages were created using an array of heated and pristine fish scales and revealed under UV illumination. **c**, **d** shows a smiley face, and **e**, **f** show a Chinese character symbolizing wood. **g**–**m** Pristine and **n**–**t** heated samples. **g**, **h**, **n**, **o** Top-view FM images of a single fish scale taken under BF and UV excitation. **i**, **p** Corresponding CIE1931 color profile of the fish scale under UV excitation. **j**, **k**, **q**, **r** Cross-section of the fish scale taken under BF and UV excitation. **l**, **m**, **s**, **t** Cross-section SEM images of the fish scale. **m**, **t** Higher magnification of the regions demarked in (**l**, **s**) by the green and yellow dotted boxes. **u**, **v** PL spectra from (**u**) IE of pristine and heated fish scale (indicated by the grey sphere in (**k**), and red sphere in (**r**). (**v**) IE and LL of heated fish scale (indicated by the red and yellow spheres in (**r**)).

Readily available freshwater *Red Tilapia* (RT) fish scales are heated to the required temperature on a hotplate for 3 min while sandwiched between two pre-heated stainless-steel plates. Optical characterizations are carried out under bright field (BF), ultraviolet (UV), and green (G) excitation light sources (Fig. 1a). Without heating, the pristine fish scale (Fig. 1b) exhibits faint fluorescence under UV excitation. After heating at 270 °C, intense cyan fluorescence is observed from uncharred heated fish scales under UV excitation. While brighter fluorescence is observed from charred fish scales with visible browning in BF (heated at 300 °C), their brittleness makes them unsuitable for the intended applications. Thus, this work will focus on uncharred heated fish scales. Under BF observation, the indiscernibility of heated fish scales from pristine fish scales allows discrete messages/patterns to be hidden among an assembly of fish scales and revealed under UV illumination. An assembly of fish scales under BF (Fig. 1c, e) unveils the hidden steganography as a smiley face (Fig. 1d) and a Chinese character symbolizing wood (Fig. 1f) under UV illumination.

Systematic heating is conducted by increasing the temperature from 230 to 280 °C. The most intense and uniform fluorescence enhancement occurs at 270 °C (Supplementary Fig. 1)—beyond this temperature, the fish scale’s charring reduces the available area on the fish scale that can fluoresce. Time-dependent heating at constant temperature (270 °C) was also conducted by heating the pristine fish scale for 1 to 4 min at 1-min intervals (Supplementary Fig. 2). Enhancement to the fish scale’s fluorescence without charring the fish scale was most intense for a heating duration of 3 min. At 4 min, charring of the fish scale resulted in a slight reduction in the fluorescence intensity, similar to Supplementary Fig. 1. Enhanced fluorescence is also observed on heated fish scales (270 °C, 3 min) from other species of fish, such as *Salmon* and *Yellow Tail* (Supplementary Fig. 3). However, the fluorescence enhancement lacks uniformity, which can be resolved by optimizing the heating temperature and duration for different fish scale types.

Generally, a piece of the fish scale (top view, Supplementary Fig. 4a) comprises different regions—the Anterior, the Lateral, the Focus, and the Posterior. Through cross-section imaging, the top layer (in contact with the fish’s living environment) is the limiting layer (LL). Below the LL is the elasmodine, which comprises discrete piles of unidirectionally aligned collagen fibers. This elasmodine is further divided into two layers: the external elasmodine (EE) and the internal elasmodine (IE). The subtle distinction between these two layers lies in the latter having lower mineral content (Supplementary Fig. 4b)^27^.

Figure 1g–t depicts the top and cross-section view of optical and SEM analyses of pristine (Fig. 1g–m) and heated (Fig. 1n–t) fish scales. Comparing Fig. 1g, h, n, o, the enhanced fluorescence from the heated fish scale is more concentrated at the Focus of the fish scale, which could be due to the natural convexity of the fish scale. The crest, which occurs at the Focus region, will exert pressure against the heating plates, resulting in greater heat transfer from the heated plates to the Focus region of the fish scale.

CIE1931 color profile of the fish scale under UV excitation shows a 5 nm redshift from 479 nm to 484 nm after heating (Fig. 1i, p), agreeing with the change in shade from blue to cyan from the heated fish scale. The cross-section FM image (Fig. 1j–k, q, r) highlights that no significant differences are observed between fluorescence from LL, EE and IE layers, which agrees with photoluminescence (PL) analyses of the fluorescence emitted when probed with a 325 nm laser beam (Fig. 1u, v). PL spectra show an enhancement of the peak intensity by a factor of 7 when compared between IE of pristine (grey dot in Fig. 1k) and heated fish scale (red dot in Fig. 1r). Likewise, the ratio of the area under the graph from heated IE over pristine IE is 3.9. In contrast to Fig. 1u, PL from the LL and IE (yellow and red dots in Fig. 1r) regions exhibit minimal differences (Fig. 1v).

### Characterization of fish scales, HAp, and collagen extracts

**Fig. 2:**
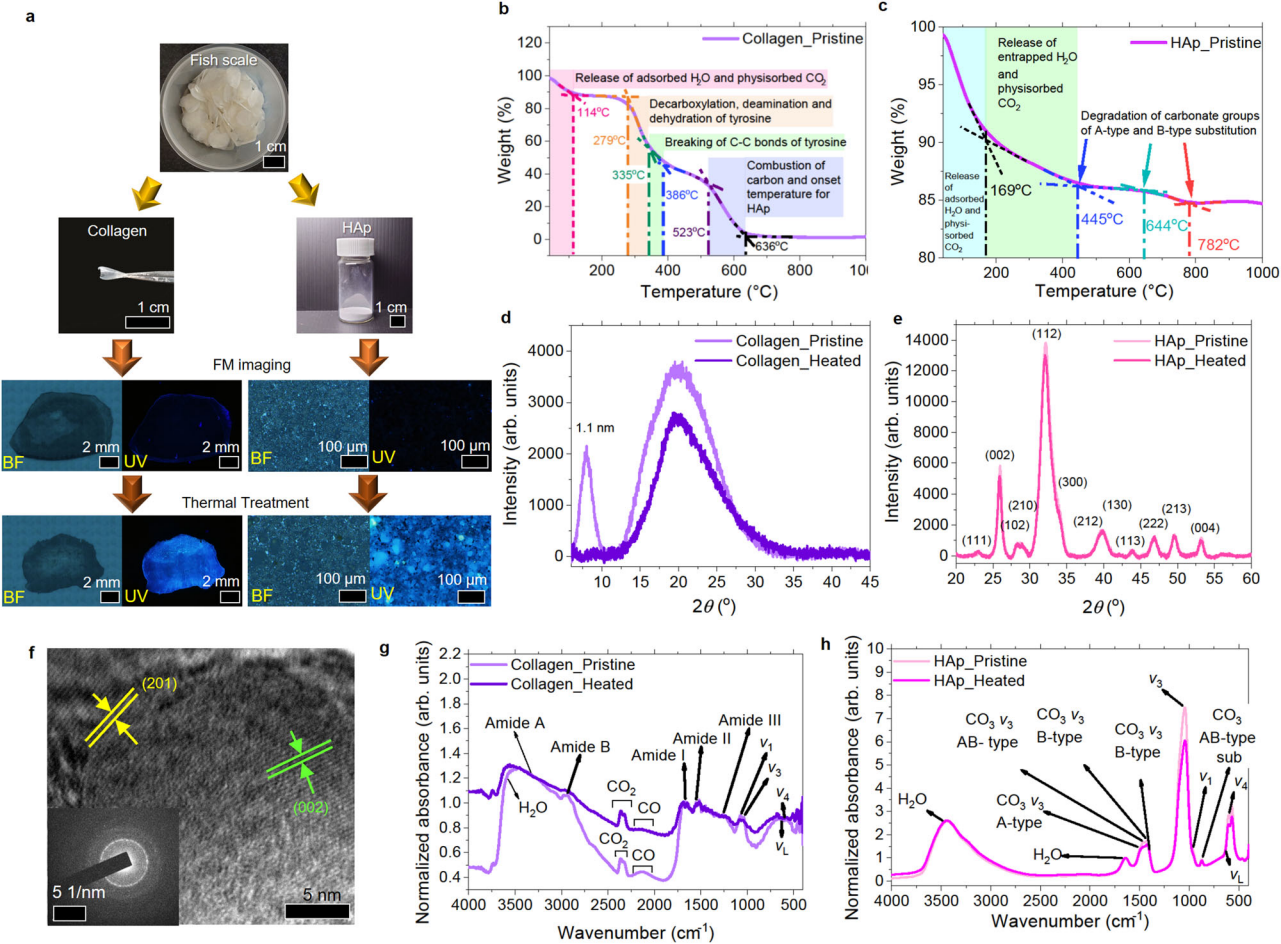
Characterization of pristine and heated collagen and HAp extracts. **a** Extraction of HAp from the primitive fish scale. Collagen and HAp are heated separately, and both materials individually exhibit significant fluorescence enhancement under UV excitation. **b**, **c** TG of pristine collagen and HAp extracts. **d**, **e** XRD analyses of pristine and heated collagen and HAp extracts. **f** HRTEM of extracted pristine HAp (inset corresponding SAED). **g**, **h** FTIR spectra of pristine and heated collagen and HAp extracts.

Fish scales primarily consist of 41–45% organic material (collagen, fat, etc.) and 38–46% inorganic material (calcium-deficient hydroxyapatite (HAp), calcium phosphate, etc.)^3^ with possible sources of environmentally induced defects/impurities^28^. HAp is isolated through de-calcification of fish scale^29^ to determine the underlying fluorescence mechanism. Pristine HAp and collagen show limited fluorescence. When heated at 270 °C for 3 min in ambient, both materials individually exhibited significant fluorescence enhancement under UV excitation (Fig. 2a). The possibility of a fluorescence contribution from NaCl formed during extraction is eliminated with the lack of fluorescence observed from pristine and heated NaCl samples (Supplementary Fig. 5).

Compositions and thermal behavior of the extracts are determined using thermogravimetric analysis (TGA), and the results are interpreted using a tangential baseline method^30^. The TG curve of the pristine primitive fish scale (Supplementary Fig. 6) shows predominately the release of adsorbed H_2_O, physisorbed CO_2_, and degradation of collagen and HAp, which agrees with TG curves from the separate entities of collagen and Hap (Fig. 2b, c). Pristine collagen extract shows the presence of both collagen and HAp (Fig. 2b). The mass loss at a temperature below 200 °C (~9 *wt* %) is attributed to the release of H_2_O and physisorbed CO_2_ molecules^31,32^. Mass variations between 279–335 °C (~23 *wt* %) and 335–386 °C (~12 *wt* %) are due to decarboxylation, deamination, dehydration of tyrosine (a fluorescence chromophore in collagen) and C–C bond breakage of tyrosine^33^. The last detected mass variation between 523 and 636 °C (~29 *wt* %) is attributed to carbon combustion and HAp degradation’s onset temperature.

TG of pristine HAp shows ~13 *wt* % loss when heated to 445 °C (Fig. 2c). Mass loss between 445 and 782 °C correlates to possible degradation of A-type and B-type carbonate substitution of OH^−^ and PO_4_^3−^ sites^34,35^, while the mass loss at a lower temperature is associated with the release of water and physisorbed gases^36^ such as CO_2_^34^. The presence of impurities such as CO_3_^2−^ and H_2_O within the mineralized tissues are attributed to physiological needs and are commonly found in biological HAp^34^. The results thus suggest that the extracted HAp is slightly carbonated. Derivative thermogravimetric (DTG) profiles of extracted collagen and HAp are presented in Supplementary Fig. 7 to better represent the changes in the *wt* % to temperature.

XRD analyses (Fig. 2d) of pristine collagen show a diffraction peak at 2*Ɵ* = 8.0^o^ corresponding to a 1.1 nm distance between the molecular chains^37,38^. The broad reflection peak (15−30°) corresponds to amorphous scattering due to unordered components of collagen fibers. Comparing pristine and heated collagen extract, the lack of diffraction peak at 2*Ɵ* = 8.0° and lower intensity of the broad reflection peak indicates increased disorder within the heated collagen structure. The narrower peak between 15 and 30° suggests a larger crystallite size within the heated collagen^37^. Both pristine and heated HAp (Fig. 2e) show similar diffraction profiles and peaks (JCPDS PDF: # 09-0432). However, lower intensities for peaks located at 2*Ɵ* = 25.9°, 40.0° after heating is detected. The FWHM for heated HAp is generally larger than pristine HAp (Supplementary Table 1), suggesting a loss of crystallinity in heated HAp following the release of trapped H_2_O and physisorbed CO_2_. The crystallinity of pristine HAp extract is evident in high-resolution TEM (Fig. 2f) and its selective area electron diffraction (inset Fig. 2f) image. Lattice spacings of 3.41 Å and 3.51 Å correspond to (002) and (201) lattice planes of the HAp hexagonal crystal structure, and distinct ring structures highlight the presence of polycrystallinity.

Detailed FTIR analyses of both isolated extracts (Fig. 2g, h) affirm the above observations. FTIR peaks between 2200 and 2400 cm^−1^ correspond to CO and CO_2_ (Fig. 2g)^38,39^. Peaks located at 3462 cm^−1^ and beyond are due to the physisorption of H_2_O molecules^40^. Distinct peaks from pristine collagen extract arising from N-H stretch in amide A and amide B^41^ are at 3315 cm^−1^ and 2940 cm^−1^. Peaks from amide I (C = O stretching), amide II (C-N stretching and N-H bending out of phase), and amide III (C-N stretching, O = C-N and N-H bending in-phase α-helix) are located at 1665 cm^−1^, 1551 cm^−1^, and 1260 cm^−1^ (see refs. ^42,43^). Aligned with the outcome of detecting HAp in collagen extract from the TG analyses, *v*_1_ (1074 cm^−1^), *v*_3_ (1039 cm^−1^) and *v*_4_ (592 cm^−1^) vibrational modes of (PO_4_^3−^) clusters, and *v*_L_ (629 cm^−1^), due to the liberational modes of OH in the hexagonal channels formed in the HAp structures^35,44^ are also detected in the FTIR spectrum of the collagen extract.

The FTIR of pristine HAp extracts (Fig. 2h) identifies typical modes of HAp samples while no amide-related peaks are detected. These include *v*_1_ (962 cm^−1^), *v*_3_ (1042 cm^−1^), and *v*_4_ (600 cm^−1^) vibrational modes of (PO_4_^3−^) clusters and *v*_L_ (635 cm^−1^) mode of OH. From the TG results, the extracted HAp is identified as a slightly carbonated form; this is confirmed by the identification of CO_3_-related vibrational modes: *v*_3_ vibration modes from AB-type CO_3_ substitution (871 cm^−1^, 1450 cm^−1^), CO_3_ groups substituting OH (A-type) at 1493 cm^−1^ and PO_4_ (B-type) groups at 1396 cm^−1^ and 1417 cm^−1^ (see refs. ^45–47^).

**Fig. 3:**
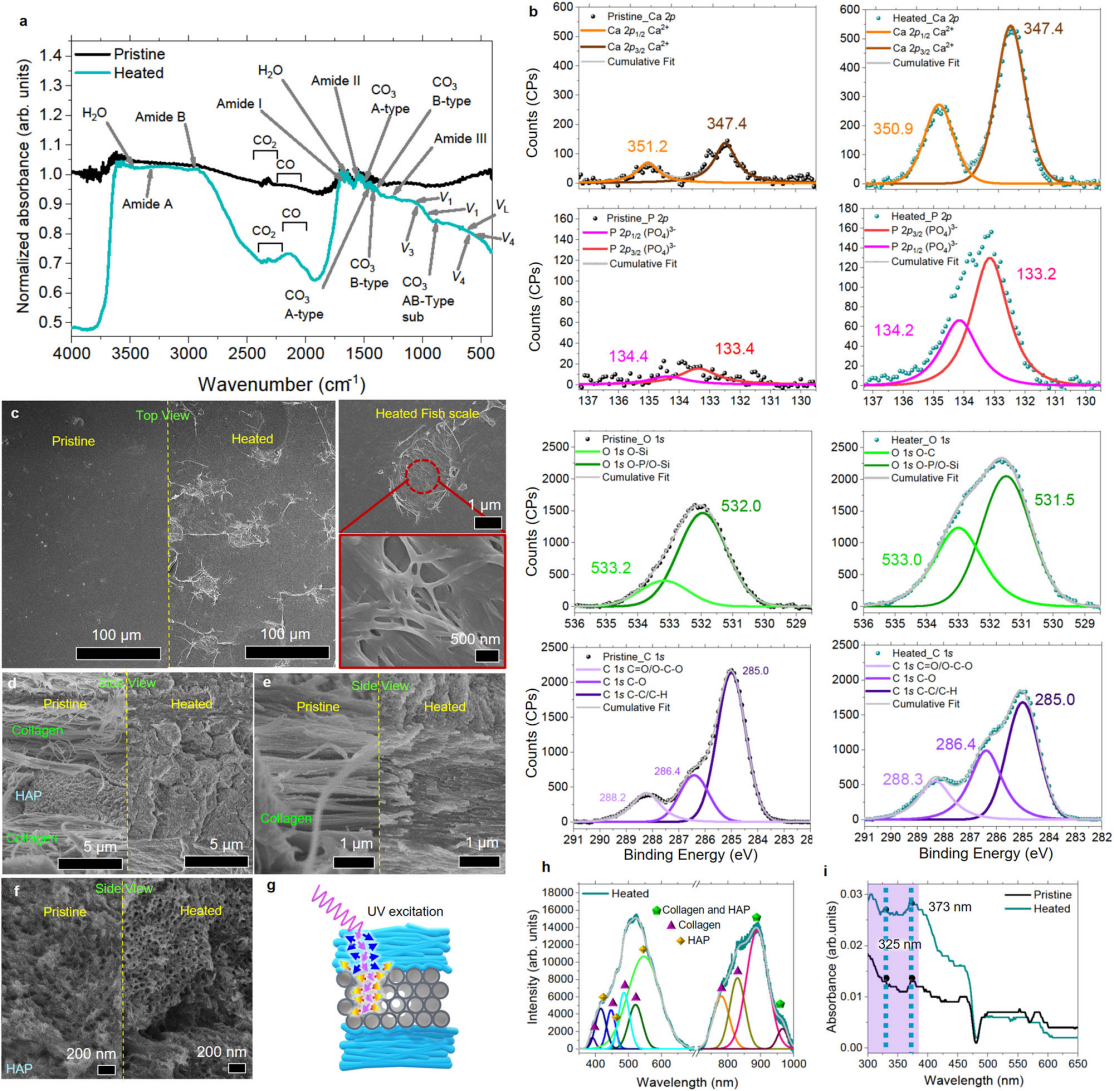
Characterization of pristine and heated fish scale. **a** FTIR of pristine and heated fish scale. **b** XPS spectra from pristine and heated fish scales. **c** Top and **d**–**f** side-view SEM images of the same piece (separated into two portions) of pristine and heated fish scale. **g** Schematic of the proposed cascading effect that fluorescence from denatured collagen structure can have at the interface to the adjacent HAp structure. **h** Deconvoluted PL spectra from primitive fish scale with the main contributors to the deconvoluted PL peaks as indicated. **i** UV–Vis absorption spectra of pristine and heated fish scale.

FTIR from heated collagen and HAp (Fig. 2g, h) exhibits a lower relative intensity to the C–C bond peak, suggesting that thermal annealing results in the decomposition of the collagen protein, the remaining HAp in the collagen extract and the HAp extract. The heated primitive fish scale (Fig. 3a) also shows a breakdown of collagen protein structure with the reduction in amide A, B, I, II, and III vibrational modes observed from extracted collagen. Degradation of HAp and A-, B-type CO_3_ substitutions are also detected through the reduction in the vibrational modes of (PO_4_^3−^) clusters, liberational modes of OH (*v*_L_) and A-, B-, AB-type CO_3_ substitutions.

Further characterization of the surface states of primitive heated fish scales is achieved through XPS analysis (Fig. 3b). C 1 *s* scan shows no significant shifts of C = O/O–C–O, C–O, and C–C/C–H related peaks from both samples. However, the heated sample shows an increase in the intensities of C = O/O–C–O and C–O components to that of the C–C/C–H component. Annealing in an oxygen-rich environment encourages bond formations between the broken C–C and C–H bonds. Collagen is also rich in oxygen, carbon, nitrogen, and hydrogen content. Its denaturation under thermal treatment can encourage O–P formation (attributed to the denaturation of HAp) and O–C (binding to carbonate ions). The fish’s living environment, which has an abundant Si source, contributed to O–Si contaminations in the pristine fish scale sample. Such bond formations are also reflected in the O 1 *s* scan, as more intense peaks for O–C and O–P are measured from the heated fish scale. XPS P 2*p* (PO_4_^3−^) and Ca 2*p* (Ca^2+^) scans also revealed stronger peaks after thermal annealing.

A comparison of the atomic % compositions of P (pristine: 0.6%) and Ca (pristine: 1.2%) shows a 4 and 1.8 times increase in P and Ca content from the heated fish scale, respectively. The increase in atomic % composition of P and Ca is attributed to the exposure of underlying collagen fiber and HAp structures due to damage sustained by the fish scale during the heating process (Fig. 3c). With heat-initiated phosphate substitution by carbonate ions from carbonate impurities within the primitive fish scale, diffusion of phosphate towards the surface could lead to a stronger signal being detected, thus accounting for the greater proportion of P compared to Ca.

### Proposed mechanism for thermally induced fluorescence

We propose that collagen and HAp contributed to the enhanced fluorescence observed. Due to the short lifetime of UV excited fluorescence chromophores cross-link in the collagen fibers^4,5^, presence of carbonated HAp whose broad PL spectrum closely matches our measured results^35,48^, will have a greater contribution to the observed fluorescence enhancement. As HAp and collagen are still distinguishable after thermal annealing (Fig. 3d–f), the interaction region between these organic and inorganic matrices likely occurs at the interfaces with the denatured collagen, forming a coating at the interface on the HAp.

When heated at 270 °C, pristine fish scales undergo structural decomposition, forming porous structures and exposing the underlying collagen fibers and HAp structures (Fig. 3f). The exposure facilitates the release of physisorbed CO_2_, H_2_O, and entrapped H_2_O molecules (Fig. 2b, c), forming cracks, increase in porosity (Fig. 3f), changes intercrystalline locations and encourages substitution of OH groups by intrinsic CO_3_ impurities. The structural changes also allow thermal dissipation to reach beyond the surface.

Collagen fluorescence has been attributed to multiple chromophores cross-links, such as tyrosine-like dityrosine and polytyrosine^49^. When thermally annealed, the process leads to collagen breakdown at the thermal labile domain, Gly877 to Pro941^50^, resulting in the random formation of tyrosine-like cross-links with longer, uncoupled coiled states confined by surrounding fibers network. Although tyrosine-like cross-links fluorescence mainly occurs between 400 and 420 nm^49^, dehydration results in the formation of polymer-in-a-box phenomenon^51^, such that the formation of elongated polymer chains can cause a redshift in the emission. While these chromophores under UV excitation have been reported to exhibit a short emission lifetime of <80 ns^5^, the collagen’s proximity to the porous carbonated HAp can result in secondary excitation from photons emitted by de-excited denatured collagen (Fig. 3g). The observable fusing of these collagen fibers upon thermal annealing also provided a less destructive optical path for excitation light transmission towards the porous HAp, where scattering and attenuation of light is minimized without traveling through multiple layers of air/collagen fiber interfaces. The process allows more photons to interact with the underlying carbonated HAp.

Although the energy gap of HAp was reported to be 5.23 eV^6^, since the heating temperature of 270 °C is much lower than the detected activating temperature (445 °C, Fig. 2c) required for the decomposition of A- and B-type carbonate substitutes, the presence of these carbonate impurities thus played a vital role in the observed fluorescence from pristine and heated HAp. When heated, distortions in the intrinsic HAp structure due to carbonate impurities and release of entrapped H_2_O molecules can result in the introduction of more energy levels within the forbidden zone that are responsible for electron-hole recombinations^35,52–54^, resulting in the fluorescence emission that is not observed from heated pure HAp structures (Supplementary Fig. 8). It is evident that energy levels are being introduced due to the presence of defects through the observation of multiple fluorescent wavelengths under different excitation lights (Supplementary Fig. 9).

The fluorescence profile of both heated collagen and HAP extracts are independently analyzed and matched to the PL obtained from the heated primitive fish scale. The result shows that both components contributed to the overall peaks’ formation in the heated fish scale (Fig. 3h) (refer to Supplementary Fig. 10 for a detailed discussion of PL analyses). Due to an increased number of fluorescence-activated species and sites within the heated primitive fish scale, fluorescence-contributing tyrosine-like cross-links and energy levels are formed within the forbidden zone of HAp. Stronger UV light absorbance is also detected from the heated fish scale (Fig. 3i). The stronger absorption then encourages the formation of more electron-hole pairs, contributing to the fluorescence enhancement upon recombination.

### Laser-assisted microscopic tuning of fish scale fluorescence

**Fig. 4:**
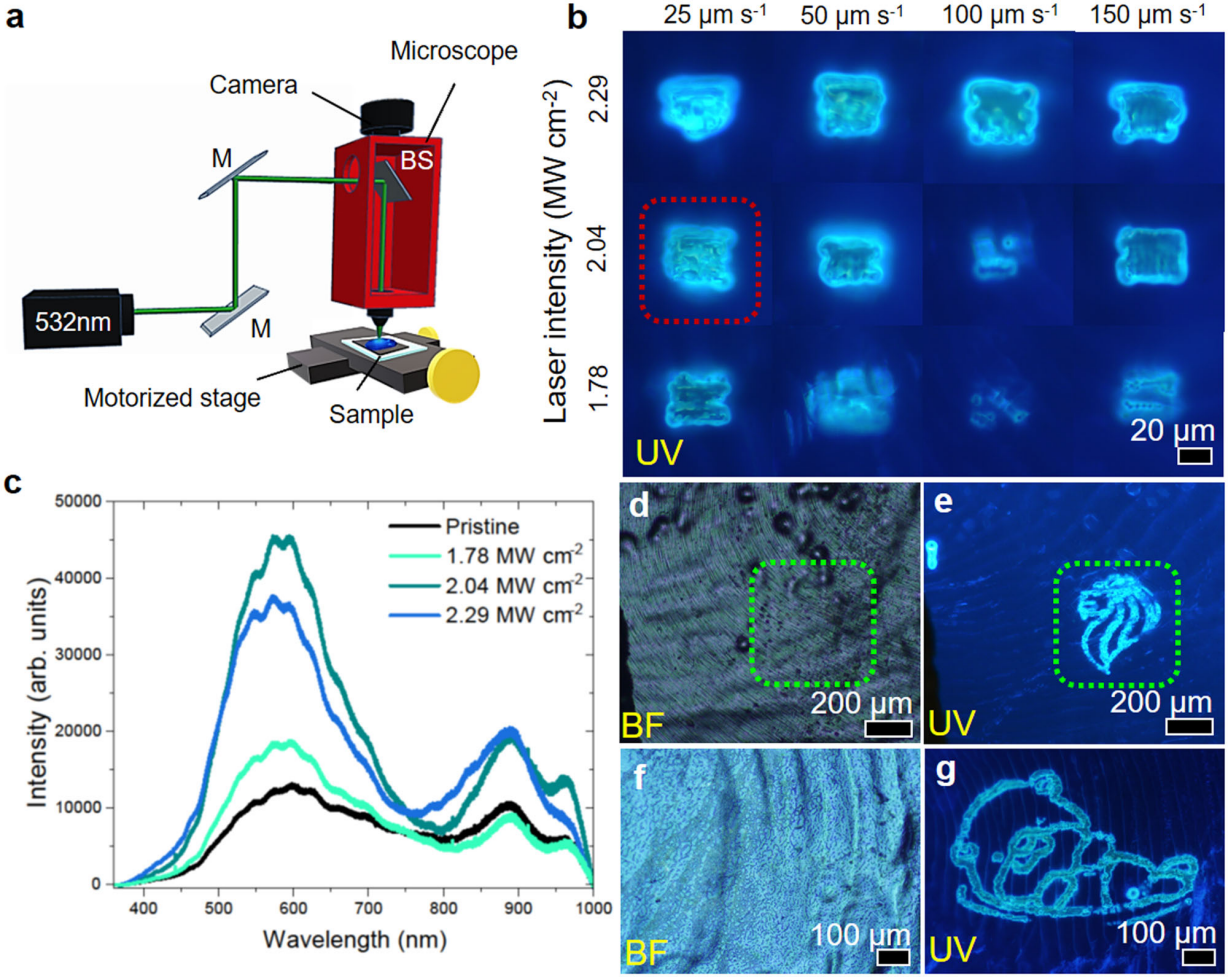
Laser-assisted microscopic tuning of fluorescence in primitive fish scale. **a** Schematic of the focused laser setup. **b** Systematic study on the effect of a focused laser beam as a sculpting tool to initiate fluorescence enhancement in fish scale by varying the laser intensity and patterning speed. **c** PL analysis of pristine and laser-treated (different laser intensity) fish scales at a constant speed of 25 µm s^−1^. **d**–**g** Laser-crafted steganos of **d**, **e** Merlion and **f**, **g** Otter structures.

A focused laser beam (Fig. 4a, detail in “Methods”) is used to achieve greater precision of localized heating and further develop fish scales for microscopic steganography and chemical detection. It has the capability and precision to impart a large amount of energy into a localized part of the sample over a short duration, resulting in photothermal changes to the material’s surface chemistry, morphology, and crystal structure. These changes can be controlled by varying the laser power and patterning speed. From Fig. 4b, the square created using 2.04 MW cm^−2^ at 25 µm s^−1^ patterning speed (enclosed in the red dotted box) exhibits the most uniform transformation.

Under BF, while the transformation appears to have left no discernable damage on the surface of the fish scale, slight changes to the morphology of the fish scale sample are observed when focused 30 µm below the surface of the fish scale (Supplementary Fig. 11). The approach allows engineered steganos that are hidden in plain sight to be placed on fish scales.

Figure 4c depicts PL spectra from the pristine sample versus regions treated with 1.78 MW cm^−2^, 2.04 MW cm^−2^, and 2.29 MW cm^−2^ of laser power at a fixed patterning speed of 25 µm s^−1^. The square treated with 2.04 MW cm^−2^ exhibits the most intense fluorescence, followed by the squares treated with 2.29 MW cm^−2^ and 1.78 MW cm^−2^, respectively. This outcome concurs with the observations seen in FM imaging. The versatility and controlled visibility of such localized enhanced fluorescence transformation on a fish scale is proven by an engraved fluorescing Merlion (Fig. 4d, e) and Otter (Fig. 4f, g) pattern on the fish scale. Under BF examination, no distinct markings are observed on the sites of the laser-patterned regions. However, once the excitation source changes to UV, the Merlion and Otter patterns emerge while emitting a vivid cyan fluorescence. The laser-initiated fluorescence enhancement in fish scale exhibits good stability in ambient conditions even after 5 months of creation (Supplementary Fig. 12).

**Fig. 5:**
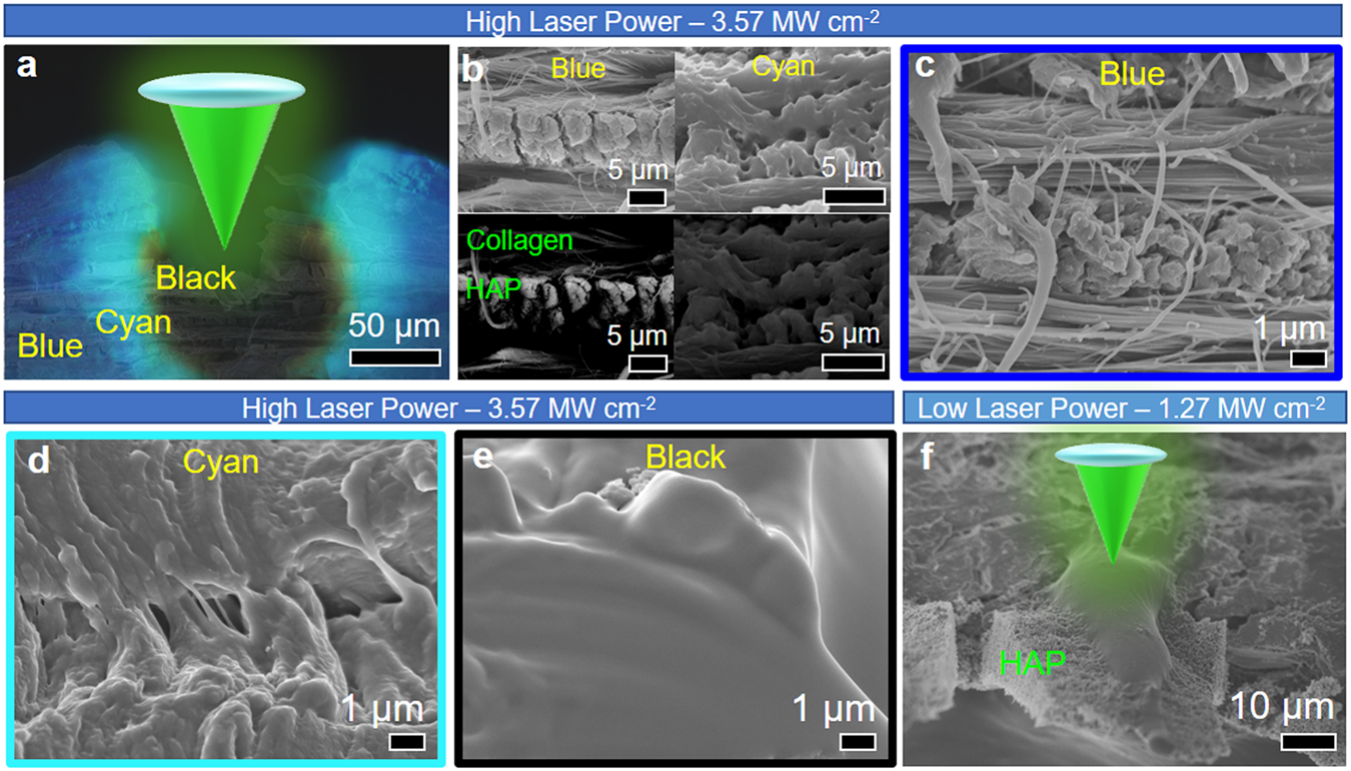
SEM characterization of laser-treated fish scale. **a** Overlayed cross-sectional FM image of high power density (3.57 MW cm^-2^) laser-treated fish scale under UV excitation on the corresponding cross-section SEM image. **b** Higher magnification of the section shows blue biofluorescence from the pristine sample and cyan biofluorescence from the laser-treated sample. Bottom row: BEI images. **c**–**e** Higher magnification of blue, cyan and black regions as indicated in (**a**). **f** Effect of low power density laser (1.27 MW cm^−2^) treatment of exposed HAP along the cross-section of a fish scale.

The locality and ease of altering the amount of heat energy deposited by the focused laser beam on the fish scale allow for verification of the proposed mechanism. The fish scale is intentionally patterned with a laser of high power density (3.57 MW cm^−2^). The laser-treated region’s cross-section FM image overlaps with the corresponding SEM image of the fish scale (Fig. 5a) to identify the surface morphology of the specific regions emitting different fluorescence.

The focused laser’s Gaussian beam profile highlights the existence of a particular heat zone that facilitates the formation of fluorescing active species and carbonate impurities (cyan fluorescence). Far from the laser spot, the pristine section (blue fluorescence) of both SEM and BEI images (Fig. 5b, c) show alternating multi-layered collagen fibers and HAp crystals^3,55^. Within the cyan fluorescing zone, collagen and HAp denatured (Fig. 5b, d). In the region closest to the laser beam (Fig. 5e), the lack of fluorescence is attributed to the rapid, intense heating from the laser, which readily breakdowns molecular bonds and decomposes impurities such as carbonate substitutes and entrapped H_2_O molecules within both Gly-X-Y and HAp structures. Subsequent quick removal of the laser from the annealing spot leads to fast cooling, leaving the laser-treated region in a quenched state with minimal molecular restructuring.

Using a lower laser power density of 1.27 MW cm^−2^ to anneal a portion of the HAp crystal layer protruding from the fish scale (Fig. 5f), HAp crystals along the laser path undergo a structural change. This likely arises from the release of entrapped H_2_O molecules and results in the formation of substitutional bonds between the intrinsic structure and carbonate impurities. More heat is imparted by reducing the speed of the laser process, causing the formation of larger areas of denaturation, thus presenting a means for controlled fluorescence (Supplementary Fig. 13).

### Applications

Collagen fibers-HAp crystal complexes are established adsorbents for heavy metals^29,56^ and dyes^57–59^. However, no report has been published on implementing fish scales as an adsorbent for RhB dye. RhB is an amphoteric dye with a pink appearance under white light and fluoresces bright pink and orange under UV and G excitation at high concentrations. It is used in flow tracing due to its fluorescence. However, its toxicity, owing to its ability to cause oxidative stress on cells^11^ when ingested, emphasized the need for the chemical cleaning of water used. With the extraction processes being both time-consuming and involving a fair amount of chemical resources, it will be more economical if fish scales can be implemented as an adsorbent with a minimum treatment process.

**Fig. 6:**
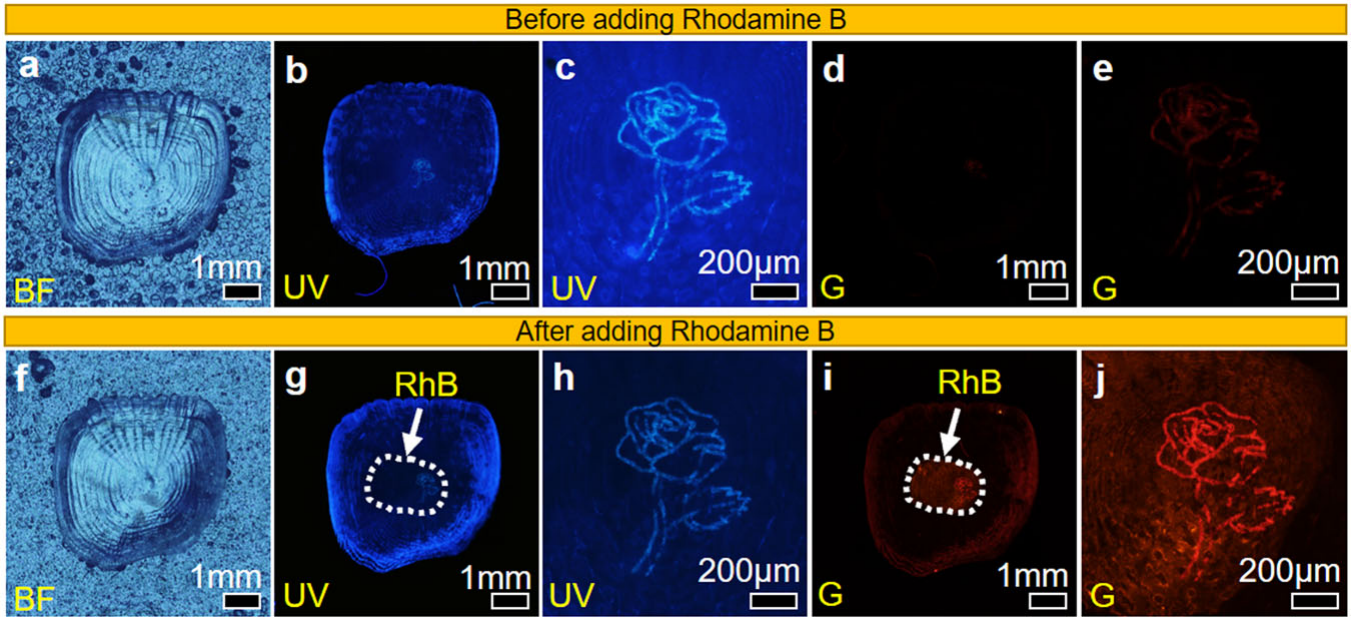
Enhanced adsorption of RhB on laser-modified fish scale. **a** No observable changes to the fish scale after a Rose design is patterned on the fish scale. **b**, **c** Cyan and **d**, **e** dull orange fluorescence observed from the laser pattern site under UV and G excitation. **f**–**j** After the addition of RhB, **f**–**h** the laser-patterned fluorescence appears slightly dimmer under UV excitation. **i**, **j** More intense orange fluorescence is observed from the Rose pattern after interacting with RhB. The white dotted circle shows the region that interacts with the RhB solution for 3 min before rinsing off with DI water.

A Rose pattern crafted at the center of a single primitive fish scale with a focused laser beam appears invisible under BF while exhibiting cyan and dull orange fluorescence under UV and G (Fig. 6a–e). A droplet of 10^−8 ^m of RhB is placed on the fish scale for 3 min, after which the scale is rinsed with DI water and blown dry with N_2_ gas. At this concentration, the solution emits very dim fluorescent under UV, and a very slightly orange fluorescent is observed under G excitation (Supplementary Fig. 14). No significant visual difference between the laser-patterned Rose and the surrounding pristine sample is observed under BF (Fig. 6f). Under UV excitation (Fig. 6g, h) the Rose pattern appears dimmer, due to the presence of RhB molecules, not readily excited by UV light at this concentration. The region of the fish scale interacting with the RhB solution is enclosed within the white dotted circle (Fig. 6g, i). It emits a weak orange fluorescence under G excitation (Fig. 6i). The laser-patterned Rose exhibits a more intense orange emission within the same region, more substantial than the surrounding pristine sample under G excitation (Fig. 6i, j), thus proving the feasibility of developing a sensitive microdetector from readily accessible fish waste.

### Biosorbancy of heated fish scales on RhB

All subsequent PL spectra are obtained under 532 nm excitation. A 4.00 µL droplet of 10^−6^ m of RhB is placed onto fish scales heated at 260 °C, 270 °C, and 280 °C for 3 min before the scales are rinsed in DI water and blown dry with N_2_ gas. Supplementary Fig. 15 depicts FM images under G excitation (Supplementary Fig. 15a–c) and PL (Supplementary Fig. 15d) spectra obtained from the regions that interacted with RhB (the orange dot in Fig. 15a–c). Based on the intensity of orange fluorescence from the RhB-treated regions, the qualitative and quantitative results highlight that fish scales heated at 270 °C are most effective as an adsorbent of RhB.

**Fig. 7:**
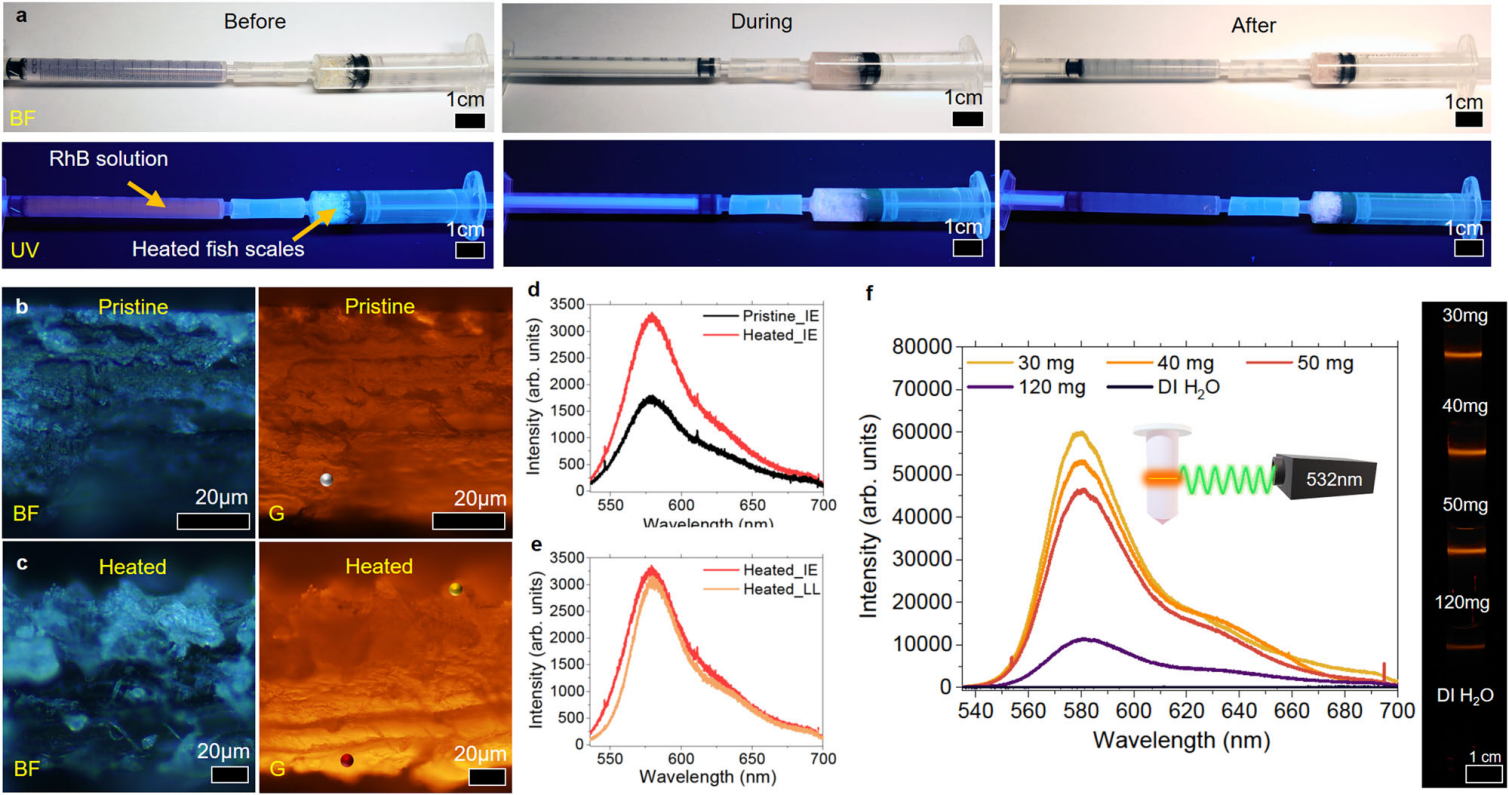
Heated fish scale as biosorbent for RhB. **a** Syringe setup was used to test the adsorption capability of the fish scale for RhB removal. **b**, **c** Cross-sectional view of pristine and heated fish scale after submerging in RhB under BF and green excitation. **d**, **e** PL spectra were taken at the IE ((**b**, **c**): red and grey dots) and LL (**c**): yellow dot) of heated and pristine fish scale with RhB. **f** PL spectra of fish scale-treated RhB-contaminated water. The inset shows a 532 nm laser setup that excites the fish scale-treated RhB solution. The setup achieves the column of optical images taken of the RhB-contaminated water after being treated with different amounts of fish scales. All PL spectra in this figure are taken using a 532 nm monochromatic laser.

A customized setup comprising two syringes connected by a rubber tubbing is designed to test the adsorptive capabilities of pristine and heated fish scales (Fig. 7a). Images are captured under BF and UV illumination. One of the syringes is filled with 10^−6^ m of RhB (pink), while the other is filled with fish scales cut into smaller pieces (before). The solution is transferred from one syringe to the other, soaking the fish scales for 10 min (during) before the treated solution is extracted back to the syringe (After), leaving a pink appearance on the fish scales from the adsorption of RhB. Minimal fluorescence observed from the treated solution ascertains the successful removal of RhB by the heated fish scales.

Cross-sectional FM images of pristine and heated fish scales after being immersed in RhB are depicted in Fig. 7b, c. Figure 7d, e illustrates the PL spectra taken at the IE (red and grey dots) and LL (yellow dot) layers of the heated and pristine fish scale with RhB. Both qualitative and quantitative analyses indicate stronger fluorescence detected from heated fish scales (two fold enhancement in PL intensity, Fig. 7d), with a minimal distinction between IE and LL regions of the heated fish scale (Fig. 7e). With heated fish scales proven as a more effective adsorbant of RhB, subsequent studies will be conducted using heated fish scales.

Heated fish scales are cut into smaller pieces with an average width of ~2 mm and separated into portions of 30 mg, 40 mg, 50 mg, and 120 mg before submerging into 4 bottles of 2 ml of 10^−6^ m of RhB for 10 min. The heated fish scales are subsequently removed, and the remaining solutions are characterized. Figure 7f presents PL spectra and optical images of these solutions under 532 nm laser excitation. The most significant reduction in the intensity of fluorescence detected at 579.9 nm occurs with 120 mg of cut-heated fish scales, which resulted in a fourfold reduction from 50 mg of heated fish scales. With 120 mg of these heated fish scale fish scales, 91% of RhB is effectively removed from 2 ml of 10^−6^ m of RhB within 10 min. The loss of intensity is also reflected in the optical image (Fig. 7f) captured by sending a 532 nm laser through the treated solution. Fluorescence from the RhB dye readily reveals the laser path, showing an orange line through the solution. The illuminated path gets dimmer as the concentration of RhB reduces.

### Proposed adsorption mechanism

**Fig. 8:**
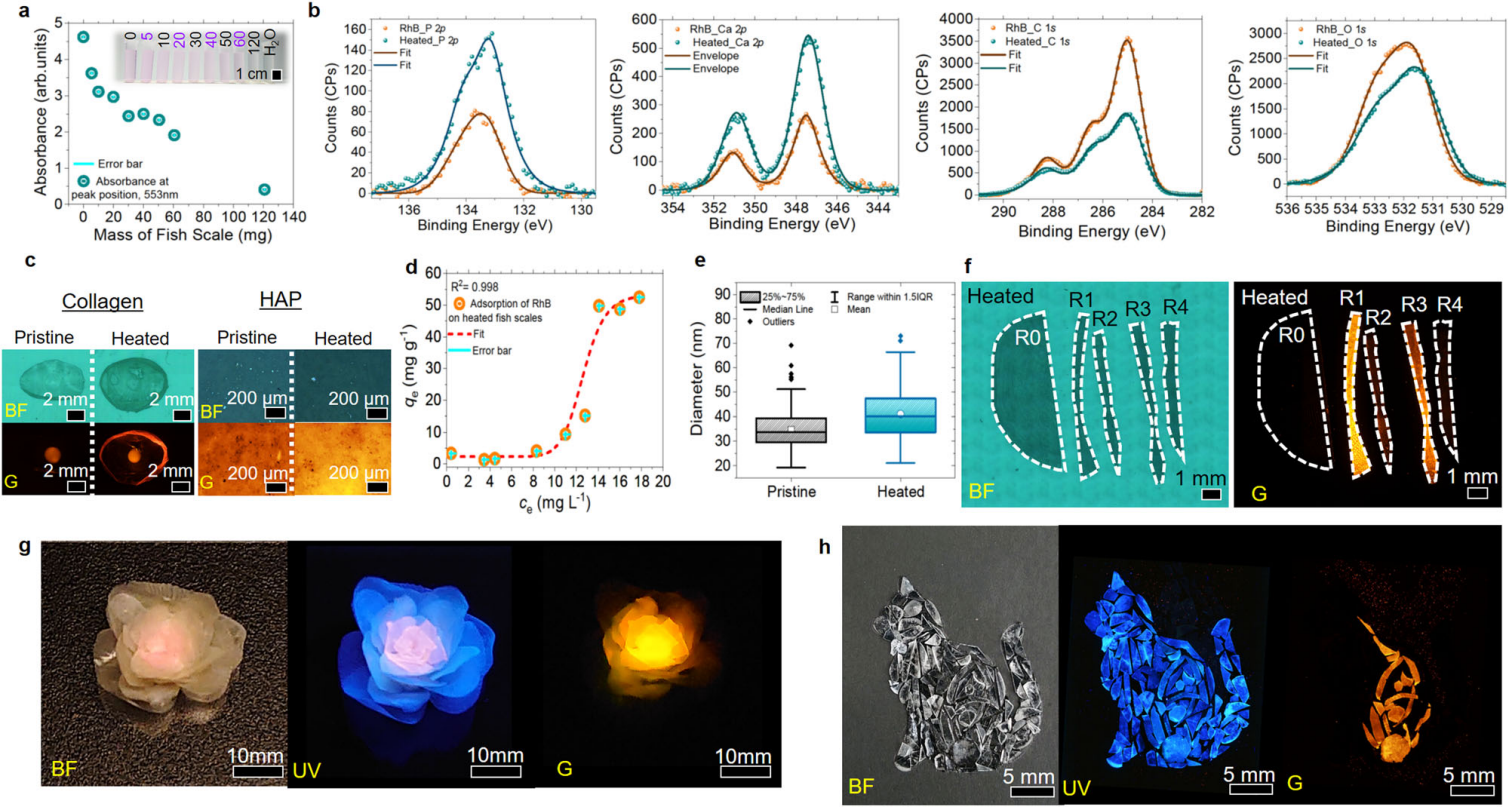
Proposed adsorption mechanism, recoverability, and steganography application. **a** A plot of UV–Vis absorbance intensity at 553 nm to the mass of the fish scales used. **b** XPS analyses of heated fish scales and those treated with RhB. **c** FM images of pristine and heated collagen and HAP extracts after interacting with RhB molecules. **d** RhB biosorption isotherms on heated fish scales. **e** Distributions of pore size of pristine and heated fish scales. **f** Recovery of heated fish scale (R0) from RhB adsorption through multiple rounds of adsorption (R1, R3) and desorption (R2, R4) via ultra-sonication in DI water. **g** Macroscopic 3D flower art piece created from the pristine, heated, and heated fish scale that has been used to adsorb RhB from water taken under BF, UV, and G excitation. **h** Sculpture of a cat made from heated fish scales under BF and UV illumination. Under green excitation, a hidden waveparticle sculpture is revealed within the cat sculpt.

UV–Vis absorbance scan is conducted using the heated fish scale (smaller intervals weight distribution) treated RhB solution. A plot of the intensity of the absorption peak at 553 nm to the mass of the heated fish scale shows a positive correlation between the mass and the efficacy of adsorption of RhB by the fish scales. Inset shows an optical image of RhB solution treated with different amounts of heated fish scales (Fig. 8a). Such observations show succinctly that simple thermal treatment can engineer and enrich the physical properties of the fish scale, further bringing out its potential in pollutant removal.

XPS scans (Fig. 8b) conducted on a single heated fish scale that was separated into two pieces, with one of the pieces submerged in 10^−6^ m of RhB for 10 min, resulted in a drastic reduction in PO_4_^3−^ (atomic % of 3.0 to 1.0) and Ca^2+^ (atomic % of 3.4 to 1.1) detected as the RhB molecules adsorbed onto the exposed HAp structures from the heated fish scale. The presence of C–O (atomic % of 18.8 to 17.5), C = O/O–C–O (atomic % of 10.8 to 10.2), O–P/O–Si (atomic % of 16.2 to 13.0), and O–C (atomic % of 10.4 to 9.0) also experienced a slight reduction. Concurrently, more C–C/C–H bonds (atomic % of 28.8 to 39.0) are detected after interaction with RhB. As an amphoteric dye, RhB contains positive and negative charges, suggesting they can readily interact with PO_4_^3−^, Ca^2+^, and oxide-related bonds. The intrinsic nature of the RhB molecules also allows it to contribute to forming more C–C/C–H bonds.

The above proposal is verified by investigating extracted collagen and HAp towards the adsorption of RhB (Fig. 8c). The heated form of both extracts exhibits stronger fluorescence attributed to more RhB molecules than pristine extracts. Sorption isotherm analysis of RhB onto heated fish scales (Fig. 8d) agrees with the adaption of the Dubinin-Astakov model proposed by Blázquez et al.^60^, which suggests that the adsorption process of RhB on heated fish scales involved multimolecular layer formation in mesopores (diameter: 2–50 nm) with both strong and weak adsorbate-adsorbent interactions. The distribution plot of pores diameter (Fig. 8e) from pristine and heated fish scales (Fig. 3f) shows that the average pores’ size falls within the proposed model’s range.

Heat-induced larger pores (increase in pore size by 6 nm) also facilitates diffusions of RhB solution into the fish scale structure, encouraging greater uptake of RhB molecules from the solution. Interactions between RhB and heated fish scale are investigated by sending the same piece of RhB adsorbed heated fish scale through multiple rounds of adsorption (R1, R3 in Fig. 8f) and sonication in DI water (R2, R4 in Fig. 8f). Detailed in the method section. With weak fluorescent detected from R2 and R4 heated fish scales, the outcome agrees with the above model that the adsorption of RhB onto heated fish scale involve both strong and weak interactions. Similar processes and results are also achieved when the heated fish scales adsorb methylene blue (MB) molecules from DI water. 120 mg of heated fish scales are determined to be capable of removing 64% of 10^−8^ m MB within 3 min (Supplementary Fig. 16). Both results from RhB and MB adsorption prove the recoverability of the heated fish scale, thus paving the way for implementing the heated fish scale as an efficient, sustainable alternative biosorbent.

Control over the adsorption of RhB on heated fish scales while utilizing different colors and strengths of fluorescence from pristine, heated, and RhB-treated fish scales allows cipher encoding into paintings and sculptures. Arrangement of pristine (edge petals), heated (middle petals), and RhB adsorbed (core petals) fish scales enable the creation of a 3D macroscopic flower art piece with fluorescence emission capability (Fig. 8g). By reducing the concentration of RhB to 10^−7^ m, adsorption of RhB onto the heated fish scale can occur without the fish scale exhibiting any visible changes under white light and UV illumination. Figure 8h shows a cat sculpture created from a blend of heated fish scales and heated fish scales that had been immersed in RhB. No clear differences under BF or UV excitation are observed from the sculpture. Switching the illuminating source to green light revealed an orange fluorescing waveparticle sculpture encoded using heated fish scales immersed in RhB, thus creating art with steganography.

In summary, we propose using controlled annealing in the ambient to transform the fluorescence property of primitive fish scales. Denaturation of collagen resulted in the random formation of tyrosine-like cross-links with longer, uncoupled coiled states within the vicinity of carbonated defective HAp, contributing to the enhanced fluorescence of fish scale waste detected. These strong fluorescing fish scales effectively remove 91% of RhB pollutants within 10 min, which is ten times faster when compared to other biomass with RhB pollutants removal percentage of 85–95% and can serve as natural steganographic material with multidimensional security capable of micro- and macroscopic text and imagery transmission. Thus, using a single processing step, fish scale waste is established as an economical alternative material with multidimensional application. Looking forward, we believe that uncovering alternative properties of natural waste materials and re-inventing them as a host of multiple applications is a viable route towards a sustainable and resource-constrained future.

## Methods

### Treatment of the collected *red tilapia* fish scales

*Red Tilapia* fish scales are collected from the local supermarket. The scales are washed in detergent before rinsing in tap water. The cleaned scales are then air-dried and stored in a sealed container at room temperature for further use.

### Sex and gender equity in research

The research does not involve the use of live or dead fish. We only purchase fish scales from the local supermarket. We do not select the fish scales base on the sex of the fish as we are looking at the general behavior of fish scales regardless of the sex of the fish from which it is obtained from. This study does not include any human research participants.

### Macroscopic thermal annealing of *red tilapia* fish scales

Fish scales are sandwiched between two pre-heated 8.9-cm × 8.9-cm stainless-steel plates (each weighing 185 g) and placed on a hotplate (Thermo Fisher Cimarec^+^). The process caters for large-scale production while ensuring even heating and maintaining the shape of the fish scales. The heating process occurs under ambient conditions at various temperatures for specific durations and temperatures listed in this report.

### Focused laser micro-patterning of *red tilapia* fish scales

A primitive fish scale, with IE facing the microscope’s lens, is secured onto a silicon wafer with double-sided carbon tape for the laser patterning. The underlying carbon tape served as an adsorption layer for the focused laser beam to rapidly increase the temperature within the highly transparent fish scale. Thus, the process enabled laser-initiated microscopic changes to the IE layer and enhanced the observed fluorescence from the laser-treated fish scale. On the computer-controlled motorized stage, the fish scale moves relative to a focused beam of 532 nm laser that passes through a ×100 lens (final beam diameter: 1 µm). In all, 50 µm × 50 µm laser-patterned squares (at different laser power and patterning speeds) are then created on the fish scale.

### Extraction of hydroxyapatite

In total, 50 mL of 0.8 m HCL is added to 2.50 g of fish scales. The mixture is then sonicated at 60 °C for 2 h at 0.4 kW. In all, 30 mL of supernatant is extracted and adjusted to pH 12 with 5 m NaOH. The pH-adjusted supernatant is sonicated at 0.4 kW at 22 °C for 45 min and centrifuged at 22,140×*g* for 45 min. The separated solution is removed, and DI water is added for rinsing. The rinsing process (centrifuge coupled with the removal and addition of DI water) is repeated three times to ensure that the supernatant is clean before placing it into an oven for drying at 60 °C for 24 h. The process resulted in the extraction of 0.43 g of HAP. Pure HAP is obtained from Merck (900194-25 G). Extracted quantities are not sufficient for full characterization.

### Recovery of heated fish scale from RhB and MB treatment

To recover the heated fish scale from RhB adsorption, the heated fish scale is divided into two pieces, and one is kept as R0. The remaining piece is placed into 10^−6 ^m of RhB solution for 10 min. A thin sample piece is removed (R1) from the RhB adsorbed samples, and the remaining larger piece is sent for sonication. The first round of the sonication process takes place in DI water at 22 °C for 30 min at 0.4 kW. After the sonication process, another thin piece is removed (R2) and the adsorption (R3) – desorption (R4) process is repeated for a second round. All five pieces of samples are then placed between two glass slides for imaging. A similar process is repeated for the MB adsorption-desorption study, with 10^−8 ^m of MB used, and the adsorption process takes 3 min for each round.

### X-ray photoelectron spectroscopy (XPS)

XPS analysis uses Thermo Fisher Scientific Theta Probe XPS with monochromatic Al K alpha X-ray (1486.7 eV). Charge correction for binding energy was based on C1s from adventitious carbon at 285.0 eV.

### Fluorescence microscope (FM)

FM (Olympus BX51) with filter cubes U-MWU2 (330–385 nm), U-MWB2 (450–500 nm), U-MWG2 (510–550 nm), and U-MWIY2 (530–580 nm) are used to illuminate the samples with ultraviolet, blue, green and yellow light. Olympus DP74 camera and objective lens MPlanFLN 5x, LMPlanFLN 10×, LMPlanFLN 20×, LMPlanFLN 50×, and MPlanFLN 100× are used for imaging. CIE1931 color profiles are collected using the Ocean Optics Flame spectrometer attached to the FM.

### Further characterizations

Raman and photoluminescence (PL) are carried out using a Renishaw Micro Raman System (Renishaw inVia Qontor) coupled with a 532 nm and 325 nm laser. Fourier-transform infrared (FTIR) analysis is measured using IRPrestige-21. FTIR graphs are smoothed by taking adjacent-average and normalized to the C–C peak (1645 cm^−1^). TGA and DTG analyses use the TA instruments Model: Discovery TGA. SEM images are taken using (JEOL JSM6700-F); TEM images are taken using (JEOL 2010-F), and the absorbance spectra are collected using Ultraviolet-visible spectroscopy (UV–VIS, Shimadzu UV−2600 UV–Vis Spectrophotometer). X-ray diffraction (XRD) data are collected using Bruker D8 Advance Powder XRD with Cu K alpha radiation.

### Supplementary information

Supplementary Information

Peer Review File

### Source data

Source Data

## Data Availability

The source data generated in this study have been deposited in the Figshare database under the accession code 10.6084/m9.figshare.22647988. Source data are provided with this paper.
