# Author Correction: Upcycling fish scales through heating for steganography and Rhodamine B adsorption application

**DOI:** 10.1038/s41467-023-43515-5

**Published:** 2023-11-24

**Authors:** Malcolm Miao Geng Sow, Zheng Zhang, Chorng Haur Sow, Sharon Xiaodai Lim

**Affiliations:** 1 NUS High School of Mathematics and Science, 20 Clementi Avenue 1, Singapore, 129957 Singapore; 2 Institute of Materials Research and Engineering (IMRE), Agency for Science, Technology and Research (A*STAR), 2 Fusionopolis Way, Innovis #08-03, Singapore, 138634 Singapore; 3 Department of Physics, National University of Singapore, 2 Science Drive 3, Singapore, 117542 Singapore

**Keywords:** Synthesis and processing, Optical materials, Biopolymers

Correction to: *Nature Communications* 10.1038/s41467-023-42080-1, published online 16 October 2023

An incorrect version of the Source Data file was originally published with this article; it has now been replaced with the correct one.

The original article has been corrected.

### Supplementary information

Updated Source Data

