# Supplementary material for: Upcycling fish scales through heating for steganography and Rhodamine B adsorption application
Source: Nat Commun. 2023 Oct 16;14:6508. doi: 10.1038/s41467-023-42080-1 (PMC10579236; doi:10.1038/s41467-023-42080-1)
Supplement: Supplementary file 1 — Supplementary Information [file 41467_2023_42080_MOESM1_ESM.pdf]

nature portfolio

Peer Review File

Upcycling Fish Scales Through Heating for Steganography and Rhodamine B Adsorption Application

Open Access This file is licensed under a Creative Commons Attribution 4.0 International License, which permits use, sharing, adaptation, distribution and reproduction in any medium or format, as long as you give appropriate credit to the original author(s) and the source, provide a link to the Creative Commons license, and indicate if changes were made. In the cases where the authors are anonymous, such as is the case for the reports of anonymous peer reviewers, author attribution should be to 'Anonymous Referee' followed by a clear attribution to the source work. The images or other third party material in this file are included in the article's Creative Commons license, unless indicated otherwise in a credit line to the material. If material is not included in the article's Creative Commons license and your intended use is not permitted by statutory regulation or exceeds the permitted use, you will need to obtain permission directly from the copyright holder. To view a copy of this license, visit http://creativecommons.org/licenses/by/4.0/.

REVIEWER COMMENTS

Reviewer #1 (Remarks to the Author):

In this work, the authors employed abundant and eco-friendly fish scales for the subtle fabrication of bifunctional materials via facile heating treatment for steganography and RhB adsorption applications. This work is interesting and a good storyline is presented in the main text. The majority of the results could be supported by the corresponding experiments. Moreover, the topic of this work is within the scope of the journal. Therefore, this manuscript is recommended to the journal after a Major Revision. The comments are as follows:
1. What chemical structures or moieties endow the heated fish scales with bright fluorescence emission? Please describe it in detail.
2. In Figure 2c and lines 282-284， the authors mentioned that "….shows 3.6 times and 1.8 times increase in phosphorous and calcium content from…" Why the contents of P and Ca greatly increased after heating treatment? Is the charring process of the fish scale? If it is, why the content of C decrease compared to the pristine one? Please give some comments on it.
3. How long this enhanced fluorescence property could be maintained after heating treatment?
4. The authors mentioned the effect of temperature on the fluorescence property of the fish scale. However, no related mechanism was described in the main text.
5. Line 200-202, the authors mentioned that "the most intense and uniform fluorescence enhancement occurs at 270 oC". However, in the Figure b, the intensity of fluorescence of heated fish scales at 300 oC seems higher than that of at 270 oC. Why?
6. Lin 300, "collagen shows a 5-fold and 10-fold increase in PL for both ….." Please explain this result. Meanwhile, Line 312, the authors mentioned that "collagen has a greater contribution to the observed fluorescence enhancement" Any references or experiments could support this conclusion?
7. The adsorption capacity and behavior of the heated fish scales toward RhB is suggested to investigate in detail including kinetics, isotherm and effect factors et al.
8. The mechanism of the heated fish scales toward RhB should also be a proposal, although the authors explored the adsorption behavior of the heated fish scales through XPS test.
9. How about the adsorption capacity of the heated fish scales toward other organic dyes? Please check it.
10. How to achieve the desorption of the RhB-adsorbed fish scales? How about the recyclability of this biosorbent?

Reviewer #2 (Remarks to the Author):

In this paper, the authors investigated the enhanced fluorescence and RhB adsorbent properties of the heated fish scales. Macroscopically, a mosaic array of the heated and unheated fish scales can reveal the hidden messages only under the UV illumination. 120 mg of the heated fish scales are also found to effectively remove 91% of RhB from 2 mL of 10-6 M of RhB within 10 mins. Microscopically, the laser-treated sites on the fish scales exhibited the more pronounced orange fluorescence under the green-light excitation after interacting with 10-8 M of RhB for 3 mins as compared to the case in the pristine fish scales. Miniature steganography is also achieved through the laser-treatment processes, and the topic is interesting, and the results showed the new applications of waste resources and RhB pollutant removal. This study is novel and deserves to be considered for the publication. However, the exact mechanism of the luminescence of the modified fish scales has not well described in a detailed way. I strongly recommend the major revision as follows:

1. Without heating, the pristine fish scale depicted in Figure 1(b) exhibits the faint fluorescence under UV excitation. The authors should explain the reason and the mechanism by which the pristine fish

scale can luminesce under UV excitation.

2. A comparison should be made of the changes in the luminescence mechanism of fish scales under UV excitation before and after heating. As we all know, the triple helix steric structure of collagen is altered by heating. In addition, there are luminescent functional groups in natural proteins that can cause fluorescence, and heating also causes intermolecular interaction changes of luminescent functional groups. In this study, the heated fish scales stripped of HAp also showed the good fluorescence properties (Figure 3 (a)), and the authors should explain the chemical changes in the collagen molecules under 270 ℃ of heating that led to the enhanced fluorescence and the change in electron leap state during luminescence.

3. The fluorescence properties of HAp from the fish scales were also improved after heating at 270 °C under the UV excitation. Did you measure the change in the crystalline phases or crystalline structures before and after heating? It is necessary to measure XRD patterns and FTIR spectra of the HAp powder extracted from the fish scales before and after heating to evaluate the chemical changes that occur in HAp.

4. On page 14, the results showed that 3.6 and 1.8 times increase in phosphorous and calcium contents for the heated fish scales, as comparing to the case in the pristine. The reason for the greater magnitude of the increase in the phosphorus as compared with the calcium should be explained. The inorganic/organic hybrid states would be changed.

5. There were black regions in Figure 5 (a), and the reason should be discussed with adding the description on the measurement system.

6. As compared to Figure 6 (c), the cyan luminescence of the fish scales after adsorption of RhB (Figure 6 (h)) was reduced, the reason should be explained.

7. The RhB solution generally exhibits the bright pink fluorescence under the UV excitation, but there was no fluorescence color in the enclosed region treated with RhB under the UV excitation in Figure 6 (g-h). The reason should be explained.

8. The adsorption of RhB occurs on which part of the fish scale should be explained. Whether it occurs only on the limiting layer of the scale. In the case, RhB easily interacts only with hydroxyapatite surfaces. What happens if RhB is adsorbed on HAp extracted from the fish scales and what happens if it is adsorbed onto fish scales from which HAp has been removed? These points were not clear at the present states.

9. I feel the manuscript is not well organized, and there are many mistakes as follows.
(1) The author should define the full name of “IE，LL” where they first appear in Line 196 and197, Page11.
(2) “ART-FTIR” in line 284, Page 14 should be changed to “ATR-FTIR”.
(3) On page 27, “RhB is hard to distinguish with the naked eye beyond 10-6 M”. "beyond" is not a correct expression.

10. The abstract, introduction and conclusion that correspond to the text contents should be revised. Furthermore, the abstract and conclusions should focus more on what the findings of the study mean rather than describing the study again.

Reviewer #3 (Remarks to the Author):

This manuscript submitted for consideration in Nat Comm describes thermal annealing of fish scales to give luminescent materials.

The procedure involves heating the scales between two steel plates (or by laser). According to the authors, the resulting materials have similar morphology to the pristine scales but possess increased fluorescence.
The authors propose using these photoluminescent materials for steganography and RhB detection/sequestration.
In the opinion of this reviewer the manuscript is not suitable for publication for a number of reasons (in order of importance).

1. The insurgence of luminescence observed by annealing organic materials of any kind is necessarily due to chemical reactions occurring on the biomass. These reaction generally cause the formation of moieties (chromophores, unsaturated systems, discrete luminescent molecules, etc.) that are responsible for photoluminescence. In this manuscript the origin of luminescence of the obtained materials is not explained and no evidence or hypothesis of the presence and structure of luminescent systems is described. The complete absence of any explanation as to what is causing photoluminescence is itself reason enough to make the manuscript unsuitable for publication. A proposed insight on the cause of fluorescence is attempted In lines 249-252 by linking collagen denaturation to luminescence, but this is purely phenomenological without any explanation on the cause. On line 285-286 enhanced fluorescence is attributed to “collagen fibers and HAP crystal complexes”, which is also a scientifically unsubstantiated statement.
2. To verify the claim that fluorescence depends on collagen fibers and HAP, the authors separate HAP and collagen in the fish scales (lines 291-293), then heat each component separately and observe enhanced luminescence in both. While it is understandable that heated collagen may become luminescent (although what is causing it is still unexplained), it is rather surprising that pure HAP ($Ca_{10}(PO_4)_6(OH)_2$) becomes luminescent once heated. In the latter case it is reasonable to assume that the extracted HAP still contained organic material able to generate luminescent moieties.
3. On lines 541 the authors state that “the thermal annealing process encourages the formation of molecular moieties attractive to RhB”. I would suggest identifying these moieties before considering publication.
4. On line 265-266 “Heating the fish scale in ambient results in (1) the formation of more O=C=O bonds” seems to indicate that heating fish scales containing carbon under air produces carbon dioxide. This is rather obvious and certainly does not account for luminescence.
5. The proposed application of the laser-treated fish scales as a micro-detector and adsorbent for RhB seems like a potentially interesting application of these materials. Here also however the authors failed to explain the molecular reason why the annealed scales are more RhB-phylic than the pristine counterparts. The experiments described in figure 7 also provide a purely phenomenological view of the system without explaining the underlying interactions that lead to increased adsorption.
6. Using an assembly of fish scales – some heated, some pristine – to form hidden messages as shown in figure 1c-f is puerile. The uncharred annealed scales are visibly different from the pristine material under visible light, therefore any pattern that is observed under UV irradiation is also evident under visible light.
7. Laser etching of the scales to obtain luminescent figures and patterns is certainly decorative but of scarce scientific value as the procedure simply replicates on a smaller scale the previously observed luminescence enhancement obtained by bulk annealing.
8. The final section dedicated to using the system to encode ciphers in painting and sculptures is appealing but should probably find its publication stage elsewhere.

COMMENTS TO AUTHOR:

Reviewer #1 (Remarks to the Author):

In this work, the authors employed abundant and eco-friendly fish scales for the subtle fabrication of bifunctional materials via facile heating treatment for steganography and RhB adsorption applications. This work is interesting and a good storyline is presented in the main text. The majority of the results could be supported by the corresponding experiments. Moreover, the topic of this work is within the scope of the journal. Therefore, this manuscript is recommended to the journal after a Major Revision. The comments are as follows:

Comment 1: What chemical structures or moieties endow the heated fish scales with bright fluorescence emission? Please describe it in detail.

Reply 1: *Thank you for your comment. We have carried out additional experiments; detailed analyses are presented in the revised manuscript. Herein, we would like to provide a summary of our proposed mechanism for the enhanced fluorescence observed in heated fish scales:*

The fluorescence enhancement is contributed by dehydrated and defective collagen and HAP.

When primitive fish scales are heated, structural changes expose the underlying collagen fibers and HAP structures, facilitating the release of physisorbed CO_2, H_2O *and entrapped* H_2O *molecules. The desorption process leads to cracks, increases in porosity, and encourages the substitution of OH groups by intrinsic* CO_3 *impurities. Heating also initiated the denaturation of collagen fibers, forming more blue fluorescence glycyl peptides. Work by Perry et al.*[1] *shows that the intrinsic blue fluorescent (410 nm) from collagen under UV excitation is largely attributed to the presence of glycyl peptides.*

HAP in the fish scale are determined to be slightly carbonated. When heated, distortion to the intrinsic HAP structure due to carbonate impurities and the release of entrapped H_2O *molecules can introduce energy levels within the forbidden zone responsible for electron-hole recombinations, resulting in fluorescence emission not observed from heated pure HAP structures. Machado et al.*[2] *highlight the role that substitutional defects such as* CO_3 *in HAP have towards fluorescent in HAP.*

The shift in fluorescence emitted (from blue to cyan) is attributed to the loss of stabilizing hydrogen bonds which lead to the formation of longer, uncoupled, randomly coiled states from the thermal labile domain (from Gly 887 to Pro941, ie: 65 residues) within the collagen network, resulting in the observed polymer-in-a-box phenomenon. Miles et al.[3] *discuss such transformation to collagen in their work.*

We have included the above description in the revised manuscript and also cited the relevant literatures supporting the claim.

Comment 2: In Figure 2c and lines 282-284, the authors mentioned that "….shows 3.6 times and 1.8 times increase in phosphorous and calcium content from…" Why the contents of P and

Ca greatly increased after heating treatment? Is the charring process of the fish scale? If it is, why the content of C decrease compared to the pristine one? Please give some comments on it.

Reply 2: *Thank you for your comment. With a better understanding of the fluorescence mechanism, we propose the following explanation for the observed increase in P and Ca content after heat treatment:*

The heating process results in structural damage that exposes the underlying collagen fiber and HAP structures. The damage is evident from the SEM image of the same fish scale before and after heating (see below). Hence, the exposure of underlying HAP could contribute to the observed increase in P, and Ca detected.

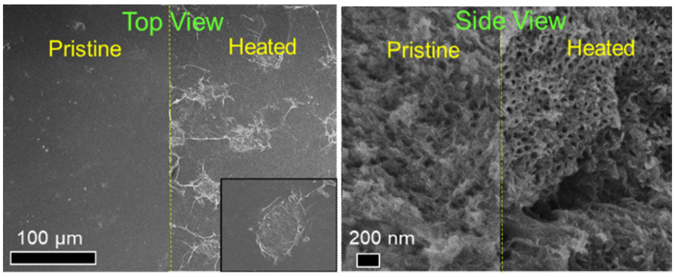

Furthermore, with possible phosphate being substituted by carbonate ions from carbonate impurities within the primitive fish scale during the heating process, phosphate diffusion towards the surface could also lead to a stronger signal being detected, thus accounting for the greater proportion of P compared to Ca.

Comment 3: How long this enhanced fluorescence property could be maintained after heating treatment?

Reply 3: *The enhanced fluorescence property is stable even when observed 5 months after fabrication, as presented in the Supplementary Fig. 12 of the revised manuscript (see below).*

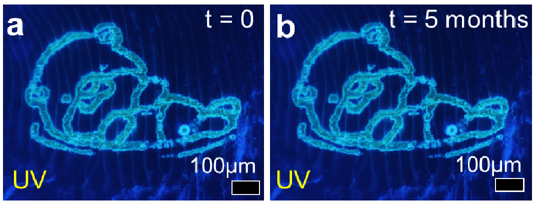

Comment 4: The authors mentioned the effect of temperature on the fluorescence property of the fish scale. However, no related mechanism was described in the main text.

Reply 4: *Thank you for your comment. Based on the proposed fluorescence mechanism, we propose the following for the observed temperature dependency of the observed fluorescence.*

Given the same duration of heating, higher temperature will result in a greater degree of dehydration, hence increasing the amount of H_2O, CO_3-related defects and glycyl peptides, which contributed to the temperature-dependent enhanced thermal induced fluorescence detected.

The above is included in the revised manuscript.

Comment 5: Line 200-202, the authors mentioned that “the most intense and uniform fluorescence enhancement occurs at 270 ^{o}C”. However, in the Figure b, the intensity of fluorescence of heated fish scales at 300 ^{o}C seems higher than that of at 270 ^{o}C. Why?

Reply 5: *Based on the reply to Comment 4, the intensity of the fish scale heated at 300 ^{o}C will exhibit strong fluorescence from the uncharred region. However, the fish scale will experience some charring at this temperature, reducing the fluorescence emitted at the charred site. The reduction in fluorescence intensity is depicted in Supplementary Fig. 1, where the exposure time of image capture is intentionally reduced to highlight the loss of fluorescence due to the charring effect. Furthermore, charring makes the fish scale very brittle and unsuitable for use in the applications studied in this work.*

Comment 6: Lin 300, “collagen shows a 5-fold and 10-fold increase in PL for both …..” Please explain this result. Meanwhile, Line 312, the authors mentioned that “collagen has a greater contribution to the observed fluorescence enhancement” Any references or experiments could support this conclusion?

Reply 6: *From the newly included TGA and DTG results, while most of the HAP has been extracted, a significant amount exists in the remaining collagen structure. On the other hand, no significant collagen-related peaks from the FTIR analysis of HAP extracts are detected. The results thus suggest that stronger fluorescence from collagen extract likely arises from a combinatory effect of heated HAP and collagen. The new results are included in Fig. 2 of the revised manuscript.*

Comment 7: The adsorption capacity and behavior of the heated fish scales toward RhB is suggested to investigate in detail including kinetics, isotherm and effect factors et al.

Reply 7: *Thank you for your suggestion. We have conducted further experiments and have included the RhB biosorption isotherms on heated fish scales in Fig. 8d of our revised manuscript.*

Comment 8: The mechanism of the heated fish scales toward RhB should also be a proposal, although the authors explored the adsorption behavior of the heated fish scales through XPS test.

Reply 8: *Thank you for highlighting this. From the RhB biosorption isotherms on heated fish scales presented in Fig. 8d of our revised manuscript and supporting experiments such as porosity and desorption of RhB (Fig. 8 of the revised manuscript), we proposed that the adsorption process of RhB on heated fish scales likely involved multimolecular layer formation in mesopores (diameter: 2-50 nm) with both strong and weak adsorbate-adsorbent interactions. This proposal is based on an adaption of the Dubinin-Astakov model proposed by Blázquez et al.*[4] *Details of the proposed adsorption mechanism are included in the revised manuscript.*

Comment 9: How about the adsorption capacity of the heated fish scales toward other organic dyes? Please check it.

Reply 9**:** *Thank you for this suggestion. 120 mg of heated fish scales are determined to be capable of removing 64% of methylene blue within 3 mins. The result, which also shows the versatility of heated fish scale as a biosorbent, is included in the revised manuscript (Supplementary Fig. 16)*

Comment 10: How to achieve the desorption of the RhB-adsorbed fish scales? How about the recyclability of this biosorbent?

Reply 10**:** *Thank you for bringing up this point. We have investigated and shown in Fig. 8 of the revised manuscript that the heated fish scale can be recovered through a simple ultra-sonication process in DI* H_2O*. The recovery process also works well for methylene blue molecules (Supplementary Fig. 16). We have also conducted repeated adsorption-desorption tests, and the results are favourable towards the recyclability of fish scale as a biosorbent (Fig. 8 of the revised manuscript).*

Reviewer #2 (Remarks to the Author):

In this paper, the authors investigated the enhanced fluorescence and RhB adsorbent properties of the heated fish scales. Macroscopically, a mosaic array of the heated and unheated fish scales can reveal the hidden messages only under the UV illumination. 120 mg of the heated fish scales are also found to effectively remove 91% of RhB from 2 mL of 10-6 M of RhB within 10 mins. Microscopically, the laser-treated sites on the fish scales exhibited the more pronounced orange fluorescence under the green-light excitation after interacting with 10-8 M of RhB for 3 mins as compared to the case in the pristine fish scales. Miniature steganography is also achieved through the laser-treatment processes, and the topic is interesting, and the results showed the new applications of waste resources and RhB pollutant removal. This study is novel and deserves to be considered for the publication. However, the exact mechanism of the luminescence of the modified fish scales has not well described in a detailed way. I strongly recommend the major revision as follows:

Comment 1: Without heating, the pristine fish scale depicted in Figure 1(b) exhibits the faint fluorescence under UV excitation. The authors should explain the reason and the mechanism by which the pristine fish scale can luminesce under UV excitation.

Reply 1: *Thank you for the comment. Work by Perry et al.*[1] *shows that the intrinsic blue fluorescent (410 nm) from collagen under UV excitation is largely attributed to the presence of glycyl peptides. At the same time, Machado et al.*[2] *highlight the role that substitutional defects such as* CO_3 *in HAP have towards fluorescent in HAP. Considering that the fish scale comprises mainly of these two materials, and pure HAP is tested not to exhibit blue fluorescence even when heated (Supplementary Fig. 8), we thus attribute the intrinsic fluorescence in fish scales to the factors mentioned above.*

Comment 2: A comparison should be made of the changes in the luminescence mechanism of fish scales under UV excitation before and after heating. As we all know, the triple helix steric structure of collagen is altered by heating. In addition, there are luminescent functional groups in natural proteins that can cause fluorescence, and heating also causes intermolecular interaction changes of luminescent functional groups. In this study, the heated fish scales stripped of HAp also showed the good fluorescence properties (Figure 3 (a)), and the authors should explain the chemical changes in the collagen molecules under 270 °C of heating that led to the enhanced fluorescence and the change in electron leap state during luminescence.

Reply 2: *Thank you for highlighting this. We have conducted additional experiments; detailed analyses are presented in the revised manuscript. Herein, we would like to provide a summary of our proposed mechanism for the enhanced fluorescence observed in heated fish scales:*

The fluorescence enhancement is contributed by dehydrated and defective collage and HAP. When primitive fish scales are heated, structural changes expose the underlying collagen fibers and HAP structures, facilitating the release of physisorbed CO_2*,* H_2O *and entrapped* H_2O *molecules. The desorption process leads to cracks, increases in porosity, and encourages the*

substitution of OH groups by intrinsic CO_3 impurities. Heating also initiated the denaturation of collagen fibers, forming more blue fluorescence glycyl peptides.

HAP in the fish scale are determined to be slightly carbonated. When heated, distortion to the intrinsic HAP structure due to carbonate impurities and release of entrapped H_2O molecules can introduce more energy levels within the forbidden zone responsible for electron-hole recombinations, resulting in the fluorescence emission not observed from heated pure HAP structures.

The shift in fluorescence emitted (from blue to cyan) is attributed to the loss of stabilizing hydrogen bonds which lead to the formation of longer, uncoupled, randomly coiled states from the thermal labile domain (from Gly 887 to Pro941, ie: 65 residues) within the collagen network, resulting in the observed polymer-in-a-box phenomenon.

We have included the above description in the revised manuscript.

Comment 3: The fluorescence properties of HAp from the fish scales were also improved after heating at 270 °C under the UV excitation. Did you measure the change in the crystalline phases or crystalline structures before and after heating? It is necessary to measure XRD patterns and FTIR spectra of the HAp powder extracted from the fish scales before and after heating to evaluate the chemical changes that occur in HAp.

Reply 3: *Thank you for the suggestion; we have obtained new XRD and FTIR data of the extracts, and they are presented below:*

XRD

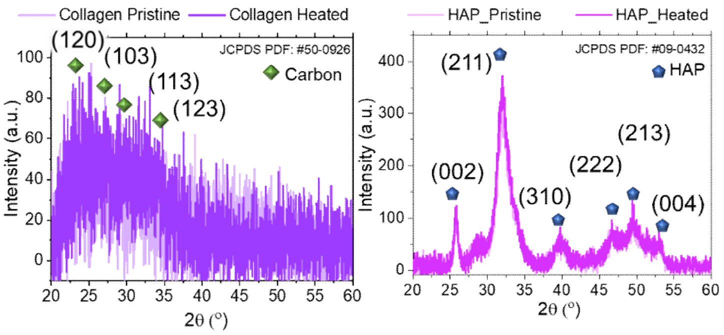

Compared between pristine and heated collagen and HAP extracts, the temperature and duration applied to the extracts appear insufficient to cause any significant difference in collagen extract's crystallinity. In contrast, a very slight improvement in crystallinity is detected from the heated HAP extract.

FTIR

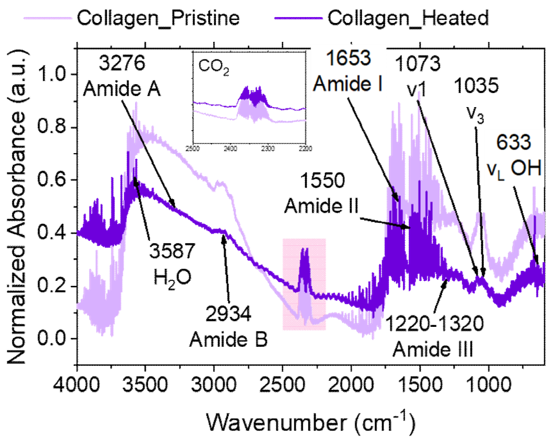

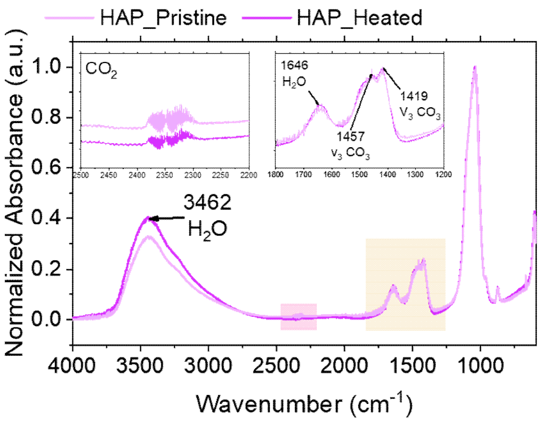

FTIR from heated collagen exhibits a drastic reduction in all vibrational modes identified above, except for CO_2 where stronger absorbance is detected. Thus, suggesting that thermal annealing results in the decomposition of both the collagen protein and the remaining HAP in the collagen extract. The detection of more CO_2 on the heated collagen extract can be due to releasing of physisorbed CO_2 from the remaining HAP within the collagen structure and atmospheric CO_2 adsorption. The release of physisorbed CO_2 from heated HAP is justified by the FTIR of heated HAP, which also shows more physisorption of H_2O molecules, possibly due to more dangling bonds from the dehydrated samples that served as adsorption sites for the atmospheric H_2O molecules. A subtle increase in the vibrational modes of CO_3 groups substituting OH (A-type) and PO_4 (B-type) groups from the heated HAP samples is also detected.

The above discussions are included in the revised manuscript.

Comment 4: On page 14, the results showed that 3.6 and 1.8 times increase in phosphorous and calcium contents for the heated fish scales, as comparing to the case in the pristine. The reason for the greater magnitude of the increase in the phosphorus as compared with the calcium should be explained.

Reply 4: *Thank you for your comment. With a better understanding of the fluorescence mechanism, we propose the following explanation for the observed increase in P and Ca content after heat treatment:*

The heating process results in structural damage that exposes the underlying collagen fiber and HAP structures. The damage is evident from the SEM image of the same fish scale before and after heating (see below). Hence, the exposure of underlying HAP could contribute to the observed increase in P, and Ca detected.

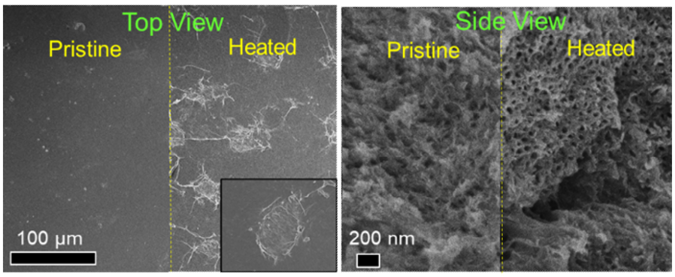

Furthermore, with possible phosphate being substituted by carbonate ions from carbonate impurities within the primitive fish scale during the heating process, phosphate diffusion towards the surface could also lead to a stronger signal being detected, thus accounting for the greater proportion of P compared to Ca.

Comment 5: There were black regions in Figure 5 (a), and the reason should be discussed with adding the description on the measurement system.

Reply 5: *Thank you for the suggestion. From the figure below, the focused laser's Gaussian beam profile (a) highlights the existence of a unique heat zone that facilitates the formation of fluorescing active species such as glycyl peptides and substitutional carbonate impurities (cyan fluorescence). In the region far from the laser spot, the pristine section (blue fluorescence) of both SEM and BEI images (b-c) shows alternating multi-layered collagen fibers and HAP crystals. Within the cyan fluorescing zone, collagen and HAP denature, with the HAP crystals merging to form a distinct layer between two collagen mash (b, d). In the region closest to the laser beam (e), the lack of fluorescence is attributed to the rapid, intense heating from the laser, which readily breakdowns molecular bonds and decomposes impurities such as carbonate substitutes and entrapped H_2O molecules within both Gly-X-Y and HAP structures. The quick removal of the laser from the annealing spot leads to fast cooling, leaving the laser-treated region in a quenched state with minimal molecular restructuring.*

The above discussion is included in the revised manuscript.

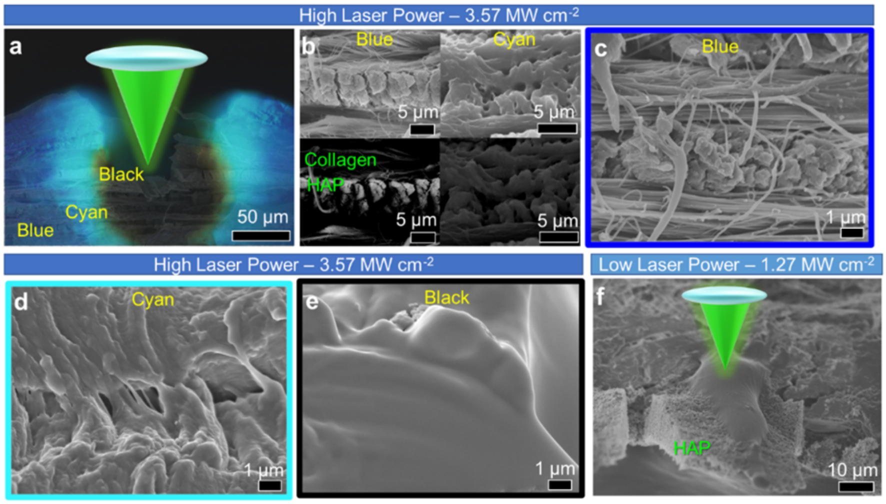

Comment 6: As compared to Figure 6 (c), the cyan luminescence of the fish scales after adsorption of RhB (Figure 6 (h)) was reduced, the reason should be explained.

Reply 6: *In this experiment, a droplet of 10^{-8} M of RhB is placed on the fish scale for 3 minutes and washed off with DI water before being blown dry with N_2 gas. At this concentration, the solution emits very dim fluorescent under UV, while a very slightly orange fluorescent is observed under G excitation when placed on a silicon wafer (see below).*

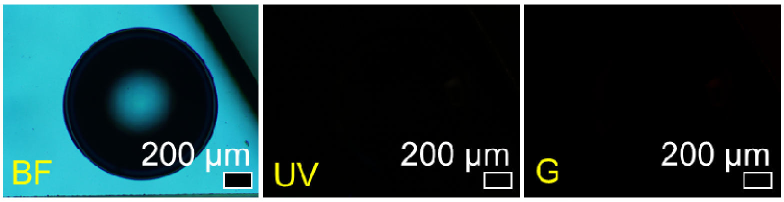

Under UV excitation, the "Rose" pattern appears dimmer, likely due to adsorbed RhB molecules that are not readily excited by UV light at this concentration.

The above discussion is included in the revised manuscript.

Comment 7: The RhB solution generally exhibits the bright pink fluorescence under the UV excitation, but there was no fluorescence color in the enclosed region treated with RhB under the UV excitation in Figure 6 (g-h). The reason should be explained.

Reply 7: *Indeed, at higher concentrations, RhB solution does take on a pink fluorescence under UV excitation (Fig.7a of the revised manuscript). However, as mentioned in reply to comment 6, at a lower concentration, RhB solution placed on the "Rose" pattern emits very dim fluorescent under UV; hence, the fluorescence is not readily detected in the enclosed region treated with RhB under UV excitation.*

Comment 8.1: The adsorption of RhB occurs on which part of the fish scale should be explained. Whether it occurs only on the limiting layer of the scale. In the case, RhB easily interacts only with hydroxyapatite surfaces.

Reply 8.1: *From the cross-sectional FM images and the PL spectra obtained from the LL and IE of the RhB-treated heated fish scale (Fig. 7c, e) of the revised manuscript), there appears to be no significant difference between the fluorescence detected between the LL and IE region. The results show no apparent preferential adsorption of RhB molecules to the different regions of the heated fish scales.*

Comment 8.2: What happens if RhB is adsorbed on HAp extracted from the fish scales and what happens if it is adsorbed onto fish scales from which HAp has been removed? These points were not clear at the present states.

Reply 8.2: *A droplet of 2 µL of 10^{-6} M RhB is placed on collagen extracts for 3 mins before rinsing with DI H_2O, while the HAP extract readily adsorbs a similar droplet of RhB solution (see below). This result is included in Fig. 8 of the revised manuscript). The heated form of both extracts exhibits stronger fluorescence attributed to the presence of more RhB molecules*

than pristine extracts, whose adsorption mechanism is proposed to be an adaption of Dubinin-Astakov model proposed by Blázquez et al.[4] The model suggests that the adsorption process of RhB on heated fish scales likely involved multimolecular layer formation in mesopores (diameter: 2-50 nm) with both strong and weak adsorbate-adsorbent interactions. Details of the verifications of the proposed adsorption mechanism are presented in the revised manuscript.

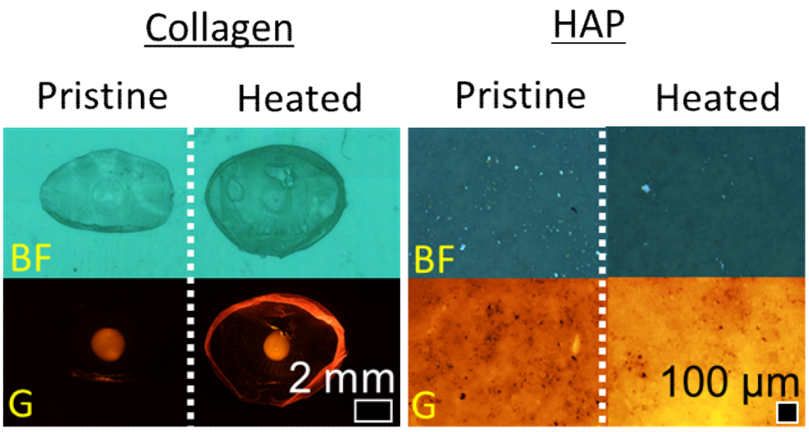

Comment 9: I feel the manuscript is not well organized, and there are many mistakes as follows.
(1) The author should define the full name of "IE，LL" where they first appear in Line 196 and197, Page11.

(2) "ART-FTIR" in line 284, Page 14 should be changed to "ATR-FTIR". (3) On page 27, "RhB is hard to distinguish with the naked eye beyond 10-6 M". "beyond" is not a correct expression.

Reply 9: *Thank you for pointing these out. We have made the necessary corrections and have conducted a thorough check of the revised manuscript to minimize such errors.*

(1) The definitions are listed in Pg. 9 together with Supplementary Fig. 4, which defines different regions of the fish scale's top view and cross-sectional view.
(2) We have verified that the FTIR spectrometer model used is FTIR Vertex 70 attached to an upright microscope) and due to the breakdown of the original system, additional FTIR data presented in the revised manuscript are obtained using IRPrestige-21 FTIR spectrometer.
(3) We have removed the above incorrect expression in the revised manuscript.

Comment 10: The abstract, introduction and conclusion that correspond to the text contents should be revised. Furthermore, the abstract and conclusions should focus more on what the findings of the study mean rather than describing the study again.

Reply 10: *Thank you for your comment. We have revised the abstract, introduction and conclusion in the revised manuscript. They now placed more emphasis on the significance of our reported work.*

Reviewer #3 (Remarks to the Author):

This manuscript submitted for consideration in Nat Comm describes thermal annealing of fish scales to give luminescent materials.

The procedure involves heating the scales between two steel plates (or by laser). According to the authors, the resulting materials have similar morphology to the pristine scales but possess increased fluorescence. The authors propose using these photoluminescent materials for steganography and RhB detection/sequestration. In the opinion of this reviewer the manuscript is not suitable for publication for a number of reasons (in order of importance).

Comment 1: The insurgence of luminescence observed by annealing organic materials of any kind is necessarily due to chemical reactions occurring on the biomass. These reaction generally cause the formation of moieties (chromophores, unsaturated systems, discrete luminescent molecules, etc.) that are responsible for photoluminescence. In this manuscript the origin of luminescence of the obtained materials is not explained and no evidence or hypothesis of the presence and structure of luminescent systems is described. The complete absence of any explanation as to what is causing photoluminescence is itself reason enough to make the manuscript unsuitable for publication. A proposed insight on the cause of fluorescence is attempted In lines 249-252 by linking collagen denaturation to luminescence, but this is purely phenomenological without any explanation on the cause. On line 285-286 enhanced fluorescence is attributed to “collagen fibers and HAP crystal complexes”, which is also a scientifically unsubstantiated statement.

Reply 1: *Thank you for highlighting this. We have conducted more literature reviews and experiments, allowing us to understand the system better. We provide a summary of our proposed mechanism for the cause of heat-induced fluorescence enhancement to the heated fish scales below:*

The fluorescence enhancement is contributed by dehydrated and defective collagen and HAP.

When primitive fish scales are heated, structural changes expose the underlying collagen fibers and HAP structures, facilitating the release of physisorbed CO_2, H_2O and entrapped H_2O molecules. The desorption process leads to cracks, increases in porosity, and encourages the substitution of OH groups by intrinsic CO_3 impurities. Heating also initiated the denaturation of collagen fibers, forming more blue fluorescence glycyl peptides. Work by Perry et al.[1] shows that the intrinsic blue fluorescent (410 nm) from collagen under UV excitation is largely attributed to the presence of glycyl peptides.

HAP in the fish scale are determined to be slightly carbonated. When heated, distortion to the intrinsic HAP structure due to carbonate impurities and the release of entrapped H_2O molecules can introduce energy levels within the forbidden zone responsible for electron-hole recombinations, resulting in fluorescence emission not observed from heated pure HAP structures. Machado et al.[2] highlight the role that substitutional defects such as CO_3 in HAP have towards fluorescent in HAP.

The shift in fluorescence emitted (from blue to cyan) is attributed to the loss of stabilizing hydrogen bonds which lead to the formation of longer, uncoupled, randomly coiled states from the thermal labile domain (from Gly 887 to Pro941, ie: 65 residues) within the collagen

network, resulting in the observed polymer-in-a-box phenomenon. Miles et al.[3] *discuss such transformation to collagen in their work.*

Detailed discussions and experimental results are presented in the revised manuscript.

Comment 2: To verify the claim that fluorescence depends on collagen fibers and HAP, the authors separate HAP and collagen in the fish scales (lines 291-293), then heat each component separately and observe enhanced luminescence in both. While it is understandable that heated collagen may become luminescent (although what is causing it is still unexplained), it is rather surprising that pure HAP ($Ca_{10}(PO_4)_6(OH)_2$) becomes luminescent once heated. In the latter case it is reasonable to assume that the extracted HAP still contained organic material able to generate luminescent moieties.

Reply 2: *As mentioned in the above reply to comment 1, the cause of heated collagen to luminescent is attributed to the formation of more blue fluorescence glycyl peptides as verified by Perry et al.*[1] *At the same time, Machado et al.*[2] *highlight the role that substitutional* CO_3 *defects can contribute towards fluorescent in HAP. Considering that the fish scale comprises mainly of these two materials, and pure HAP is tested not to exhibit blue fluorescence even when heated (Supplementary Fig. 8), we attribute the enhanced fluorescence from the heated fish scales to the formation of more glycyl peptides and the introduction of more energy levels within the forbidden zone of HAP which are responsible for electron-hole recombinations by the substitutional defects such as* CO_3 *(found intrinsically in the primitive fish scale). Detailed discussion is presented in the revised manuscript.*

Comment 3: On lines 541 the authors state that "the thermal annealing process encourages the formation of molecular moieties attractive to RhB". I would suggest identifying these moieties before considering publication.

Reply 3: *Based on further experiments conducted and results presented in the revised manuscript, we propose the following:*

As an amphoterism dye, RhB contains positive and negative charges, suggesting it can readily interact with PO_4^{3-}, Ca^{2+} *and oxide-related bonds. The intrinsic nature of the RhB molecules also allows it to contribute to forming more C-C/C-H bonds.*

The above proposal is verified by investigating extracted collagen and HAP towards the adsorption of RhB. The heated form of both extracts exhibits stronger fluorescence attributed to more RhB molecules than pristine extracts. Through sorption isotherm analysis of RhB onto heated fish scales, the result agrees with the adaption of Dubinin-Astakov model proposed by Blázquez et al,[4] *which suggests that the adsorption process of RhB on heated fish scales likely involved multimolecular layer formation in mesopores (diameter: 2-50 nm) with both strong and weak adsorbate-adsorbent interactions.*

The distribution plot of pores diameter from pristine and heated fish scales falls within the range of the proposed model. Furthermore, heating results in the formation of larger pores which facilitates diffusions of RhB solution into the fish scale structure, encouraging greater

uptake of RhB molecules from the solution. Interactions between RhB and heated fish scale is investigated by sending the same piece of RhB adsorbed heated fish scale through multiple rounds of adsorption and sonication in DI H_2O. With weak fluorescent detected after sonication of heated fish scales, the outcome agrees with the above model that the adsorption of RhB onto heated fish scale involved strong and weak interactions.

Comment 4: On line 265-266 "Heating the fish scale in ambient results in (1) the formation of more O=C=O bonds" seems to indicate that heating fish scales containing carbon under air produces carbon dioxide. This is rather obvious and certainly does not account for luminescence.

Reply 4: Indeed, CO_2 alone does not contribute to the observed luminescence of heated fish scales under UV excitation. However, when primitive fish scales are heated at 270 °C, the material exhibits structural decomposition, forming porous structures that expose the underlying collagen fibers and HAP structures (revised manuscript, Fig. 3e-f). The exposure facilitates the release of physisorbed CO_2, H_2O and entrapped H_2O molecules (revised manuscript Fig. 2c), which results in the formation of cracks, increase in porosity (revised manuscript Fig. 3f), changes intercrystalline locations and encourages substitution of OH groups by intrinsic CO_3 impurities (revised manuscript Fig.3 a-c). The structural changes also allow thermal dissipation to reach beyond the surface, forming more glycyl peptides and introducing more energy levels within the forbidden zone of HAP by the substitutional CO_3 impurities. All these effects likely contributed to the overall fluorescence enhancement observed.

Comment 5: The proposed application of the laser-treated fish scales as a micro-detector and adsorbent for RhB seems like a potentially interesting application of these materials. Here also however the authors failed to explain the molecular reason why the annealed scales are more RhB-phylic than the pristine counterparts. The experiments described in figure 7 also provide a purely phenomenological view of the system without explaining the underlying interactions that lead to increased adsorption.

Reply 5: *Being an amphoteric dye, RhB contains both positive and negative charges, which suggests that they can readily interact with PO_4^{3-}, Ca^{2+} and oxide-related bonds. Heating of fish scale leads to the exposure of underlying collagen fiber and HAP structures due to structure damage sustained by fish scale during the heating process. Furthermore, the increase in pore size of the heated fish scale (revised manuscript Fig. 3f) also facilitates the diffusion of the solution that allows more uptake of RhB molecules within the heated fish scale. As a result, the annealed scales become much more PhB-phylic than the pristine counterparts.*

From the sorption isotherm analysis of RhB on heated fish scales (revised manuscript Fig. 8d), the results show that the adsorption of RhB to the heated fish scale is akin to the adapted Dubinin-Astakov model proposed by Blázquez et al,[4] which suggests that the adsorption process of RhB on heated fish scales involved multimolecular layer formation in mesopores (diameter: 2-50 nm) with both strong and weak adsorbate-adsorbent interactions. The enhanced adsorption towards the heated fish scale can be attributed to the exposure of more adsorption sites in conjunction with more active adsorption sites from the exposed underlying collagen and HAP structure upon annealing (revised manuscript Fig. 3e). Furthermore, the

increase in pore size of the heated fish scale (revised manuscript Fig. 3f) also facilitates the diffusion of the solution that allows more uptake of RhB molecules within the heated fish scale.

Comment 6: Using an assembly of fish scales – some heated, some pristine – to form hidden messages as shown in figure 1c-f is puerile. The uncharred annealed scales are visibly different from the pristine material under visible light, therefore any pattern that is observed under UV irradiation is also evident under visible light.

Reply 6: *Thank you for highlighting this. We have re-created other structures using an array of heated (uncharred) and pristine fish scales. We hope the stegano is now better concealed. Please find the revised Fig. 1 image below.*

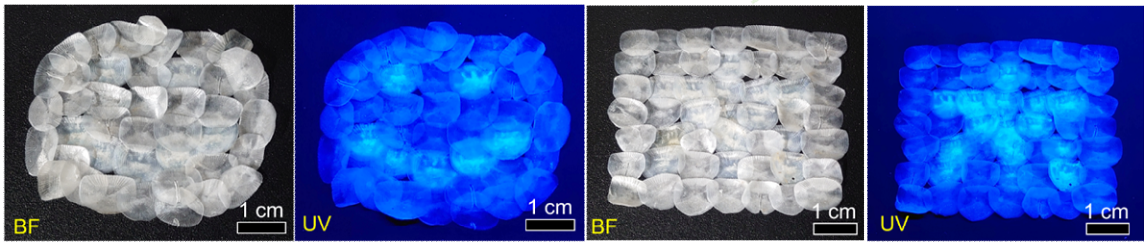

Comment 7: Laser etching of the scales to obtain luminescent figures and patterns is certainly decorative but of scarce scientific value as the procedure simply replicates on a smaller scale the previously observed luminescence enhancement obtained by bulk annealing.

Reply 7: *Thank you for bringing up this point. Apart from decorative purposes, we believe a focused laser beam allows site-selective microscopic regions with controlled fluorescence enhancement to be achieved on a single piece of fish scale. This work has shown that a heated fish scale exhibits greater adsorption efficiency. The controlled laser patterning process thus provides a means for creating a lab-on-a-chip micro-detector from a primitive fish scale.*

Furthermore, the versatility of the laser patterning process in creating different patterns also allows the creation of micro-detectors shaped as unique markers, thus allowing many micro-detectors to be created on a single fish scale. The miniature size of the micro-detector means that only a very small volume of pollutant is required for testing to be carried out, thus reducing the risk of potential exposure to the pollutant.

Comment 8: The final section dedicated to using the system to encode ciphers in painting and sculptures is appealing but should probably find its publication stage elsewhere.

Reply 8: *We believe that merging science with art is a very effective means of science communication. Through the painting and sculpture presented at the end of the manuscript, the steganographic application of this work is effortlessly showcased to readers who may or may not be in the field of steganography.*

Reference

1 Perry, A. J. & Hefferre.Jj. Fluorescence of glycyl peptides and collagen degradation mixtures. *Calcified Tissue Research* **11**, 205-208 (1973).

2 Machado, T. R. *et al.* Designing biocompatible and multicolor fluorescent hydroxyapatite nanoparticles for cell-imaging applications. *Materials Today Chemistry* **14** (2019). https://doi.org:10.1016/j.mtchem.2019.100211

3 Miles, C. A. & Ghelashvili, M. Polymer-in-a-box mechanism for the thermal stabilization of collagen molecules in fibers. *Biophysical Journal* **76**, 3243-3252 (1999). https://doi.org:10.1016/s0006-3495(99)77476-x

4 Blazquez, G., Calero, M., Hernainz, F., Tenorio, G. & Martin-Lara, M. A. Equilibrium biosorption of lead(II) from aqueous solutions by solid waste from olive-oil production. *Chemical Engineering Journal* **160**, 615-622 (2010). https://doi.org:10.1016/j.cej.2010.03.085

REVIEWER COMMENTS

Reviewer #1 (Remarks to the Author):

In this revised manuscript, the authors addressed the reviewers' comments well by point to point. Therefore, it is recommended to journal at the current version without further revision.

Reviewer #2 (Remarks to the Author):

[Note from the editor: please also see attached PDF]

In this paper, the authors investigated the enhanced fluorescence and RhB adsorbent properties of the heated fish scales. Macroscopically, a mosaic array of the heated and unheated fish scales can reveal the hidden messages only under the UV illumination. I reviewed this paper again. However, it is better not to accept the present state because of the lack of novelty and the unclear conception for the application. As for the data in the main text, the data for the flow in the purpose was completely taken in this revision process, but the strong impression on the merely analytical papers will be given to the readers, and I feel that the content is unsuitable for the magazine. If other judges have strong opinions to be considered for the publication, I think the authors should revise the novelty and deserves, and I strongly recommend the reject or the major revision as follows:

1. The TG curves in Figure 2b and 2c only measured the collagen and HAp extracted from fish scales, but it is unclear if these components alone are responsible for the enhanced fluorescence and adsorption properties due to the heating. To better understand the effects of impurities on the phenomena, it would be useful to measure the TG curves for the entire and nature fish scales.

2. The TGA result of pristine collagen extract (Fig. 2b) was added, however, the authors did not explain the reasons about the cause of mass loss at each temperature stage, especially, the mass loss of the collagen at the stage of 250-386 ℃. At this temperature stage, the physicochemical structural change of the collagen is mostly possible to be the main reason for the fluorescence enhancement.

3. In the XRD patterns of collagen and HAp, the diffraction peaks before and after the heating overlapped together, and the diffraction peaks after the heating covered over the diffraction peaks before heating. Thus, it is difficult to discuss the difference. For the collagen diffraction peaks (Fig. 2d), it is difficult to determine the crystal planes due to too much noise. For the HAp diffraction peak (Fig .2e), the increase in the crystallinity after the heating needs to be determined by specifying FWHM.

4. For the FT-IR results of collagen and HAp before and after the heating, since the absorbance obtained by the FT-IR spectra will be changed according to the amount of the measured sample. Thus, it is unsuitable to directly compare the absorbance before and after the heating, and it is necessary to find the reference band and unify the intensities of the reference peaks. Then, the authors should compare the relative intensities and the reference peak. In the wavenumber between 2000 and 2500 (cm-1) attributed to the carbon dioxide component in the air, it is not suitable to judge using the carbon dioxide component existing in the structure. If you want to evaluate the carbon dioxide component, it is recommended to separately compare the absorption band of the carbonate group. For Fig. 3a, there is too much noise in the FTIR to see the exact absorption band, and please analyze in smooth the data. For the fish scales before and after the heating (Fig. 3c), the absorption bands between wavenumber 600 and 900 show the change that is not discussed, and this part of the HAp structural change is probably linked to the luminescence mechanism. This point is important in the paper and the authors should analyze.

5. Based on the absorption peaks at 1419 and 1457 (cm-1) in Figure 2h, the author assumed that the

HAp composition was AB-co-substituted carbonated HAp. However, the peak near 1457 (cm-1) is also present in B-type carbonated HAp and cannot be used to determine the HAp composition. The absorption peak around 1539 (cm-1) should be used to confirm the presence of type A carbonate ions.

6. The authors explained that the fluorescence enhancement is contributed by the dehydrated and defective collagen and HAp. These explanations are lopsided and lack of convince, and the reason is possible due to the special complex chemical structure formed between collagen and HAp. In particular, at near 270°C, the chemical structure of collagen begins to change and then the fluorogenic chemical group of collagen is possibly modified to the surface of HAp and then to form the special composite structure. As a result, the fluorescence would be enhanced. The most significant cause of the enhanced fluorescence is probably derived from collagen rather than HAp. The authors should analyze and discuss the mechanisms.

7. The mechanism of luminescence needs the further investigation. Comparing the fluorescence spectra in the paper with those in other studies reveals the differences in the spectral shapes, especially, at the fluorescence wavelength of 410 nm for collagen in the paper, which is around 530 nm in the other studies.

8. The fluorescence intensity at 395-420 nm, which causes the blue fluorescence, is lower in the paper. Therefore, it is doubtful whether the fluorescence color can be considered blue.

9. No clear structural changes in collagen and HAp due to the heating were observed. Therefore, the enhancement of fluorescence intensity may not be due to structural changes, but rather the external factors such as an increase in excitation intensity due to impurity removal. Additional measurements should be conducted to further evaluate these external factors.

10. The authors explained that the reason for the increase in phosphate ion content being greater than calcium ion content was due to an increase in the carbonate ion substitution in HAp, but this was not evident in the presented data. In other words, the increase in the content is thought to be due to the abundance ratio of calcium and phosphate ions in the initial state. Therefore, I suggest presenting not only the increase in ion content but also the content of calcium and phosphate ions in the initial state, and then comparing then with the data obtained after the heating.

11. In Figure 7c, instead of comparing the photos under brightfield illumination, why not compare the photos under UV and green excitation, and discuss the locational sites of fluorescence due to the heating and RhB adsorption?

12. The abstract, introduction and conclusion that correspond to the text contents should be revised. Furthermore, the abstract and conclusions should focus more on what the findings of the study mean rather than describing the study again. For example, the following points should be focused.
12-1. I am having the trouble to understand the novelty of this research. It would be helpful if the introduction emphasized the superiority of this research compared to existing materials for steganography and dye adsorption.
12-2. I am unsure why you are aiming to apply your research to two very different applications, steganography materials and dye adsorbents. It would be beneficial if the introduction explained the advantages of having a material that can serve both purposes, after listing the necessary properties for each application.

Reviewer #3 (Remarks to the Author):

In the opinion of this reviewer, manuscripts that aim to be published in top journals need to contain highly original research in groundbreaking fields, that hold promise for exceptional developments and significant advance of science.

As already stated in my first round of review this manuscript does not meet any of these criteria.
Annealing of organic materials to obtain luminescent materials is already known and well described, as also confirmed by the authors in reply to the request by the previous reviewers to explain the origin of luminescence.
The proposed applications as detectors and adsorbents for RhB do not represent a groundbreaking application.
Finally, the hidden messages, the patterns and tha applications in ciphering seem like scarcely significant both from a scientific as well as from an artistic or applicative point of view.
I do not recommend publication in Nature Communications.

REVIEWER COMMENTS

Reviewer #1 (Remarks to the Author):

Comment 1: In this revised manuscript, the authors addressed the reviewers' comments well by point to point. Therefore, it is recommended to journal at the current version without further revision.

Reply 1: *Thank you for recommending our revised manuscript for publication in the journal without further revision.*

_ _ _ _ _ _

Reviewer #2 (Remarks to the Author):

In this paper, the authors investigated the enhanced fluorescence and RhB adsorbent properties of the heated fish scales. Macroscopically, a mosaic array of the heated and unheated fish scales can reveal the hidden messages only under the UV illumination. I reviewed this paper again. However, it is better not to accept the present state because of the lack of novelty and the unclear conception for the application. As for the data in the main text, the data for the flow in the purpose was completely taken in this revision process, but the strong impression on the merely analytical papers will be given to the readers, and I feel that the content is unsuitable for the magazine. If other judges have strong opinions to be considered for the publication, I think the authors should revise the novelty and deserves, and I strongly recommend the reject or the major revision as follows:

Comment 1: The TG curves in Figure 2b and 2c only measured the collagen and HAp extracted from fish scales, but it is unclear if these components alone are responsible for the enhanced fluorescence and adsorption properties due to the heating. To better understand the effects of impurities on the phenomena, it would be useful to measure the TG curves for the entire and nature fish scales.

Reply 1: *TG curves of the entire nature fish scales (labelled as pristine) show predominately release of adsorbed H_2O, physisorbed CO_2, and degradation of collagen and HAp, which are in good agreement as that observed in TG curves from the separate entities of collagen and HAp. In the revised manuscript, we have included three separate TG curves from pristine fish scales, extracted collagen and HAp extract. The possible reason for the respective mass loss is indicated in the individual graphs. TG of pristine and collagen extract exhibited similar mass loss attributed to collagen and HAp in both samples. In the case of TG from HAp, no onset of mass loss attributed to collagen degradation is observed, and more distinct mass losses from the degradation of carbonate groups are detected.*

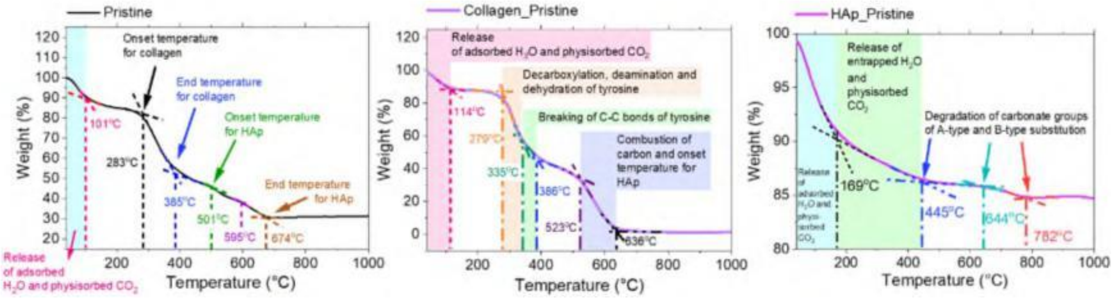

Fig 1. TG curves of pristine fish scale, pristine collagen extract and pristine HAp extract.

Comment 2: The TGA result of pristine collagen extract (Fig. 2b) was added, however, the authors did not explain the reasons about the cause of mass loss at each temperature stage, especially, the mass loss of the collagen at the stage of 250-386 °C. At this temperature stage, the physicochemical structural change of the collagen is mostly possible to be the main reason for the fluorescence enhancement.

Reply 2: *Indeed, a more detailed analysis of the TG results from collagen extract with emphasis at the range of 250-386°C suggests that the mass loss in this range can be attributed decomposition of tyrosine through decarboxylation, deamination, dehydration, and breaking of C-C bonds resulting in the release of gases like H_2O and CO_2.[1] The TG result is in good agreement with both the FTIR and XPS results presented in our work. As such, likely, the presence of tyrosine and tyrosine-like cross-link fluorescence chromophores within the collagen structure contributed to the enhanced fluorescence in the thermally treated fish scales. The plots and analyses presented in Reply 1 and 2 are included in the revised manuscript.*

Comment 3: In the XRD patterns of collagen and HAp, the diffraction peaks before and after the heating overlapped together, and the diffraction peaks after the heating covered over the diffraction peaks before heating. Thus, it is difficult to discuss the difference. For the collagen diffraction peaks (Fig. 2d), it is difficult to determine the crystal planes due to too much noise. For the HAp diffraction peak (Fig .2e), the increase in the crystallinity after the heating needs to be determined by specifying FWHM.

Reply 3: *We have collected new XRD data from collagen and HAp extracts with better resolutions. Peaks of collagen and HAp scans are now more clearly presented. The results are depicted below and included in the revised manuscript.*

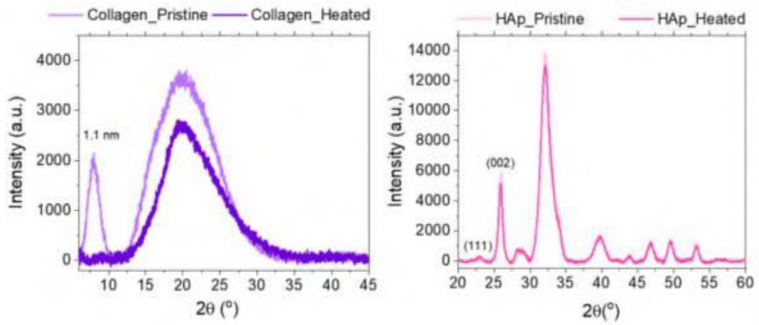

2Θ(°)	(hkl)	FWHM	
		HAP_Pristine	HAP_Heated
23.0	(111)	0.63 ± 0.10	0.72 ± 0.08
25.9	(002)	0.69 ± 0.01	0.69 ± 0.01
28.2	(102)	0.57 ± 0.05	0.58 ± 0.05
29.0	(210)	0.91 ± 0.11	0.99 ± 0.10
32.1	(112)	1.50 ± 0.01	1.44 ± 0.01
33.6	(300)	1.17 ± 0.58	1.50 ± 0.02
39.3	(212)	1.52 ± 0.19	1.31 ± 0.52
40.0	(130)	0.55 ± 0.06	1.31 ± 0.03
43.8	(113)	1.00 ± 0.02	0.57 ± 0.07
46.8	(222)	0.88 ± 0.02	0.98 ± 0.02
49.6	(213)	0.70 ± 0.02	0.85 ± 0.02
53.2	(004)	1.47 ± 0.02	0.73 ± 0.02

Fig 2. XRD spectrum of pristine and heated collagen, pristine and heated HAp and a table listing the location of the diffraction peaks, lattice planes and the FWHM of each peak obtained from pristine and heated HAp extracts.

Pristine collagen shows a diffraction peak at $2\Theta = 8.0^o$, which indicates the distance between

the molecular chains. In contrast, the broad reflection peak between 15° to 30° corresponds to amorphous scatter due to unordered components of collagen fibers.[2] Comparing pristine and heated collagen extract, the lack of diffraction peak at 2Ө = 8.0^o and lower intensity of the broad reflection peak indicates increased disorder within the heated collagen structure. Furthermore, the narrower peak between 15° to 30° suggests that the crystallite size within the heated collagen is larger.

Whereas for the case of extracted HAp, both pristine and heated HAp shows similar diffraction profiles and peaks. However, lower intensities for peaks located at 2Ө = 25.9^o, 40.0^o after heating is detected. The FWHM for heated HAp is also generally larger compared to pristine HAp. The result thus suggests a loss of crystallinity in heated HAp following the release of trapped H_2O and physisorbed CO_2.

Comment 4.1: For the FT-IR results of collagen and HAp before and after the heating, since the absorbance obtained by the FT- IR spectra will be changed according to the amount of the measured sample. Thus, it is unsuitable to directly compare the absorbance before and after the heating, and it is necessary to find the reference band and unify the intensities of the reference peaks. Then, the authors should compare the relative intensities and the reference peak. For Fig. 3a, there is too much noise in the FTIR to see the exact absorption band, and please analyze in smooth the data.

Reply 4.1: *Thank you for the feedback. We have smoothed the FTIR graphs by taking adjacent-average, and the graphs are now normalized to the C-C peak at 1645cm^{-1}.*

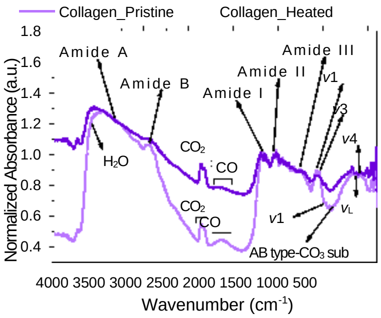

Fig 3. Smoothed FTIR of pristine and heated collagen extract and a table containing peak assignment, peak position and the relative peak intensity of the assigned FTIR peaks with respect to the C-C peak.

Comment 4.2: In the wavenumber between 2000 and 2500 (cm-1) attributed to the carbon dioxide component in the air, it is not suitable to judge using the carbon dioxide component existing in the structure. If you want to evaluate the carbon dioxide component, it is recommended to separately compare the absorption band of the carbonate group.

Reply 4.2: *Thank you for the suggestion. For clarity, the XPS results will be used for any discussions on the presence of CO_2 components.*

Comment 4.3: For the fish scales before and after the heating (Fig. 3c), the absorption bands between wavenumber 600 and 900 show the change that is not discussed, and this part of the HAp structural change is probably linked to the luminescence mechanism. This point is important in the paper and the authors should analyze.

Reply 4.3: *Thank you for the suggestion. We have analysed the peaks between 600 cm^{-1} to 900 cm^{-1} and found that indeed there exist fluorescing contributing peaks located at v_4 (605 cm^{-1}), v_L (635 cm^{-1}) and v_3 (871 cm^{-1}) associated to PO_4 bending, liberation mode of OH and CO_3 group due to presence of AB-type CO_3 substitution.*[3]

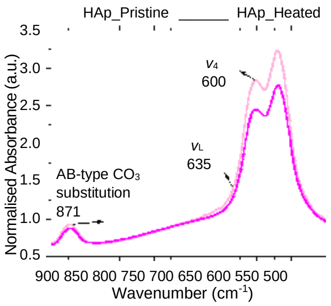

Fig 4. Identification of FTIR peaks between 600 cm^{-1} to 900 cm^{-1}.

Comment 5: Based on the absorption peaks at 1419 and 1457 (cm-1) in Figure 2h, the author assumed that the HAp composition was AB-co-substituted carbonated HAp. However, the peak near 1457 (cm-1) is also present in B- type carbonated HAp and cannot be used to determine the HAp composition. The absorption peak around 1539 (cm-1) should be used to confirm the presence of type A carbonate ions.

Reply 5: *Thank you for highlighting this point. We have re-evaluated the FTIR results within the mentioned range and identified the following peaks attributed to CO_3 A- and B-type, respectively.*[4]

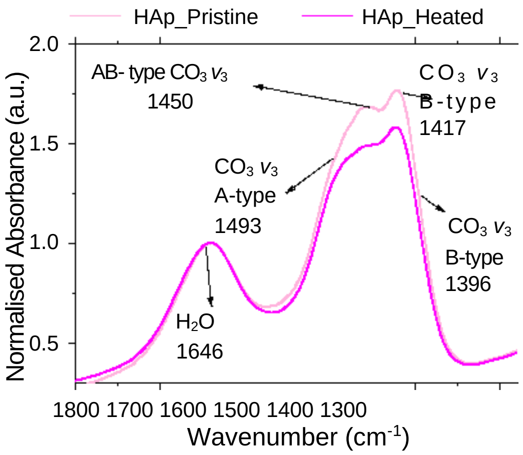

Fig 5. Identification of FTIR peaks between 1300 cm^{-1} to 1800 cm-1.

Comment 6: The authors explained that the fluorescence enhancement is contributed by the dehydrated and defective collagen and HAp. These explanations are lopsided and lack of convince, and the reason is possible due to the special complex chemical structure formed between collagen and HAp. In particular, at near 270°C, the chemical structure of collagen begins to change and then the fluorogenic chemical group of collagen is possibly modified to the surface of HAp and then to form the special composite structure. As a result, the fluorescence would be enhanced. The most significant cause of the enhanced fluorescence is probably derived from collagen rather than HAp. The authors should analyze and discuss the mechanisms.

Reply 6: *Taking into consideration the above suggestion, coupled with a detailed literature review, we would like to propose the following mechanism for the observed thermal-initiated fluorescence:*

> *In this work, collagen and HAp contributed to the enhanced fluorescence observed. Due to the reported short lifetime of UV excited fluorescence chromophores cross-link in the collagen fibers,[5,6] presence of carbonated HAp whose broad PL spectrum closely matches our measured results,[7,8] will have a greater contribution to the observed fluorescence enhancement. As HAp and collagen are still distinguishable after thermal annealing (*Figure 6***b-e***), the most likely interaction region between these organic and inorganic matrices remains only at the interfaces. Rather than forming a new complex, we propose that the denatured collagen likely forms a coating at the interface on the HAp.*

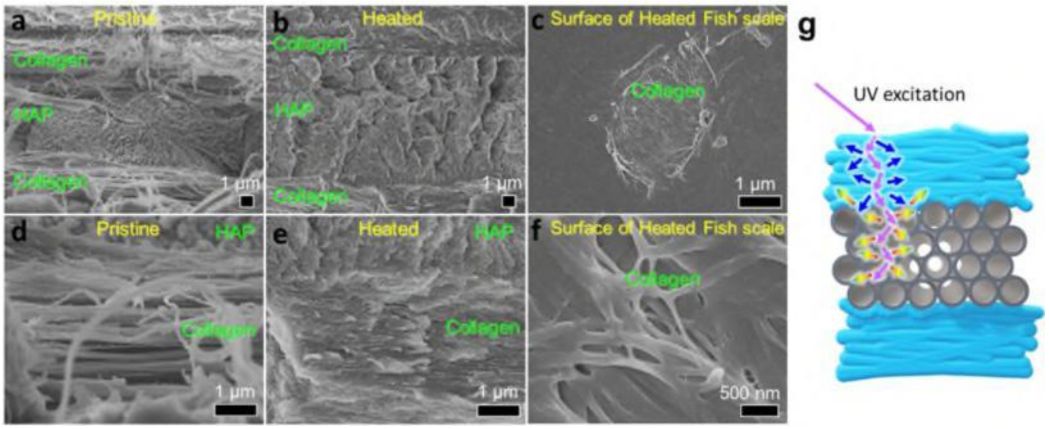

Fig 6. SEM images of (a, d) pristine, (b, e) heated and (c, f) surface of heated fish scale. (a-c) shows a lower magnification of the site of interest, while (d-f) shows a higher magnification of the sites presented in (a-c). (g) Schematic of the proposed excitation process within the denatured collagen/HAp matrix.

> *Collagen fluorescence has been attributed to multiple chromophores cross-links, such as tyrosine-like dityrosine and polytyrosine.[9] When thermal annealed, the process leads to the breakdown of collagen at the thermal labile domain, Gly877 to Pro941.[10] The denaturation of collagen then results in the random formation of tyrosine-like cross-links with longer, uncoupled coiled states confined by surrounding fibers network. Although tyrosine-like cross-links fluorescence mainly between 400nm – 420nm,[9] dehydration can result in the formation of polymer-in-a-box phenomenon[11] such that the formation of elongated polymer chains can cause a redshift in the emission. While these chromophores under UV excitation have been reported to exhibit a short emission lifetime of < 80ns,[6] the collagen's proximity to the porous carbonated HAp can result in secondary excitation of these HAps from photons emitted by de-excited denatured collagen. The observable fusing*

of these collagen fibers upon thermal annealing also provided a less destructive optical path for excitation light transmission towards the porous HAp, where scattering and attenuation of light is minimized without travelling through multiple layers of air/collagen fiber interfaces. The process then allows more photons to interact with the underlying carbonated HAp.

Although the energy gap of HAp was reported to be 5.23eV,[12] considering that the heating temperature of 270 ^oC is much lower than the detected activating temperature of (445 ^oC, Fig. 2c) required for the decomposition of A- and B-type carbonate substitutes, the presence of these carbonate impurities thus played a vital role in the observed fluorescence from pristine and heated HAp. When heated, distortion to the intrinsic HAp structure due to carbonate impurities and release of entrapped H_2O molecules can result in the introduction of more energy levels within the forbidden zone that are responsible for electron-hole recombinations,[7,13-15] resulting in the fluorescence emission not observed from heated pure HAp structures (Supplementary Fig. 7). presence of defects introduced energy levels is evident through the observation of multiple fluorescents under different excitation lights (Supplementary Fig. 8).

Comment 7: The mechanism of luminescence needs the further investigation. Comparing the fluorescence spectra in the paper with those in other studies reveals the differences in the spectral shapes, especially, at the fluorescence wavelength of 410 nm for collagen in the paper, which is around 530 nm in the other studies.

Reply 7: *Menter[16] presented a work on the temperature dependence of collagen fluorescence in which a 325 nm excitation (similar to ours) produces weak fluorescence at 400 nm. This emission's source is attributed to dityrosine, which formed photochemically or enzymatically from neighbouring tyrosine residues.*

In our work, the deconvoluted PL spectrum presented in supplementary Fig. 9, shows a weak fluorescence peak at (390.5 ± 0.6) nm, indicating a possible contribution from tyrosine-like fluorescence chromophores. However, the strongest peak in our PL graphs is at (547.2 ± 0.6) nm. As such, it is unlikely that collagen is the sole contributor to the enhanced fluorescence presented in our work. Furthermore, our PL results exhibit a broad photoluminescence spectrum which is also vastly different from the reported PL spectrum from pure collagen[9,16] and TG results also show a presence of HAp in collagen extract. Thus we proposed a combinatory contribution from denatured collagen and HAp as the mechanism of luminescence in our work (detailed as listed in Reply 6 above).

Comment 8: The fluorescence intensity at 395-420 nm, which causes the blue fluorescence, is lower in the paper. Therefore, it is doubtful whether the fluorescence color can be considered blue.

Reply 8: *Thank you for highlighting this point. The term "blue" is largely used in describing fluorescence from pristine samples, whose fluorescence at 395 nm – 420 nm is more intense*

(Supplementary Fig. 9a-b) in both pristine collagen and HAp extract compared to the heated extracts. To describe the fluorescence observed from heated samples, the term "cyan" is used.

Comment 9: No clear structural changes in collagen and HAp due to the heating were observed. Therefore, the enhancement of fluorescence intensity may not be due to structural changes, but rather the external factors such as an increase in excitation intensity due to impurity removal. Additional measurements should be conducted to further evaluate these external factors.

Reply 9: *SEM images, presented in Fig. 3 of our revised manuscript and Fig. 6 in Reply 6 above, prove that structural changes occurred for both collagen and HAp structures after thermal annealing. As proposed in reply 6 above, chemical and structural changes can contribute to the observed fluorescence enhancement.*

Comment 10: The authors explained that the reason for the increase in phosphate ion content being greater than calcium ion content was due to an increase in the carbonate ion substitution in HAp, but this was not evident in the presented data. In other words, the increase in the content is thought to be due to the abundance ratio of calcium and phosphate ions in the initial state. Therefore, I suggest presenting not only the increase in ion content but also the content of calcium and phosphate ions in the initial state, and then comparing then with the data obtained after the heating.

Reply 10: *Atomic percentage of the phosphate ions and calcium ions before and after thermal annealing are presented in the table below:*

Pristine			***Heated***		
Elements	*Peak Binding Energy (eV)*	*Composition (At%)*	*Elements*	*Peak Binding Energy (eV)*	*Composition (At%)*
P 2p3 (PO4)$^{3-}$	*133.4*	*0.6*	*P 2p3 (PO4)$^{3-}$*	*133.2*	*3.0*
Ca 2p3 Ca^{2+}	*347.4*	*1.2*	*Ca 2p3 Ca^{2+}*	*347.4*	*3.4*

XPS is surface state analysis. That means anything below 8nm cannot be detected. It is likely that during the thermal annealing process, Ca and P ions are released from deeper depths and migrate towards the surface, increasing the atomic percentage detected after annealing.

Comment 11: In Figure 7c, instead of comparing the photos under brightfield illumination, why not compare the photos under UV and green excitation, and discuss the locational sites of fluorescence due to the heating and RhB adsorption?

Reply 11: *Pristine and heated fish scale after undergoing RhB adsorption is presented below. Due to the low concentration of the RhB, there is no clear visualization of RhB's pink fluorescence under UV excitation on both pristine and heated fish scales. Hence we presented a comparison of brightfield illumination and green light excitation to highlight the location of RhB adsorption on the fish scale base on the more distinct orange fluorescence.*

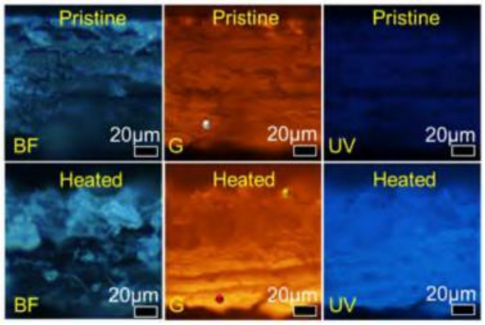

Fig 7. Comparison of pristine and heated fish scale with RhB under BF, Green and UV excitation.

Comment 12: The abstract, introduction and conclusion that correspond to the text contents should be revised. Furthermore, the abstract and conclusions should focus more on what the findings of the study mean rather than describing the study again. For example, the following points should be focused.

Reply 12: *Thank you for your feedback. We have adjusted the focus of our revised manuscript's abstract, introduction and conclusion.*

Comment 12.1: I am having the trouble to understand the novelty of this research. It would be helpful if the introduction emphasized the superiority of this research compared to existing materials for steganography and dye adsorption.

Reply 12.1: *Thank you for your suggestions. We highlight below some of the literature work on steganography and dye adsorption and the significance of our work in these contexts.*

Steganography

<u>*Why steganography?*</u>
Steganography is widely used in enhanced data structures, watermarks, document tracking, file authentication and private communication. Many materials and data processes, such as Raman ink,[17] microdots,[18] character arrangements, and spread spectrum communications[19] have been implemented to date. These processes have advantages such as multiplexing steganography, ease of message transmission and multilevel security. However, they also have drawbacks, such as requiring more sophisticated instruments for message extraction and computer code for deciphering messages, which can be more susceptible to hacking.

<u>*What is so unique about our work?*</u>

With every discovery of a message hidden from an existing application, a new steganographic application is devised. Old methods are given new twists.[20] *As in our case, we propose a new material for such a steganographic application. Fish scale steganography is a new form of steganography that functions in a multidimensional level of hiding messages with the versatility of being both micro and macroscopic, transmitting text and images. Information can easily be spread across different pieces which are compact enough to be transported. Most importantly, it does not require algorithms to minimize the risk of cyber hacking, one of today's most common forms of steganography attacks.*[21] *The widely available versatile laser writing techniques allow this discovery to be readily adapted and customized to the need of its encoder. Our discovery highlights the potential of using natural products as a possible source of steganography material that is already unique. The vast number of natural products naturally increases the degree of challenges faced by intended attacks.*

Rhodemine B Adsorption

Why RhB?
RhB, which can lead to cancer, respiratory diseases, liver failure and dermatitis issues,[22] *can also threaten sea ecosystems due to its wide range of water-related applications, such as flow tracing agents*[23] *and textile dyes.*[22] *Various materials have been proposed as alternative RhB removal adsorbents. Out of these, different biomass types have been studied and compared to our work.*

What is so unique about our work?
Biomass such as activated carbon white sugar can effectively remove 98% of RhB within 12mins of contact time.[24] *However, the functionalization process is very energy and time-intensive, involving multiple steps from chemical treatment, hours of high temperature (700°C) thermal annealing in a controlled environment to multiple rounds of repeated washing and drying processes.*

In comparison, our functionalized fish scale created using a single thermal annealing step at 270 ^{o}C in ambient for 3mins can effectively remove 91% of RhB within a contact time of 10 mins. Our reported work is thus more energy-efficient, cost and time-saving. Not only so, compared to other biomass listed in reference 25-31, an average contact time for biomass removal of RhB with removal percentage ranging between 85-95% is determined to be approximately 100 mins.[25-31] *This is 10 times slower than our work. Furthermore, while fish scale has been reported as a biosorbent for heavy metals and other dyes,*[32] *this is the first time fish scale is being reported as a bio-sorbent for Rhodamine B, and it is even more effective than half of the reported biomass RhB sorbents shown in reference 25-31.*

12.2: I am unsure why you are aiming to apply your research to two very different applications, steganography materials and dye adsorbents. It would be beneficial if the introduction explained the advantages of having a material that can serve both purposes, after listing the necessary properties for each application.

Reply 12.2: *Looking at a future of scarce resources, we feel strongly that materials with multiple functionalities and applicability will be highly desirable. Take carbon nanotubes, for example. It is not only a highly flexible material, its good electrical conductivity, unique aspect ratio, and super-hydrophobicity have allowed it to develop into a carbon-based electronic circuit, field emitting electron source and self-cleaning coat.*

In the manuscript presented, we intend to show that fish scale waste is so versatile that it is not just a collagen or HAp source. In a single process (controlled thermal annealing), we successfully functionalize a waste, turning it into one with dual applications (RhB adsorption and Steganography). Just like the saying goes – Two for the price of One.

Not only so, the steganographic application aspect can also be seen as an alternative use for this end-of-life, thermally functionalized fish scale adsorbent. This manuscript has established that these thermally functionalized fish scales RhB adsorbent can be recovered and reused through water-assisted ultra-sonication. However, practically, there is bound to be a point where the efficiency of these functionalized fish scales at adsorbing RhB pollutants is no longer worth the time and effort required for the recovery process. Instead of tossing this end-of-life bio-adsorbent away, controlled RhB adsorption-induced changes to observable fluorescence visible only under green excitation allow the fish scale bio-sorbent to find alternative use in steganography due to their good long-term stability.

– – – – – –

Reviewer #3 (Remarks to the Author):

In the opinion of this reviewer, manuscripts that aim to be published in top journals need to contain highly original research in groundbreaking fields, that hold promise for exceptional developments and significant advance of science. As already stated in my first round of review this manuscript does not meet any of these criteria. Annealing of organic materials to obtain luminescent materials is already known and well described, as also confirmed by the authors in reply to the request by the previous reviewers to explain the origin of luminescence. The proposed applications as detectors and adsorbents for RhB do not represent a groundbreaking application. Finally, the hidden messages, the patterns and tha applications in ciphering seem like scarcely significant both from a scientific as well as from an artistic or applicative point of view. I do not recommend publication in Nature Communications.

Reply: *Thank you very much for your comments.*

In our humble opinion, the main value of this work is to draw the researcher's attention to an overlooked resource. Namely, nature-based materials, usually regarded as waste, may offer unexpected functionalities with further engineering processes, and the modified functional materials demonstrated multi-faceted applications. Fish scales are abundant, yet the discoveries made in this work have not been reported before. Their modified fluorescence property is utilized for display, encryption and messaging. Their modified absorption capability is utilized as an active medium for extracting toxic chemicals from contaminated water.

As we are thinking of building a more sustainable environment, it is certainly encouraging to be able to transform seemingly useless waste into useful mediums that display multi-faceted

applications. Motivated by our discovery of such attributes in fish scale, students in our research group are now looking into similar studies on rice husks, seeds, seashells etc. Hence we hope this work can motivate other interested researchers to conduct similar studies.

There have been reports on fish scales as alternative chemical sorption material, collagen sources for medical applications, and even creating carbon nanodots from these fish scales. Still, the novelty of our work stands as follows:

1. *Undiscovered optical properties in the fluorescent regime which is different from the reflection/structure of fish scales that have been previously studied for application as an invisible cloak.*
2. *We look at using an entire fish scale without going through tedious and costly post-processing methods reported in fish scale waste studies. The simplicity, safety and ease of functionalizing the fish scale waste facilities the transition from lab base research to industrial applications without having the industries bear the cost of developing new fabrication lines, often one of the main deterrents for industrial uptake of lab-base research work.*
3. *Compared to other biomass listed in reference 25-31, an average contact time for biomass removal of RhB with removal percentage ranging between 85-95% is determined to be approximately 100 mins.[25-31] This is 10 times slower than our work, and the functionalized fish scale presented in our work is even more effective than half of the reported biomass RhB sorbents shown in reference 25-31.*
4. *While fish scale has been reported as a biosorbent for heavy metals and other dyes,[32] this is the first time fish scale is being reported as a bio-sorbent for Rhodamine B.*

As science communicators, we pay attention to how our work is presented to our readers. Hence we put effort into creating appealing artistic displays utilizing our control over the modified fish scale. We believe that merging science with art is an effective means of science communication. Through the painting and sculpture presented at the end of the manuscript, the ciphering application of this work is effortlessly showcased to readers who may or may not be in the field of ciphering. We are very encouraged by the same reviewer's comment in the first round of review when the reviewer commented that the resultant artistic images are "appealing".

Reference

1 Li, J. *et al.* The investigation of thermal decomposition pathways of phenylalanine and tyrosine by TG-FTIR. *Thermochimica Acta* **467**, 20-29 (2008). https://doi.org:10.1016/j.tca.2007.10.014

2 Meng, Z. J. *et al.* Dissolution and regeneration of collagen fibers using ionic liquid. *International Journal of Biological Macromolecules* **51**, 440-448 (2012). https://doi.org:10.1016/j.ijbiomac.2012.05.030

3 Kashkarov, V. M. *et al.* X-ray diffraction and IR spectroscopy investigation of synthesized and biogenic nanocrystalline hydroxyapatite. *Journal of Surface Investigation* **5**, 1162-1167 (2011). https://doi.org:10.1134/s1027451011120068

4 Madupalli, H., Pavan, B. & Tecklenburg, M. M. J. Carbonate substitution in the mineral component of bone: Discriminating the structural changes, simultaneously imposed by carbonate in A and B sites of apatite. *Journal of Solid State Chemistry* **255**, 27-35 (2017). https://doi.org:10.1016/j.jssc.2017.07.025

5 Chung, R. J. Study of Hydroxyapatite Nano Composites with Photoluminescence Properties. *Biomedical Engineering-Applications Basis Communications* **23**, 107-112 (2011). https://doi.org:10.4015/s1016237211002451

6 Yova, D., Hovhannisyan, V. & Theodossiou, T. Photochemical effects and hypericin photosensitized processes in collagen. *Journal of Biomedical Optics* **6**, 52-57 (2001). https://doi.org:10.1117/1.1331559

7 Machado, T. R. *et al.* Designing biocompatible and multicolor fluorescent hydroxyapatite nanoparticles for cell-imaging applications. *Materials Today Chemistry* **14** (2019). https://doi.org:10.1016/j.mtchem.2019.100211

8 Gonzalez, G. *et al.* Effect of carbonates on hydroxyapatite self-activated photoluminescence response. *Journal of Luminescence* **195**, 385-395 (2018). https://doi.org:10.1016/j.jlumin.2017.11.058

9 Theodossiou, T. *et al.* Thermally induced irreversible conformational changes in collagen probed by optical second harmonic generation and laser-induced fluorescence. *Lasers in Medical Science* **17**, 34-41 (2002). https://doi.org:10.1007/s10103-002-8264-7

10 Miles, C. A., Burjanadze, T. V. & Bailey, A. J. The kinetics of the thermal-denaturation of collagen in unrestrained rat tail tendon determined by differential scanning calorimetry. *Journal of Molecular Biology* **245**, 437-446 (1995). https://doi.org:10.1006/jmbi.1994.0035

11 Miles, C. A. & Ghelashvili, M. Polymer-in-a-box mechanism for the thermal stabilization of collagen molecules in fibers. *Biophysical Journal* **76**, 3243-3252 (1999). https://doi.org:10.1016/s0006-3495(99)77476-x

12 Avakyan, L. A. *et al.* Optoelectronics and defect levels in hydroxyapatite by first-principles. *Journal of Chemical Physics* **148** (2018). https://doi.org:10.1063/1.5025329

13 Aronov, D. *et al.* Electronic states spectroscopy of Hydroxyapatite ceramics. *Journal of Materials Science-Materials in Medicine* **18**, 865-870 (2007). https://doi.org:10.1007/s10856-006-0080-3

14 Bystrov, V. S. *et al.* Computational study of hydroxyapatite structures, properties and defects. *Journal of Physics D-Applied Physics* **48** (2015). https://doi.org:10.1088/0022-3727/48/19/195302

15 Matsunaga, K. & Kuwabara, A. First-principles study of vacancy formation in hydroxyapatite. *Physical Review B* **75** (2007). https://doi.org:10.1103/PhysRevB.75.014102

16 Menter, J. M. Temperature dependence of collagen fluorescence. *Photochemical & Photobiological Sciences* **5**, 403-410 (2006). https://doi.org:10.1039/b516429j

17 Gu, Y. Q., He, C., Liu, F. G. & Ye, J. Raman Ink for Steganography. *Advanced Optical Materials* **9** (2021). https://doi.org:10.1002/adom.202002038

18 Clelland, C. T., Risca, V. & Bancroft, C. Hiding messages in DNA microdots. *Nature* **399**, 533-534 (1999). https://doi.org:10.1038/21092
19 Bharti, S. S., Gupta, M. & Agarwal, S. A novel approach for audio steganography by processing of amplitudes and signs of secret audio separately. *Multimedia Tools and Applications* **78**, 23179-23201 (2019). https://doi.org:10.1007/s11042-019-7630-4
20 Johnson, N. F. & Jajodia, S. Exploring steganography: Seeing the unseen. *Computer* **31**, 26-+ (1998). https://doi.org:10.1109/mc.1998.4655281
21 Kaufmann, M. *Disappearing Cryptography*. Third Edition edn, 13 (2009).
22 Al-Gheethi, A. A. *et al.* Sustainable approaches for removing Rhodamine B dye using agricultural waste adsorbents: A review. *Chemosphere* **287** (2022). https://doi.org:10.1016/j.chemosphere.2021.132080
23 Skjolding, L. M. *et al.* Assessing the aquatic toxicity and environmental safety of tracer compounds Rhodamine B and Rhodamine WT. *Water Research* **197** (2021). https://doi.org:10.1016/j.watres.2021.117109
24 Xiao, W. *et al.* Preparation and evaluation of an effective activated carbon from white sugar for the adsorption of rhodamine B dye. *Journal of Cleaner Production* **253** (2020). https://doi.org:10.1016/j.jclepro.2020.119989
25 Bello, O. S. *et al.* Rhodamine B dye sequestration using Gmelina aborea leaf powder. *Heliyon* **6** (2020). https://doi.org:10.1016/j.heliyon.2019.e02872
26 Bello, O. S., Adegoke, K. A., Sarumi, O. O. & Lameed, O. S. Functionalized locust bean pod (Parkia biglobosa) activated carbon for Rhodamine B dye removal. *Heliyon* **5** (2019). https://doi.org:10.1016/j.heliyon.2019.e02323
27 Chieng, H. I., Lim, L. B. L. & Priyantha, N. Sorption characteristics of peat from Brunei Darussalam for the removal of rhodamine B dye from aqueous solution: adsorption isotherms, thermodynamics, kinetics and regeneration studies. *Desalination and Water Treatment* **55**, 664-677 (2015). https://doi.org:10.1080/19443994.2014.919609
28 Vijayakumar, G., Tamilarasan, R. & Dharmendirakumar, M. Adsorption, Kinetic, Equilibrium and Thermodynamic studies on the removal of basic dye Rhodamine-B from aqueous solution by the use of natural adsorbent perlite. *J. Mater. Environ. Sci.* **3** (2012). https://doi.org:https:// doi.org/10.9734/ajocs/2020/v7i119013.
29 Saigl, Z. M. & Ahmed, A. M. Separation of Rhodamine B Dye from Aqueous Media Using Natural Pomegranate Peels. *Indonesian Journal of Chemistry* **21**, 212-224 (2021). https://doi.org:10.22146/ijc.58592
30 Inyinbor, A. A., Adekola, F. A. & Olatunji, G. A. Kinetics, isotherms and thermodynamic modeling of liquid phase adsorption of Rhodamine B dye onto Raphia hookerie fruit epicarp. *Water Resources and Industry* **15**, 14-27 (2016). https://doi.org:10.1016/j.wri.2016.06.001
31 Vigneshwaran, S., Sirajudheen, P., Karthikeyan, P. & Meenakshi, S. Fabrication of sulfur-doped biochar derived from tapioca peel waste with superior adsorption performance for the removal of Malachite green and Rhodamine B dyes. *Surfaces and Interfaces* **23** (2021). https://doi.org:10.1016/j.surfin.2020.100920
32 Ighalo, J. O. & Eletta, O. A. A. Recent advances in the biosorption of pollutants by fish scales: a mini-review. *Chemical Engineering Communications* **208**, 1301-1312 (2021). https://doi.org:10.1080/00986445.2020.1771322

REVIEWERS' COMMENTS

Reviewer #2 (Remarks to the Author):

In the revised manuscript, the authors addressed the reviewer's comments well. Therefore, it is recommended to the journal at the present version without the further revision.

Manuscript Submitted to "Nature Communications"

Reference number for this paper is: NCOMMS-22-50918B

"Upcycling Fish Scales Through Heating for Steganography and Rhodamine B Adsorption Application"

Thank you very much for communicating the reviewer's comments on our submitted manuscript. Please find our response to the reviewer's comments below:

REVIEWER COMMENTS

Reviewer #2 (Remarks to the Author):

In the revised manuscript, the authors addressed the reviewer's comments well. Therefore, it is recommended to the journal at the present version without the further revision.

Reply 1: *Thank you for recommending our revised manuscript for publication in the journal without further revision.*

We appreciate the time and effort the editor and reviewers put in on our submitted manuscript. Thank you very much!

Sincerely,

Sharon Xiaodai Lim
