# Supplementary material for: Upcycling fish scales through heating for steganography and Rhodamine B adsorption application
Source: Nat Commun. 2023 Oct 16;14:6508. doi: 10.1038/s41467-023-42080-1 (PMC10579236; doi:10.1038/s41467-023-42080-1)
Supplement: Supplementary file 2 — Peer Review File [file 41467_2023_42080_MOESM2_ESM.pdf]

Supplementary Information

Upcycling Fish Scales Through Heating for Steganography and Rhodamine B Adsorption Application

Malcolm Miao Geng Sow[1], Zheng Zhang[2], Chorng-Haur Sow[3*], Sharon Xiaodai Lim[3*]

[1] NUS High School of Mathematics and Science, 20 Clementi Avenue 1, Singapore 129957

[2] Institute of Materials Research and Engineering (IMRE), Agency for Science, Technology and Research (A*STAR) , 2 Fusionopolis Way, Innovis #08-03, Singapore 138634, Singapore

[3] Department of Physics, National University of Singapore, 2 Science Drive 3, Singapore 117542

*Address correspondence to Dr. Sharon Lim Xiaodai. E-mail: phylimx@nus.edu.sg and Prof. Chorng Haur Sow. E-mail: physowch@nus.edu.sg.

Content

Supplementary Fig. 1. Systematic temperature study of heat-induced enhancement of fluorescence in fish scale.

Supplementary Fig. 2. A systematic study on the effect of heating duration at a constant temperature of 270 °C on the enhancement of fluorescence in fish scale.

Supplementary Fig. 3. Thermal induced fluorescence in fish scale from different fish types.

Supplementary Fig. 4. Specific regions of interest of a single primitive fish scale.

Supplementary Fig. 5. FM images of pristine and heated NaCl to prove that NaCl does not contribute to the fluorescence observed in heated HAp and collagen extracts.

Supplementary Fig. 6. TG curve from the pristine fish scale.

Supplementary Fig. 7. Purity of collagen and HAp extract from fish scales.

Supplementary Fig. 8. The role of defects in the observed fluorescence in extracted HAp from fish scales.

Supplementary Fig. 9. Multicolour fluorescent of heated HAp extract under different excitation lights.

Supplementary Fig. 10. PL comparisons between pristine and heated, collagen and HAp extracts from fish scales. Comparisons are also made between primitive and heated fish scales and the heated extracts.

Supplementary Fig. 11. Systematic study of laser-treated fish scale with different laser intensity and patterning speeds at different focal planes.

Supplementary Fig. 12. Stability study of laser-initiated fluorescence enhancement of fish scale.

Supplementary Fig. 13. SEM images of the cross-section of a fish scale after treatment with low laser power density (1.27 MW cm^{-2}) and at different patterning speeds.

Supplementary Fig. 14. FM images of a 10^{-8} M droplet of RhB placed on a silicon wafer under different excitation lights.

Supplementary Fig. 15. Systematic FM and PL analyses of RhB adsorption efficiency from fish scale heated at different temperatures.

Supplementary Fig. 16. Adsorption and recovery of heated fish scale from MB adsorption.

Table 1. Comparison of the FWHM of the XRD peaks from pristine and heated HAp extracts.

Systematic temperature study of heat-induced enhancement of fluorescence in fish scale.

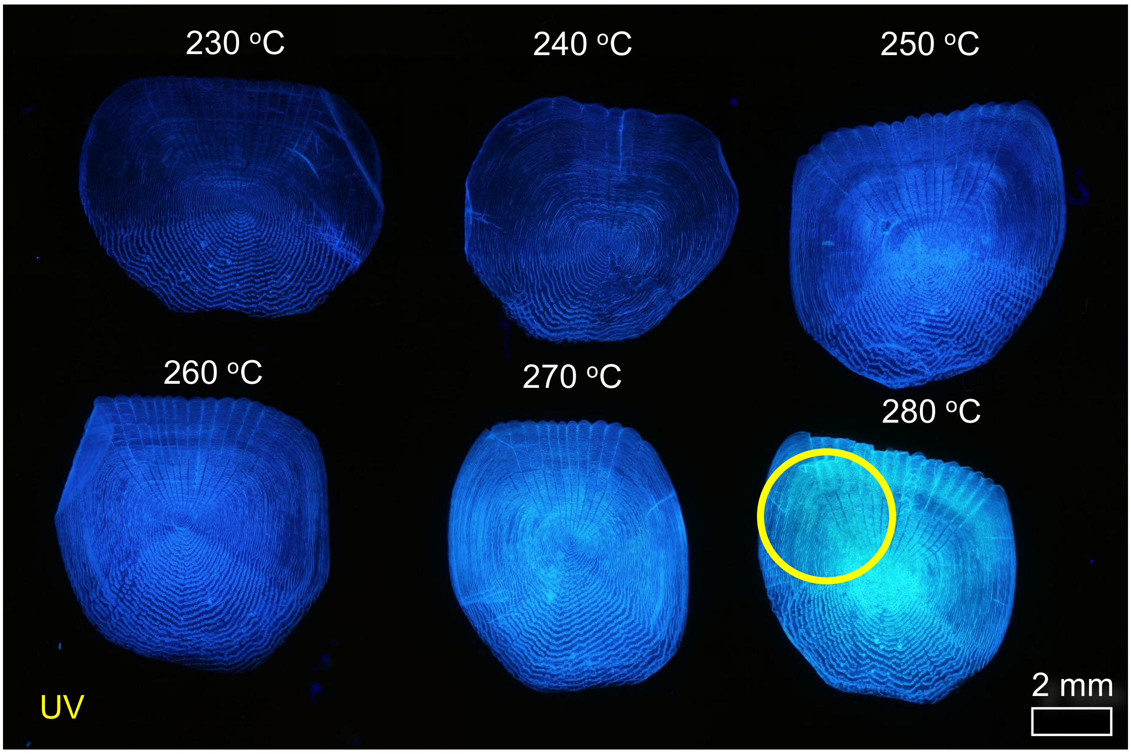

Supplementary Fig. 1. Systematic temperature study of heat-induced fluorescence enhancement in fish scale. The exposure time of this image is intentionally reduced to show the loss of fluorescence enhancement due to charring (enclosed in the yellow circle) when heated at 280 °C.

A systematic study on the effect of heating duration at a constant temperature of 270 °C on the enhancement of fluorescence in fish scale.

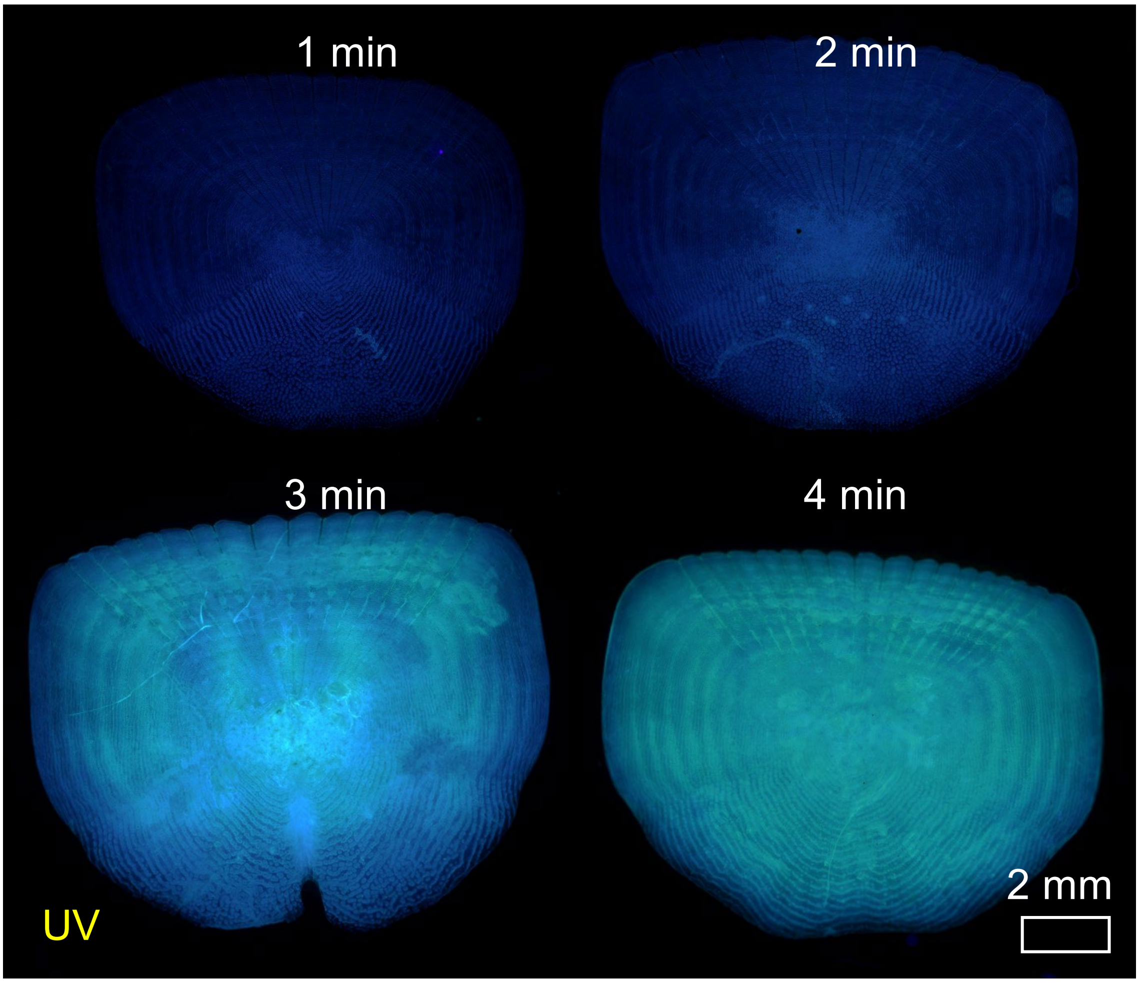

Supplementary Fig. 2. A systematic study on the effect of heating duration at a constant temperature of 270 °C on fluorescence enhancement in fish scale. Enhancement of the fish scale's fluorescence without charring the fish scale was determined to be the most intense for a heating duration of 3 minutes. At 4 minutes, charring of the fish scale resulted in a slight reduction in the fluorescence intensity, like Supplementary Fig. 1.

Thermal induced fluorescence in fish scale from different fish types.

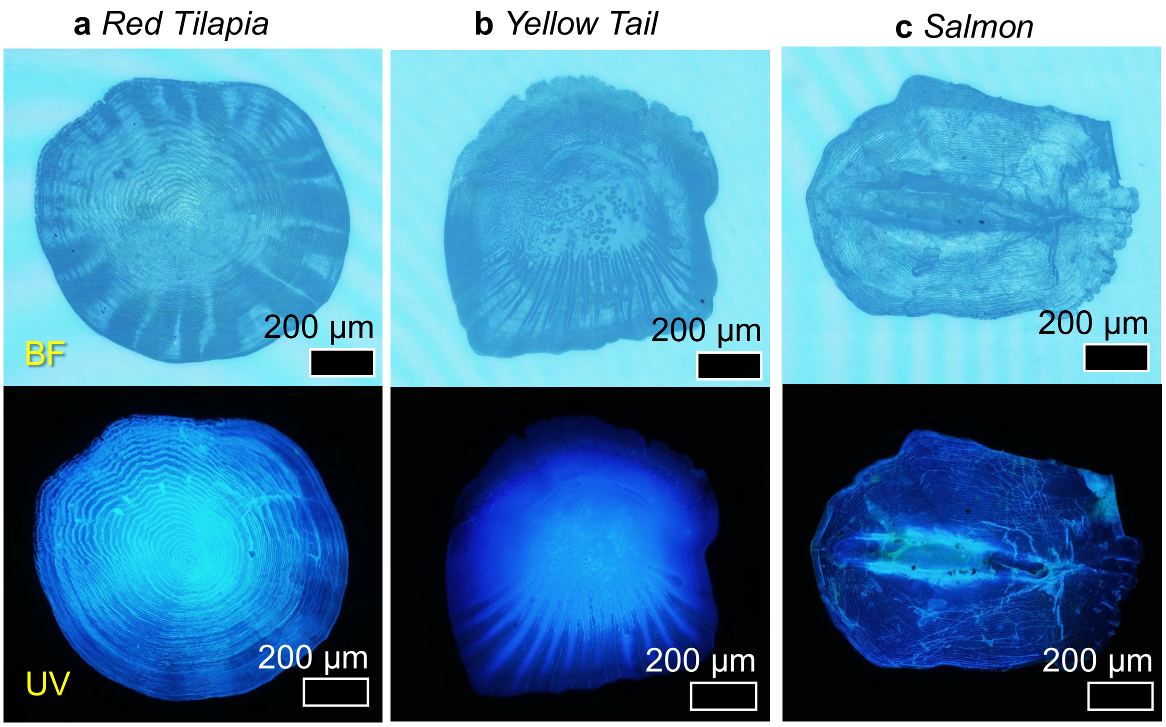

Supplementary Fig. 3. **a** *Red Tilapia*, **b** *Yellow Tail* and **c** *Salmon* scales. Enhanced fluorescence is also observed on heated fish scales (270 °C, 3 minutes) from *Salmon* and *Yellow Tail* (Supplementary Fig. 3). However, the fluorescence enhancement lacks uniformity, which can be resolved by optimizing the heating temperature and duration for different fish scale types.

Specific regions of interest of a single primitive fish scale.

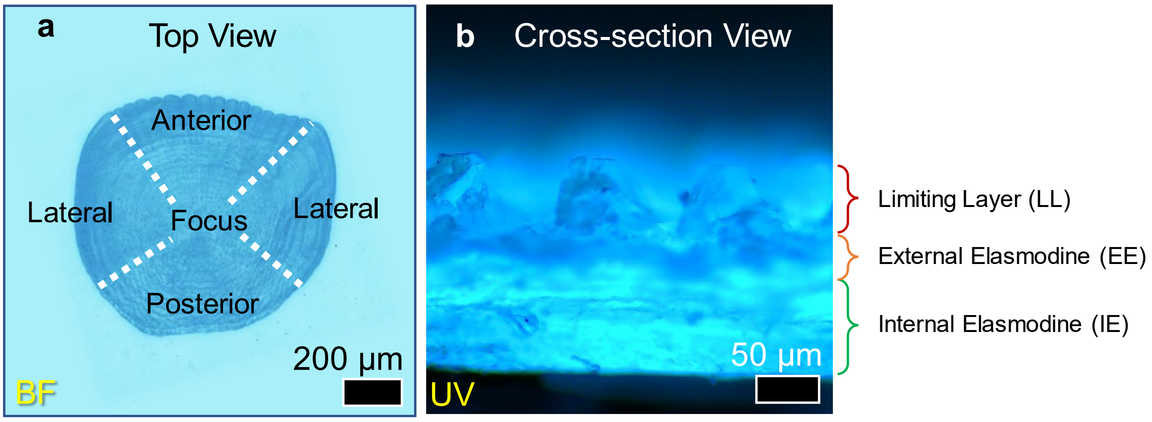

Supplementary Fig. 4. **a** Top and **b** Cross-section of the fish scale.

Generally, a piece of the fish scale (top view, Supplementary Fig. 4a) comprises different regions - the Anterior, the Lateral, the Focus, and the Posterior. Through cross-section imaging, the top layer (in contact with the fish's living environment) is the limiting layer (LL). Below the LL is the elasmodine, which comprises discrete piles of unidirectionally aligned collagen fibers. This elasmodine is further divided into two layers, the external elasmodine (EE) and the internal elasmodine (IE). The subtle distinction between these two layers lies in the latter having lower mineral content (Supplementary Fig. 4b).

FM images of pristine and heated NaCl to prove that NaCl does not contribute to the fluorescence observed in heated HAp and collagen extracts.

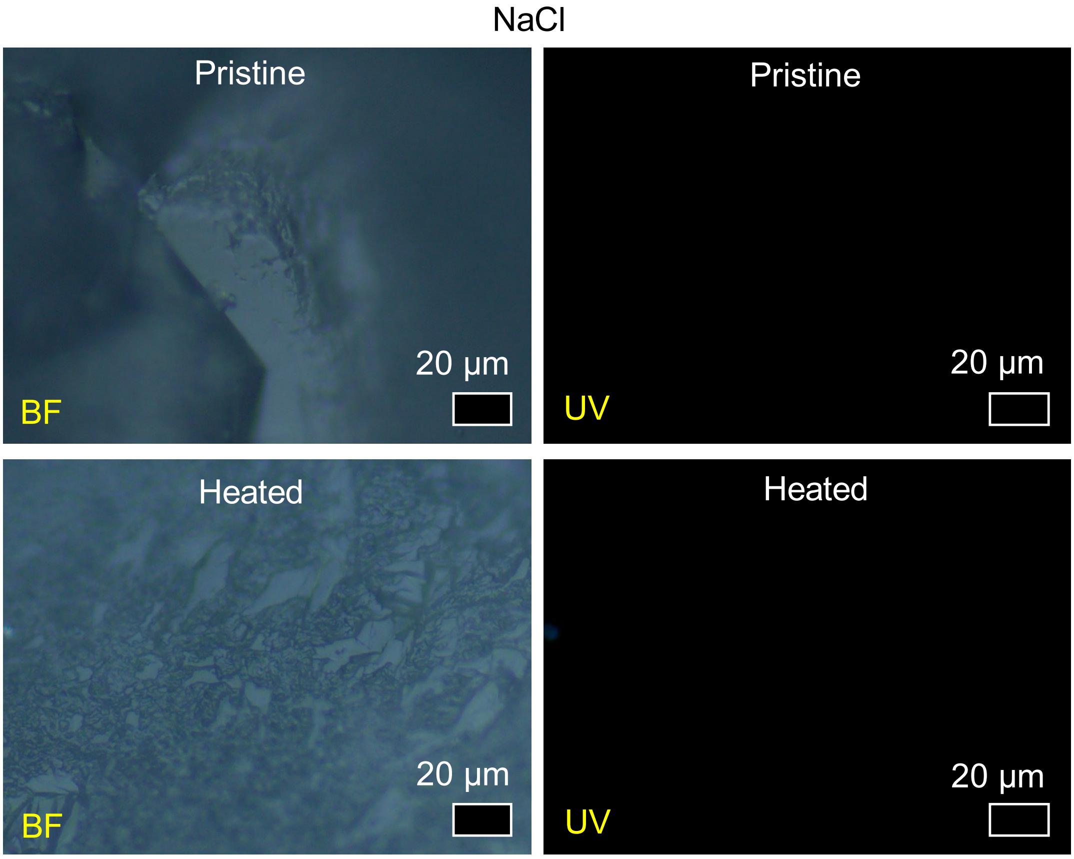

Supplementary Fig. 5. FM images of pristine and heated NaCl observed under BF and UV excitation.

The possibility of a fluorescence contribution from NaCl formed during extraction is eliminated with the lack of fluorescence observed from pristine and heated NaCl samples.

TG curve from the pristine fish scale.

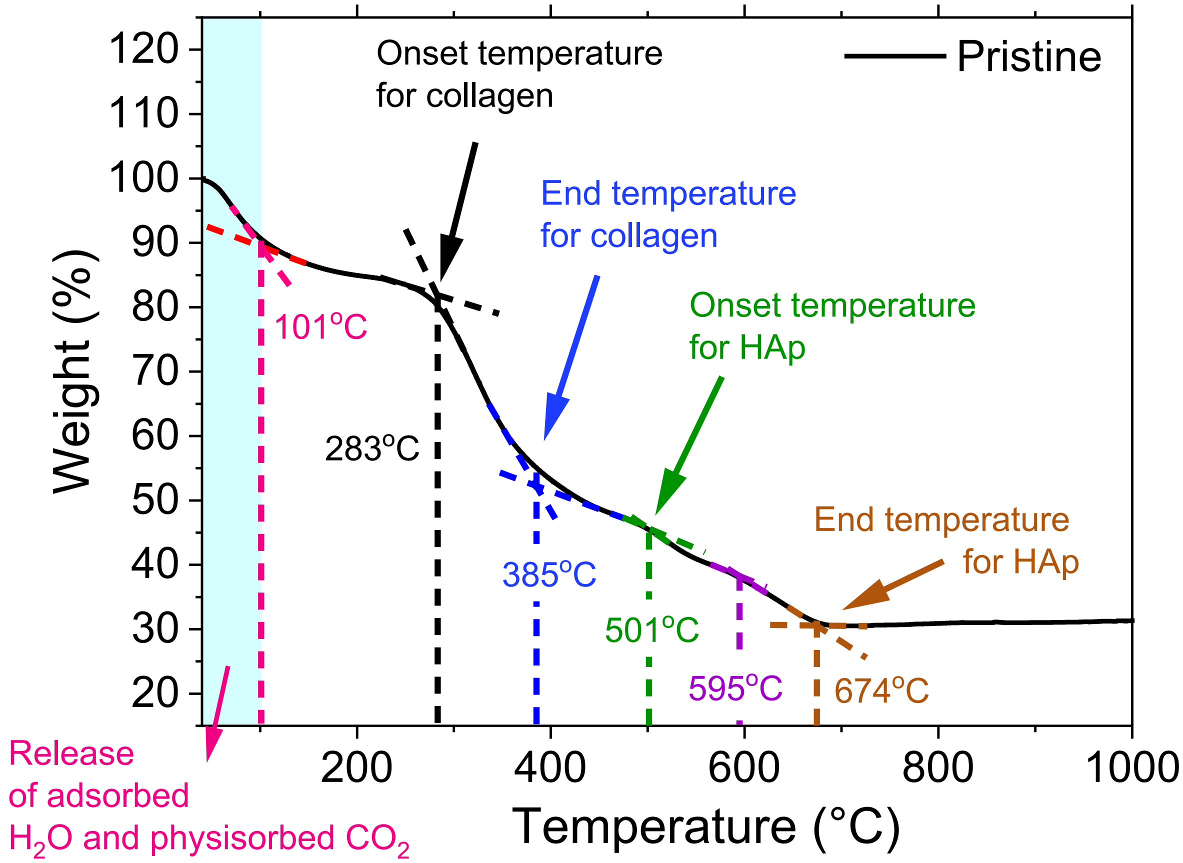

Supplementary Fig. 6. The loss of mass, onset and end temperature corresponding to changes to the collagen and HAp structures are as indicated.

TG curve of the pristine primitive fish scale (Supplementary Fig. 6) shows predominately the release of adsorbed H_2O, physisorbed CO_2, and degradation of collagen and HAp, which agrees with TG curves from the separate entities of collagen and HAp (Fig. 2b-c).

Purity of collagen and HAp extract from fish scales.

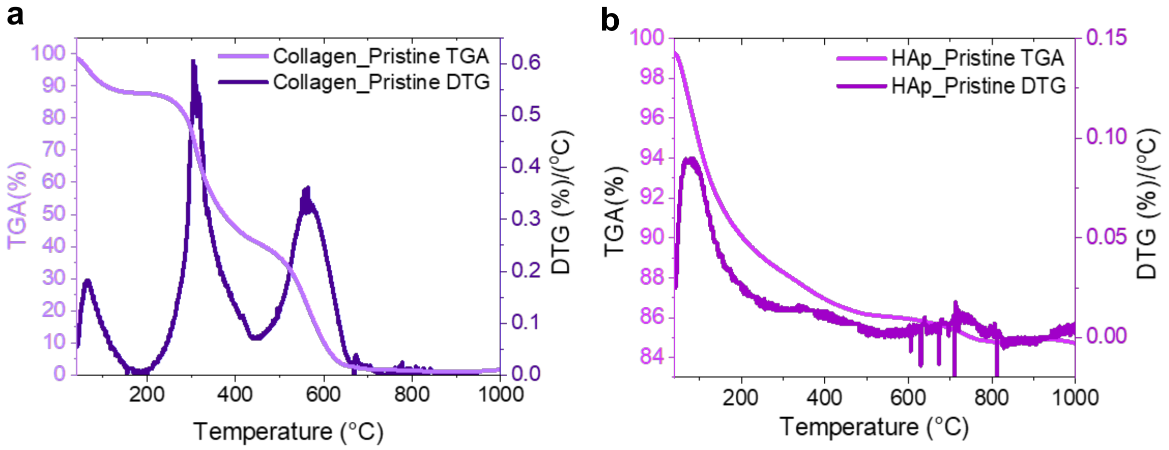

Supplementary Fig. 7. TG and DTG representation of **a** collagen and **b** HAp extracted from fish scales.

Derivative thermogravimetric (DTG) profiles of extracted collagen and HAp are presented in Supplementary Fig. 7 to better represent the changes in the wt % to temperature.

Comparison of the FWHM of the XRD peaks from pristine and heated Hap extracts.

2Θ(°)	(hkl)	FWHM	
		HAp_Pristine	HAp_Heated
23.0	(111)	0.63 ± 0.10	0.72 ± 0.08
25.9	(002)	0.69 ± 0.01	0.69 ± 0.01
28.2	(102)	0.57 ± 0.05	0.58 ± 0.05
29.0	(210)	0.91 ± 0.11	0.99 ± 0.10
32.1	(112)	1.50 ± 0.01	1.44 ± 0.01
33.6	(300)	1.17 ± 0.58	1.50 ± 0.02
39.3	(212)	1.52 ± 0.19	1.31 ± 0.52
40.0	(130)	0.55 ± 0.06	1.31 ± 0.03
43.8	(113)	1.00 ± 0.02	0.57 ± 0.07
46.8	(222)	0.88 ± 0.02	0.98 ± 0.02
49.6	(213)	0.70 ± 0.02	0.85 ± 0.02
53.2	(004)	1.47 ± 0.02	0.73 ± 0.02

Table 1. The table includes the location of the diffraction peaks, lattice planes and the FWHM of each peak from pristine and heated HAp extracts.

The role of defects in the observed fluorescence in extracted HAp from fish scales.

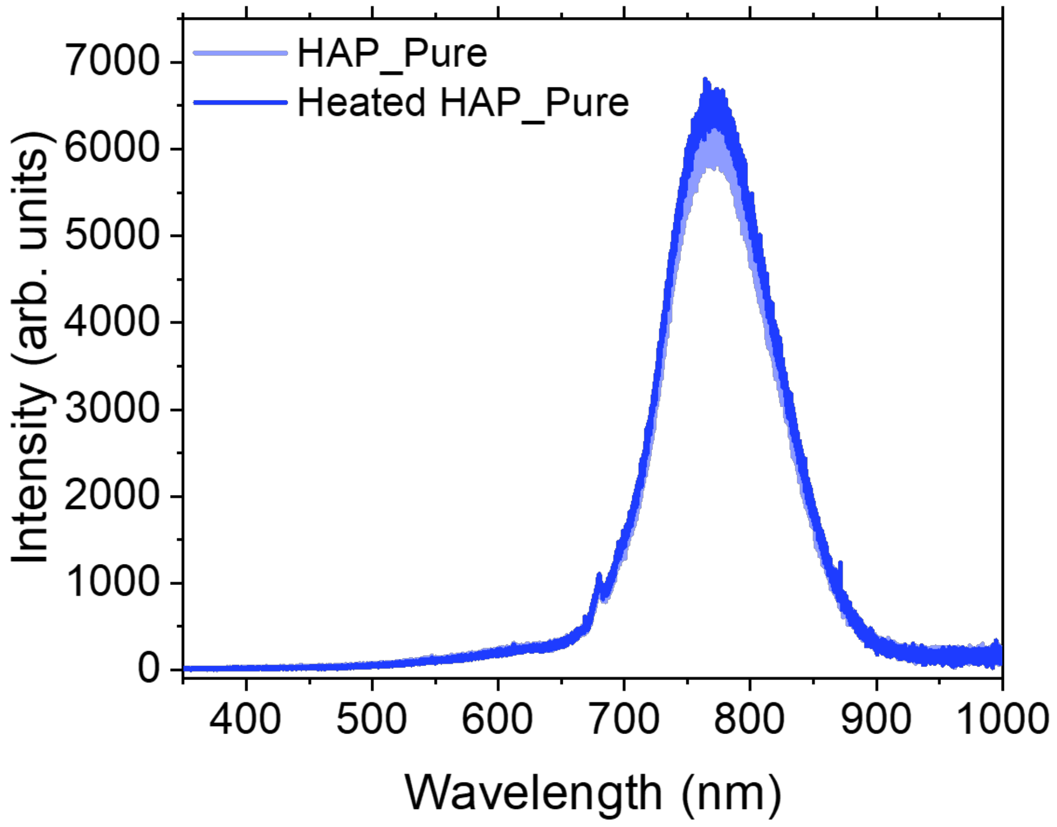

Supplementary Fig. 8. PL spectrum of pure HAp, pristine versus heated.

Multicolour fluorescent of heated HAp extract under different excitation lights.

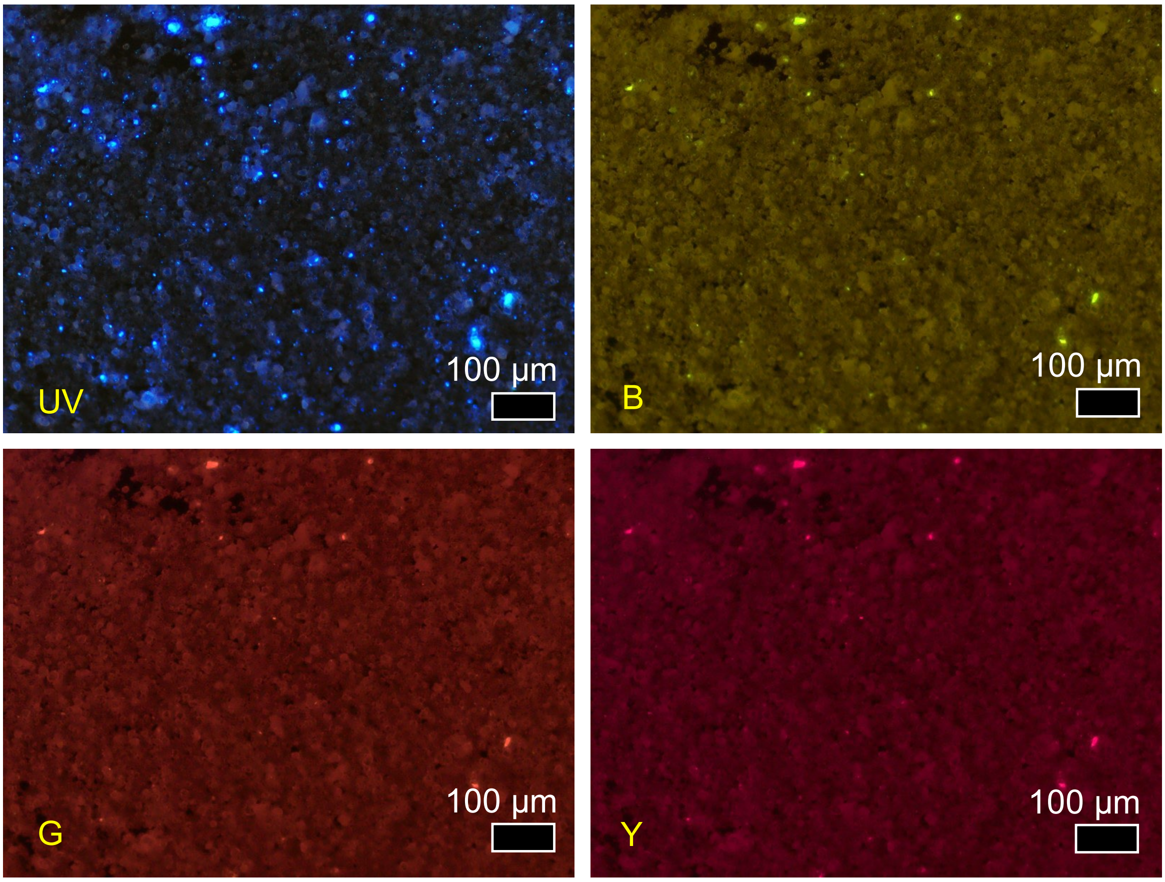

Supplementary Fig. 9. Presence of defects introduced energy levels is evident through the observation of multiple fluorescents under different excitation lights, UV, blue (450-500 nm), green and yellow (530-580 nm) excitation light.

PL comparisons between pristine and heated, collagen and HAp extracts from fish scales. Comparisons are also made between primitive and heated fish scales and the heated extracts.

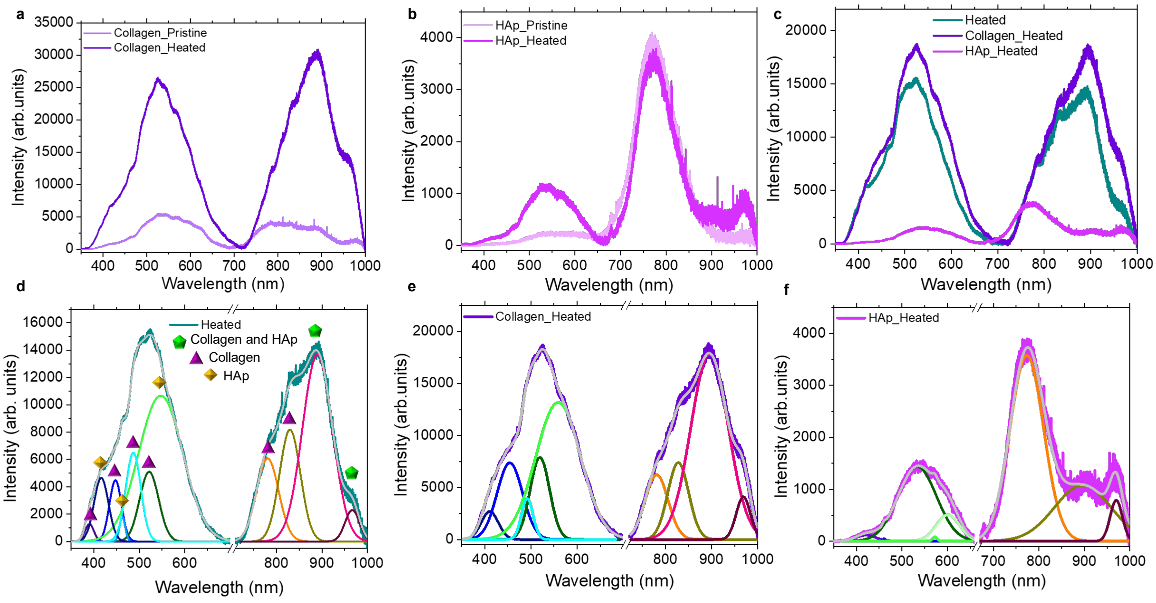

Supplementary Fig. 10. Quantitative comparison (PL) of biofluorescence enhancement between heated and pristine **a** collage, **b** HAp and **c** primitive fish scale. **d-e** Deconvoluted PL spectra from heated **d** primitive fish scale, **e** collagen and **f** HAp.

The fluorescence enhancement from the heated extracts is quantified through PL spectrum analyses with heated collagen showing a 5-fold and 10-fold increase in PL for both visible light (VL) peaks and near-infrared (NIR) ranges (Supplementary Fig. 10a). In contrast, heated HAp has a 6-fold enhancement in the VL range while experiencing 1-fold reduction in intensity in the NIR range (Supplementary Fig. 10b). With some HAp found remaining within the collagen matrix after the extraction process, it is thus reasonable that fluorescence from heated collagen extract will exhibit stronger fluorescence compared to heated HAp (Supplementary Fig. 10c).

The global PL of the heated fish scale is fitted to eleven Gaussian curves (Supplementary Fig. 10d). The fitted results suggest that the observed fluorescence comprises two main peaks at cyan and infra-red ranges, at (547.2 ± 0.6) nm and (890.1 ± 0.2) nm, respectively, and nine smaller peaks located at (390.5 ± 0.6) nm, (416.3 ± 0.8) nm, (447.6 ± 0.7) nm, (462.8 ± 0.1) nm, (486.9 ± 0.3) nm, (521.6 ± 0.7) nm, (780.7 ± 0.4) nm, (829.8 ± 0.2) nm and (966.5 ± 0.1) nm. The main contributors to the individual deconvoluted peaks in the heated fish scale are determined by comparing the deconvoluted graphs from both heated collagen (Supplementary Fig. 10e) and heated HAp (Supplementary Fig. 10f) extracts and indicated in Supplementary Fig. 10d.

Systematic study of laser-treated fish scale with different laser intensity and patterning speeds at different focal planes.

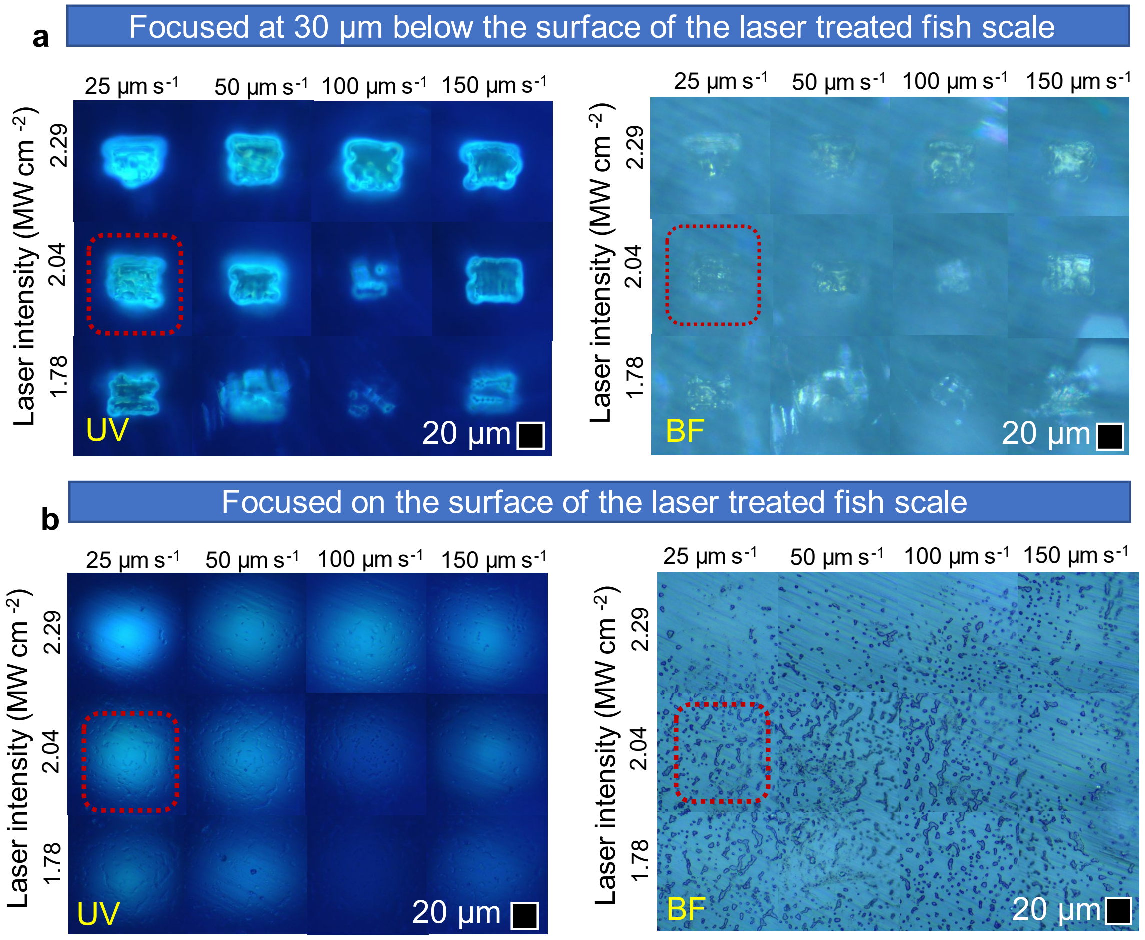

Supplementary Fig. 11. UV and BF imaging of laser-treated fish scale with different laser intensity and patterning speeds taken at **a** 30 μm below the surface of the fish scale and **b** at the surface of the fish scale.

Stability study of laser-initiated fluorescence enhancement of fish scale.

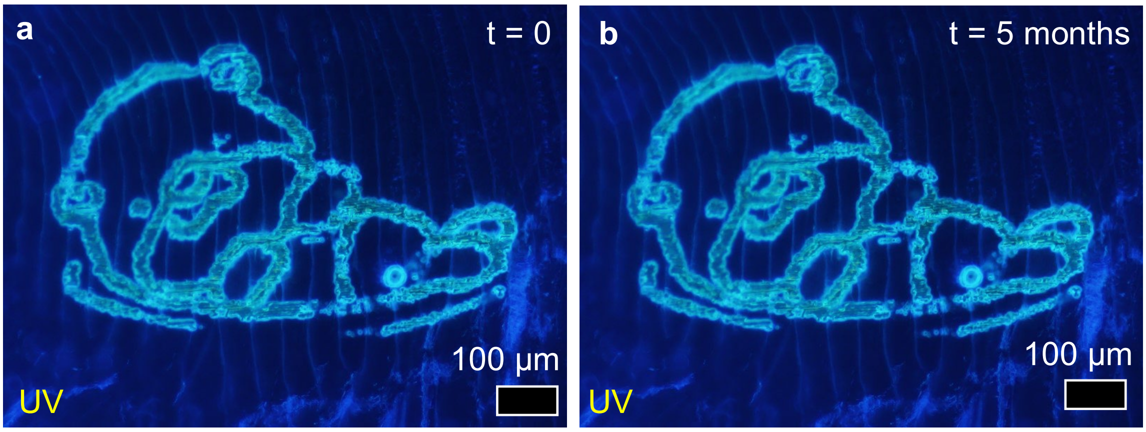

Supplementary Fig. 12. FM images taken of the same sample at **a** t = 0 s and **b** t = 5 months after the Otter design is engraved onto the fish scale.

SEM images of the cross-section of a fish scale after treatment with low laser power density (1.27 MW cm^{-2}) and at different patterning speeds.

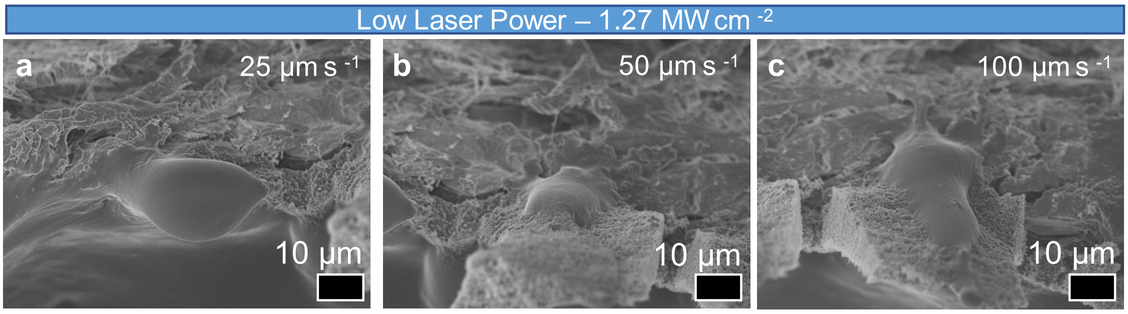

Supplementary Fig. 13. **a** 25 µm s^{-1}, **b** 50 µm s^{-1} and **c** 100 µm s^{-1}.

More heat is imparted by reducing the speed of the laser process, causing the formation of larger areas of denaturation, thus presenting a means for controlled fluorescence.

FM images of a 10^{-8} M droplet of RhB placed on a silicon wafer under different excitation lights.

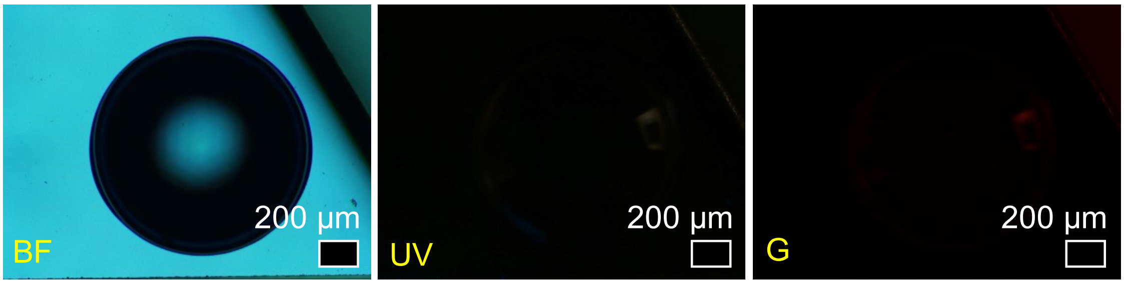

Supplementary Fig. 14. At this concentration, the droplet of RhB emits very dim fluorescent under UV, and a very slightly orange fluorescent is observed under G excitation.

Systematic FM and PL analyses of RhB adsorption efficiency from fish scale heated at different temperatures.

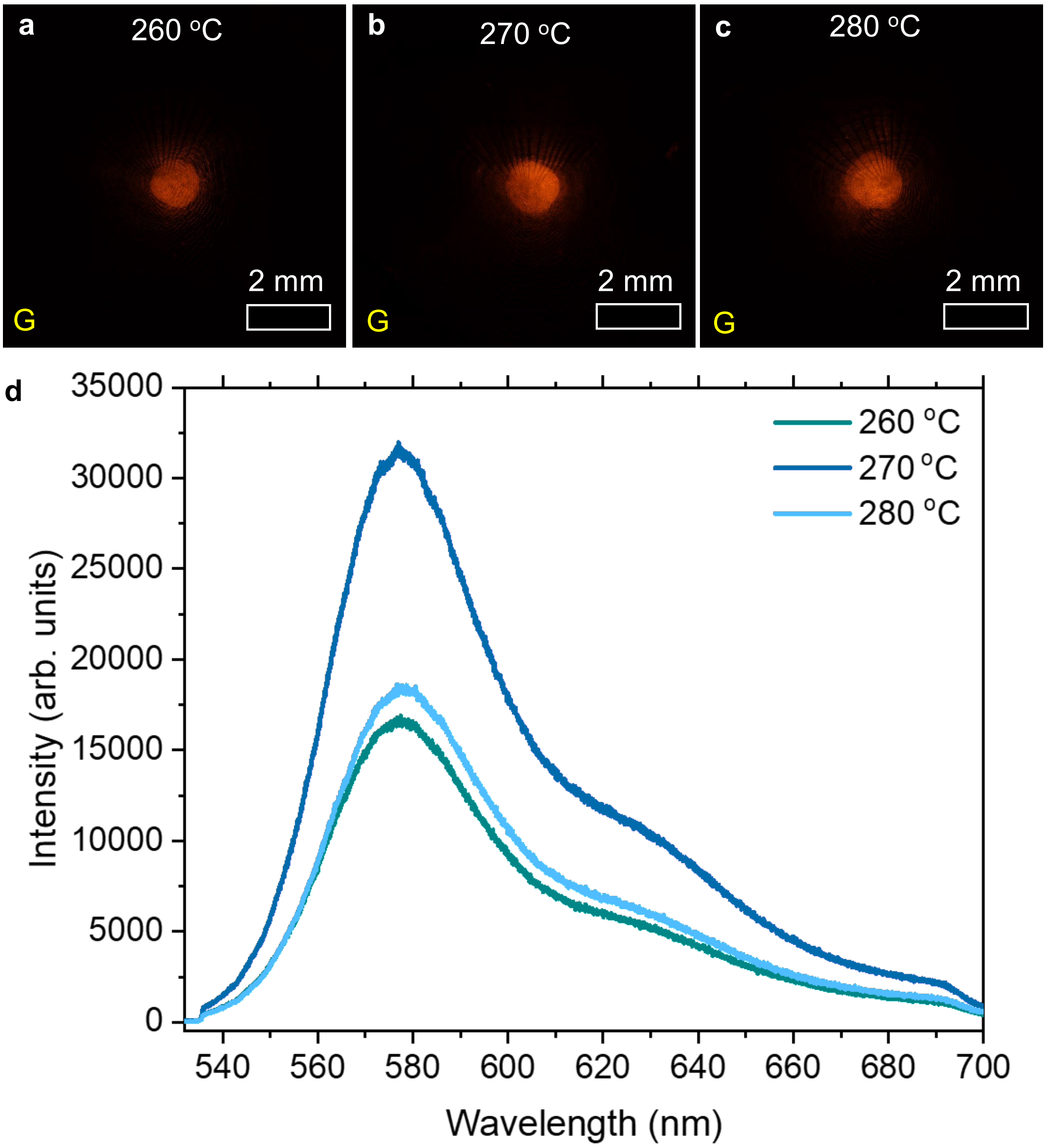

Supplementary Fig. 15. FM imaging under G excitation of fish scale heated at **a** 260 °C, **b** 270 °C and **c** 280 °C after rinsing off the RhB droplet. **d** PL spectra obtained from RhB-treated regions shown in (**a-c**).

Adsorption and recovery of heated fish scale from MB adsorption.

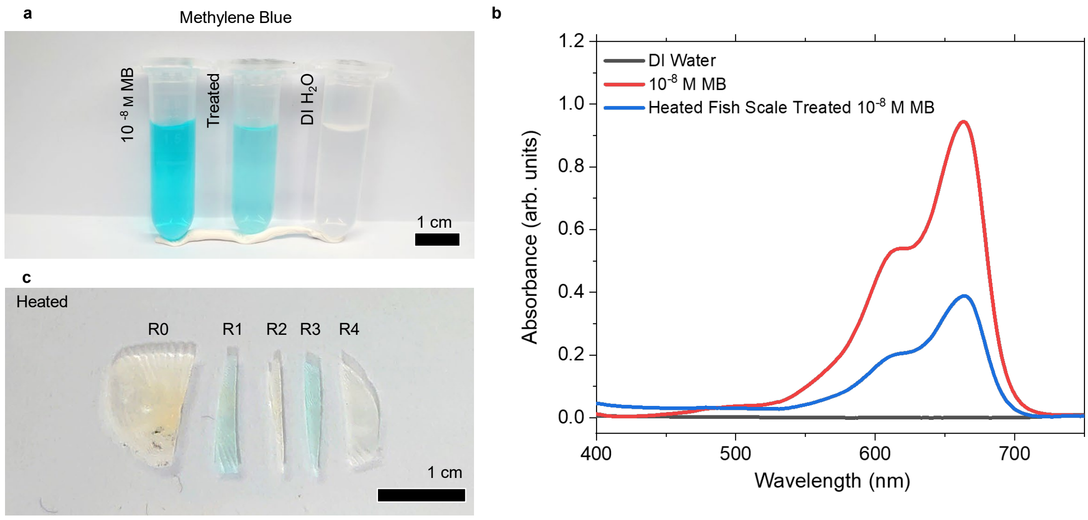

Supplementary Fig. 16. **a** Optical image and **b** UV-Vis spectra of 10^{-8} M of MB solution before and after being treated with 120 mg of heated fish scales with reference to DI water. **c** Recoverability of heated fish scale after repeated soaking and sonicating in DI water.
